# Supplementary figures and images for: Connecting Health and Technology: Validation of Instant Messaging for Use as Diabetes Mellitus Control Strategy in Older Brazilian Adults
Source: Int J Environ Res Public Health. 2025 Feb 14;22(2):282. doi: 10.3390/ijerph22020282 (PMC11855891; doi:10.3390/ijerph22020282)

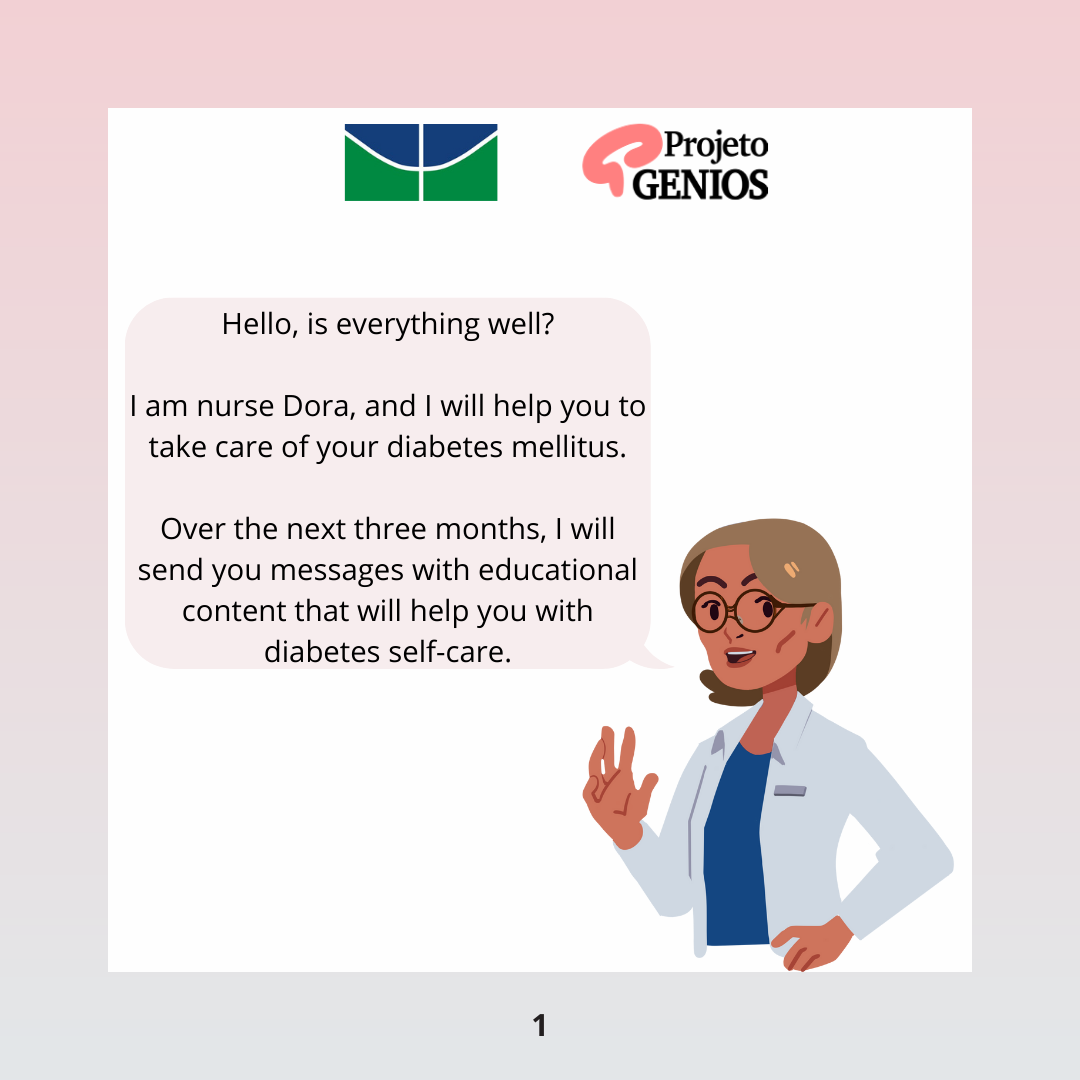

Supplement: Supplementary file 1 [file ijerph-22-00282-s001.zip › Instante Messages English/1.png]

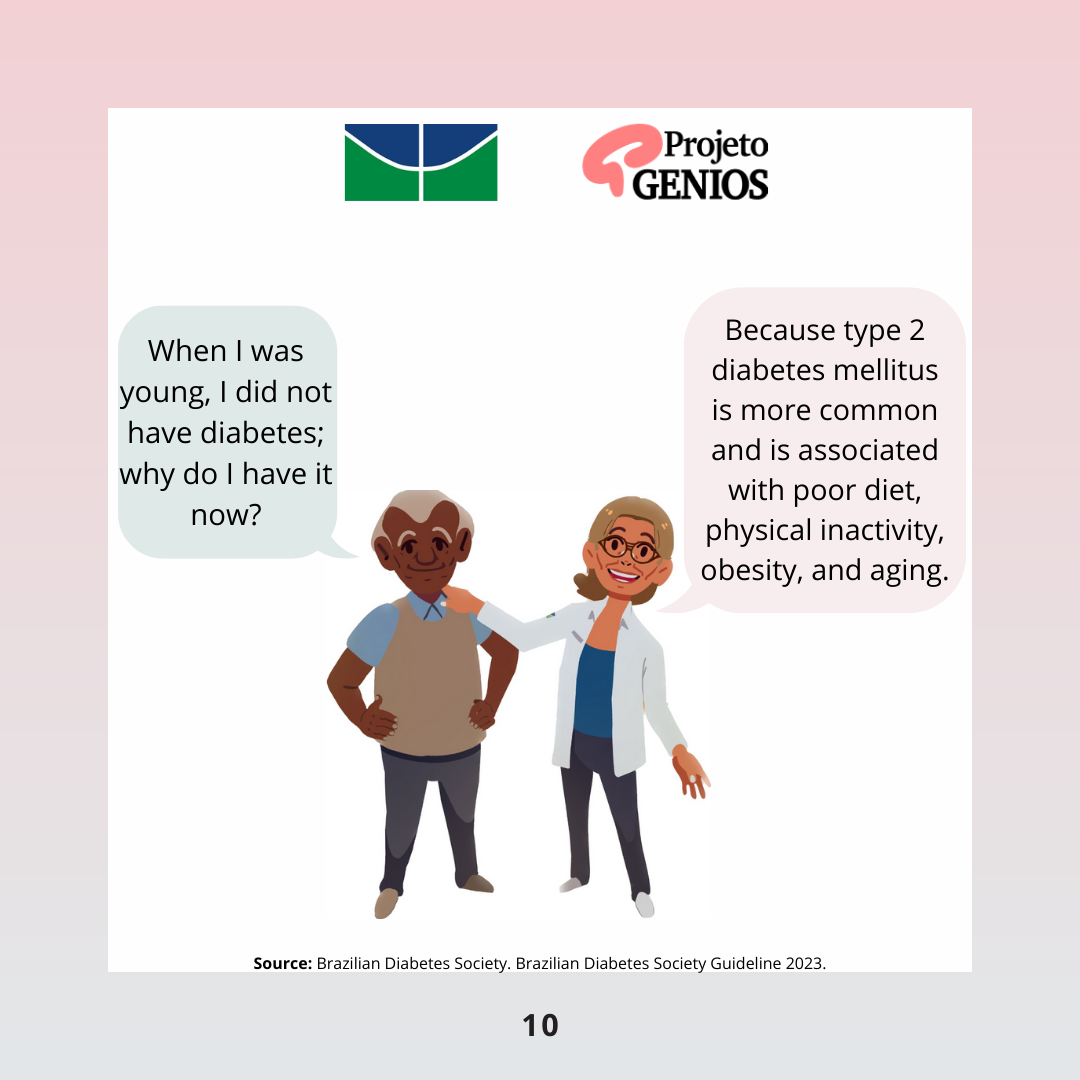

Supplement: Supplementary file 1 [file ijerph-22-00282-s001.zip › Instante Messages English/10.png]

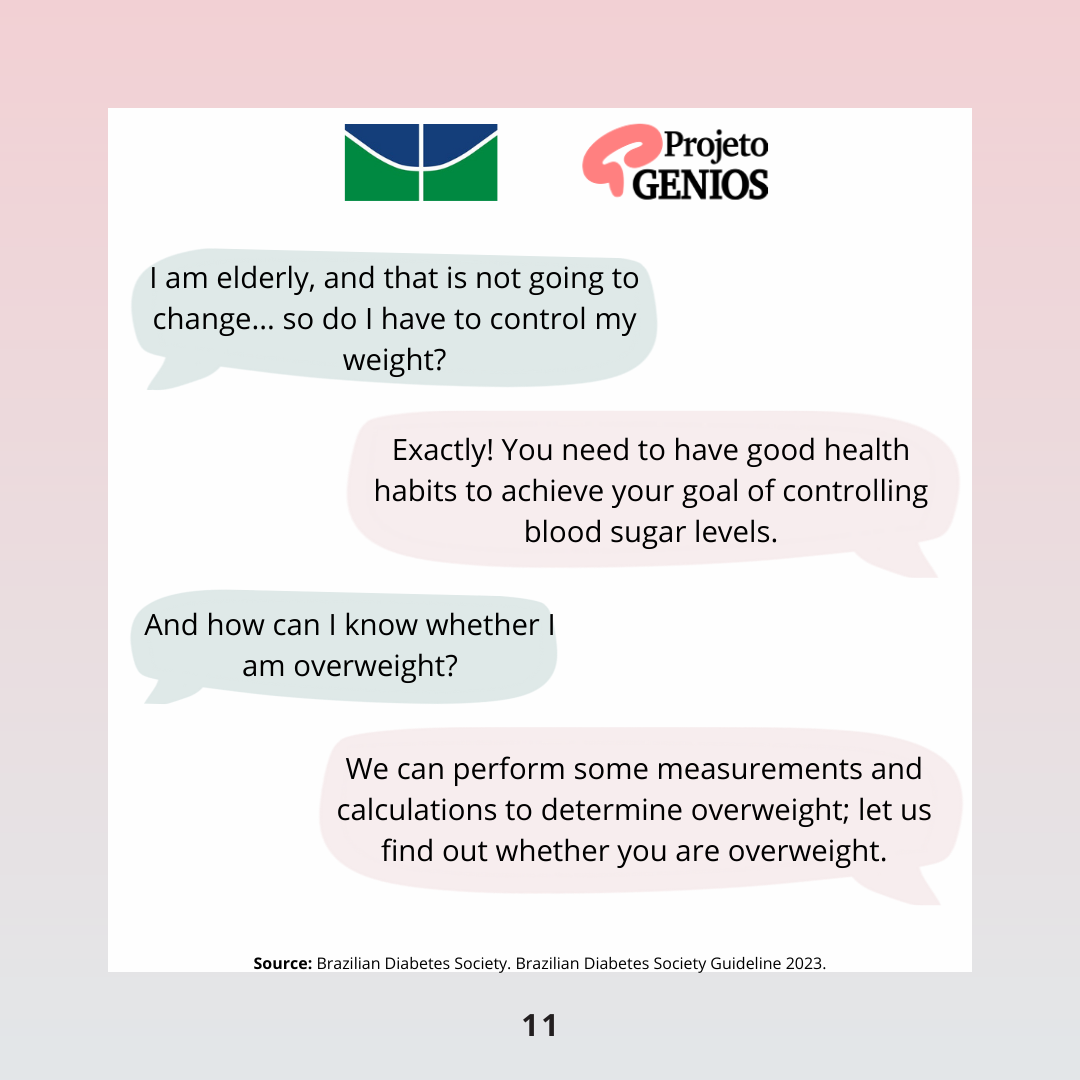

Supplement: Supplementary file 1 [file ijerph-22-00282-s001.zip › Instante Messages English/11.png]

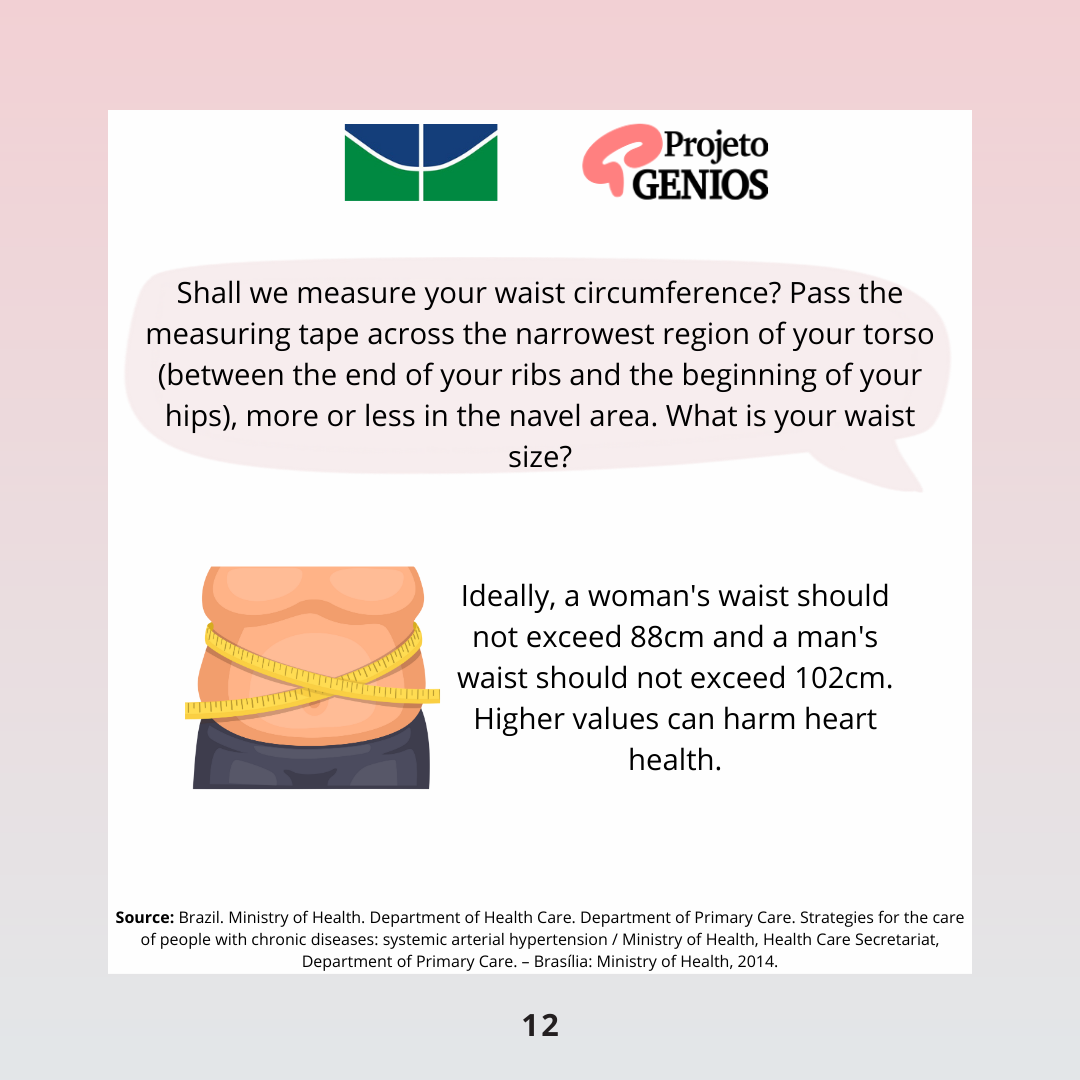

Supplement: Supplementary file 1 [file ijerph-22-00282-s001.zip › Instante Messages English/12.png]

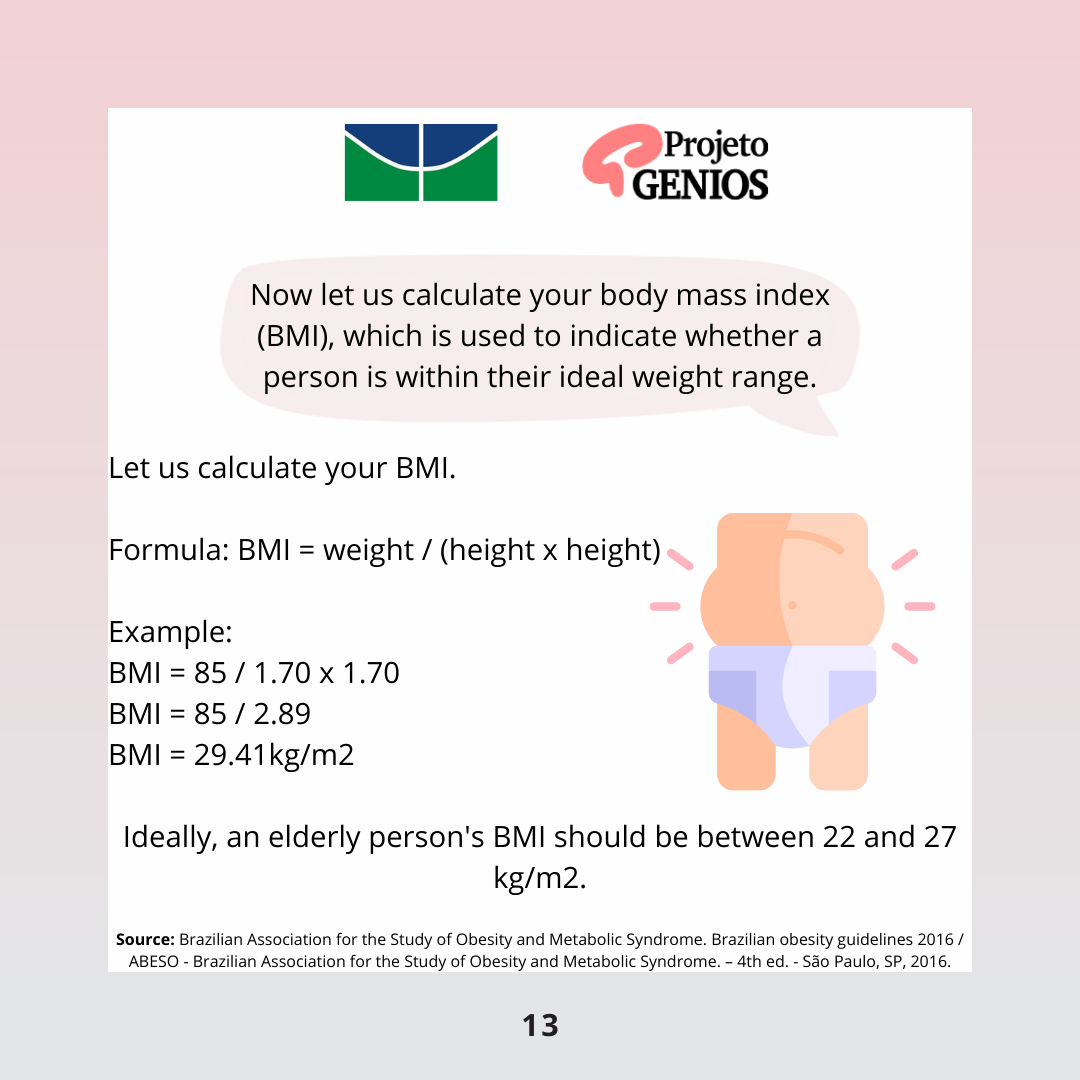

Supplement: Supplementary file 1 [file ijerph-22-00282-s001.zip › Instante Messages English/13.png]

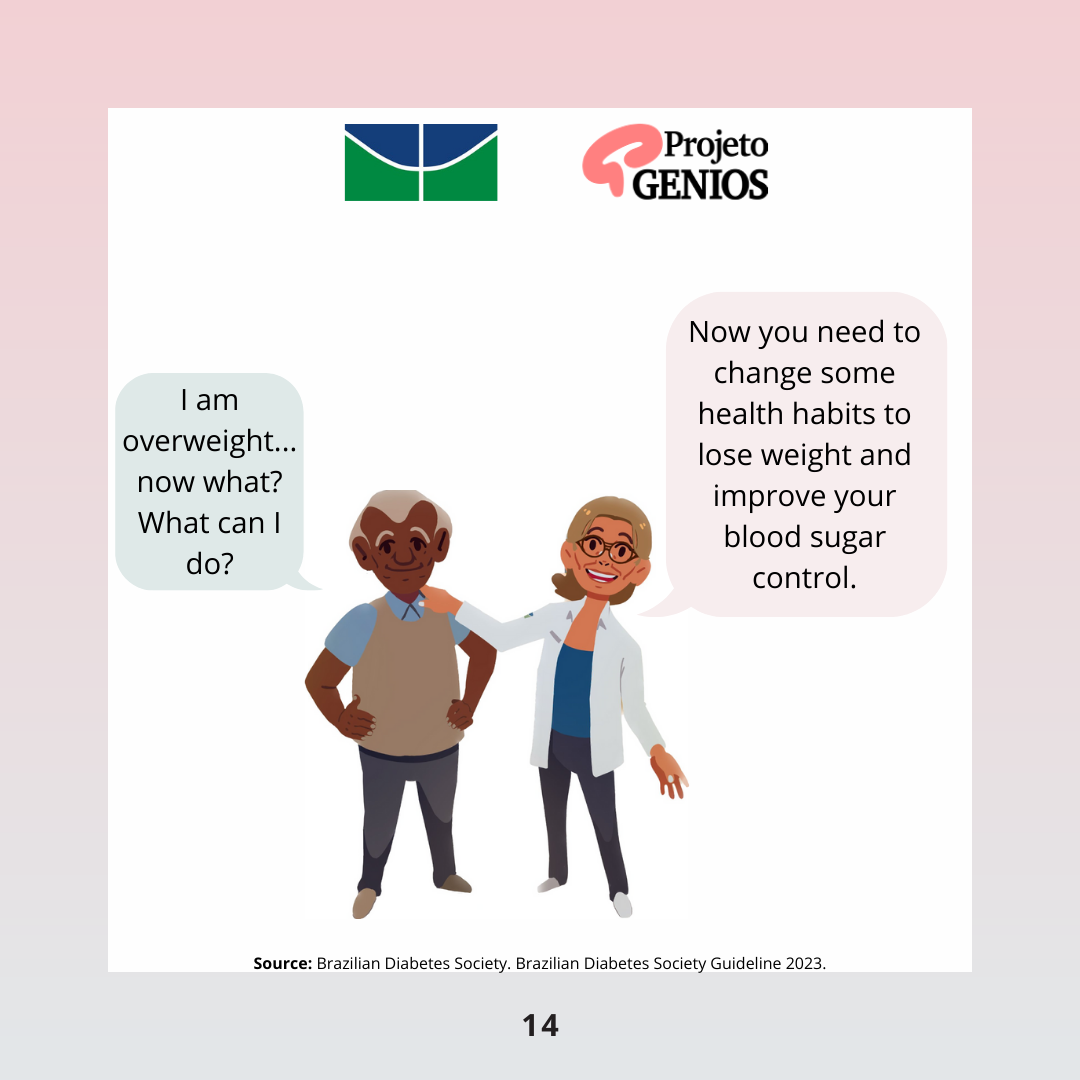

Supplement: Supplementary file 1 [file ijerph-22-00282-s001.zip › Instante Messages English/14.png]

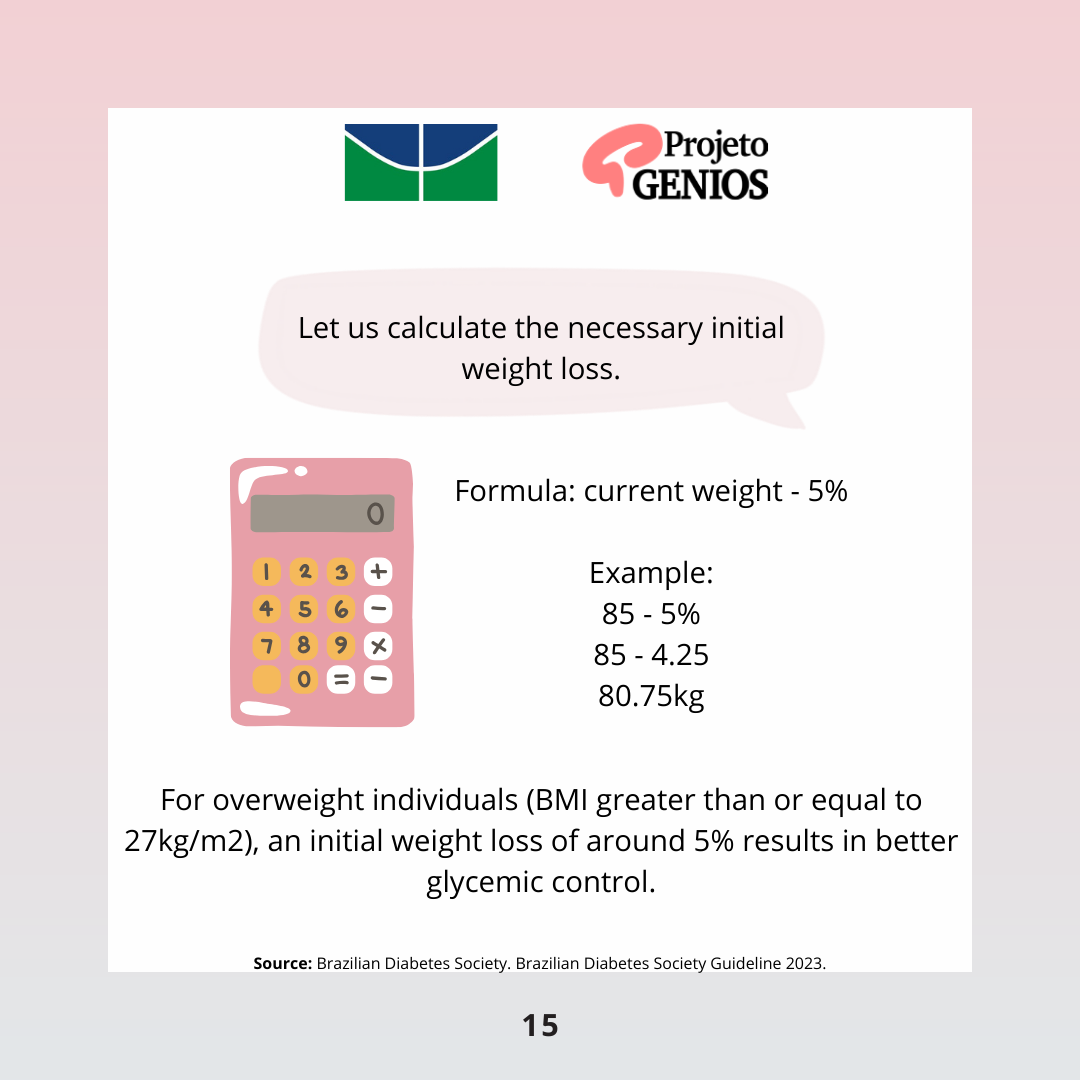

Supplement: Supplementary file 1 [file ijerph-22-00282-s001.zip › Instante Messages English/15.png]

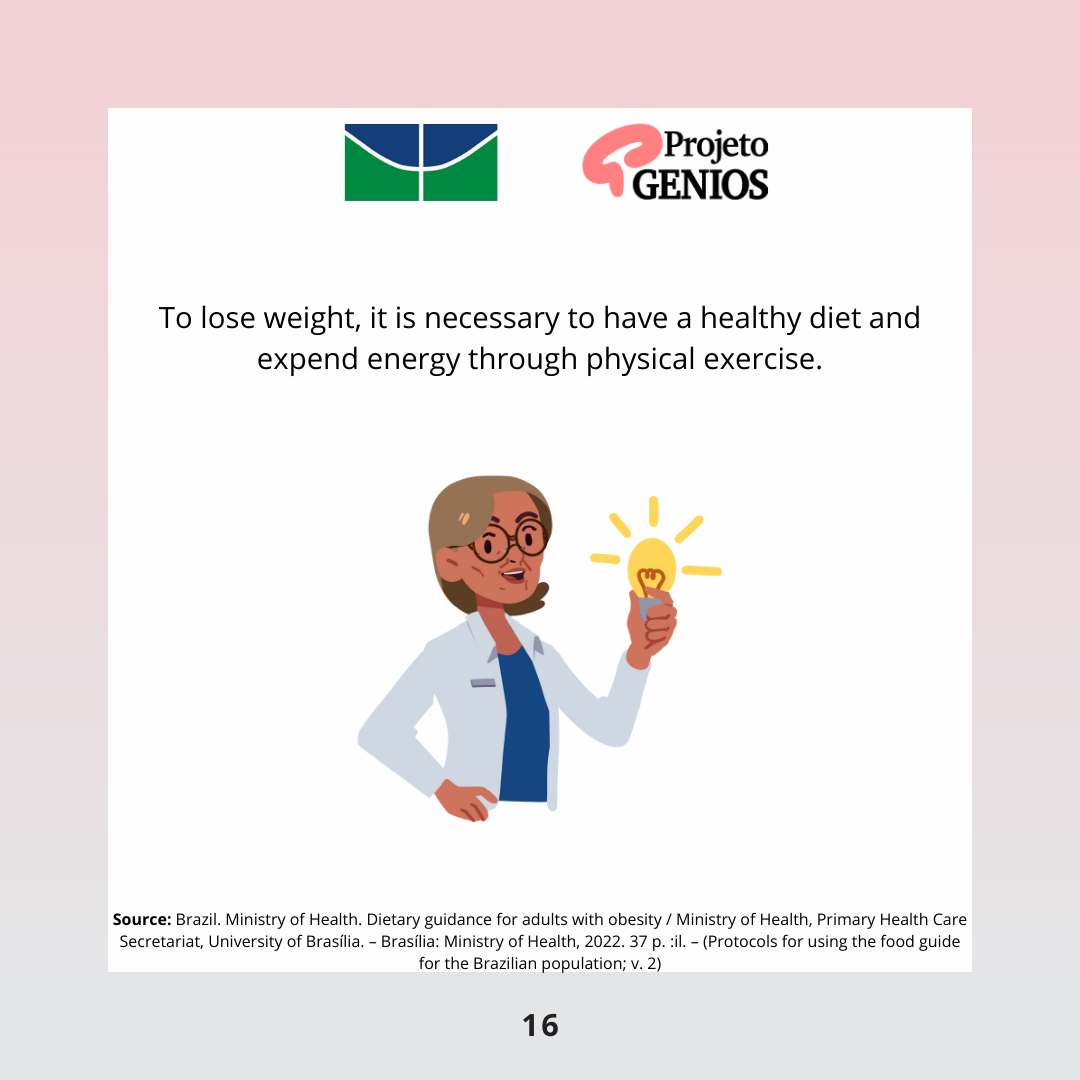

Supplement: Supplementary file 1 [file ijerph-22-00282-s001.zip › Instante Messages English/16.png]

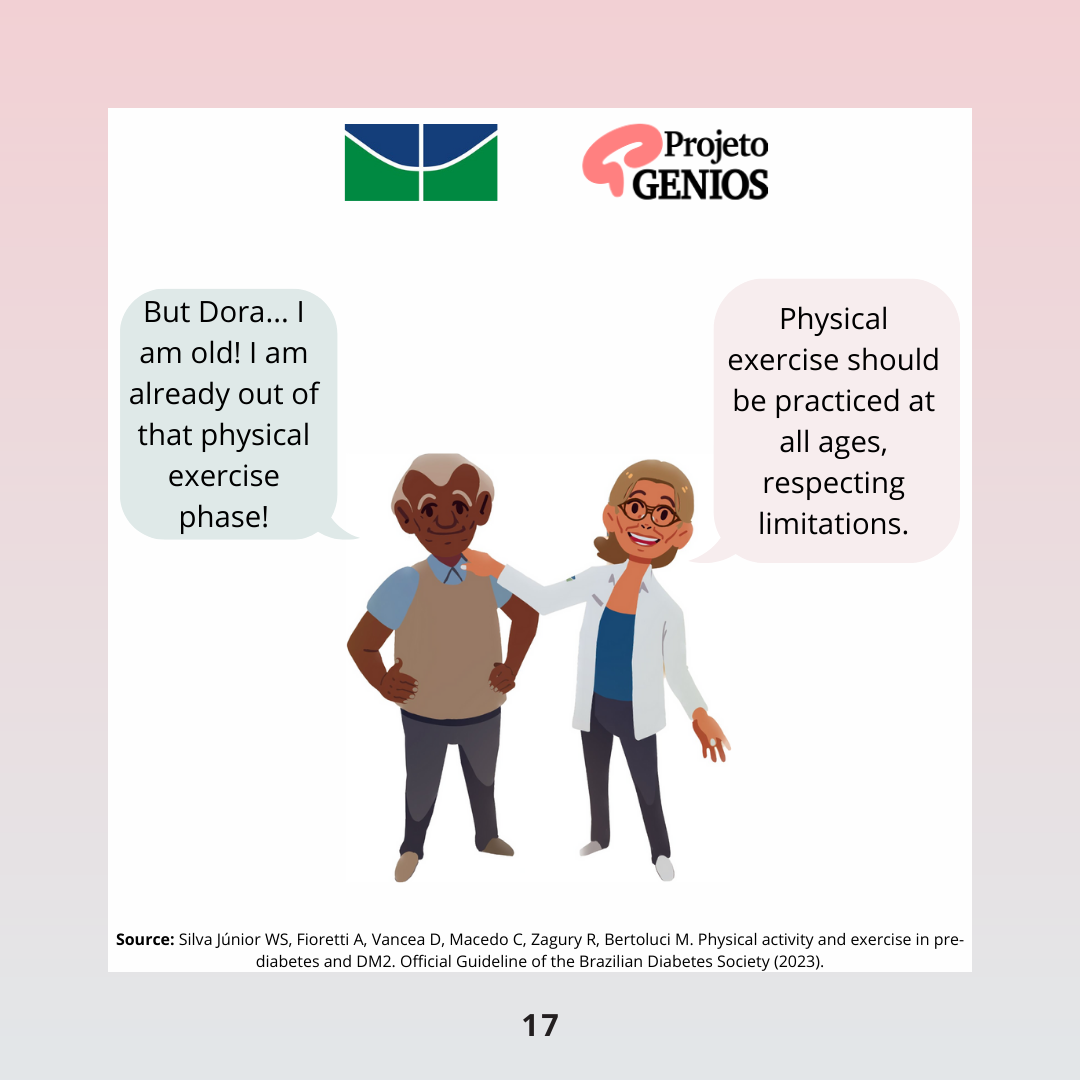

Supplement: Supplementary file 1 [file ijerph-22-00282-s001.zip › Instante Messages English/17.png]

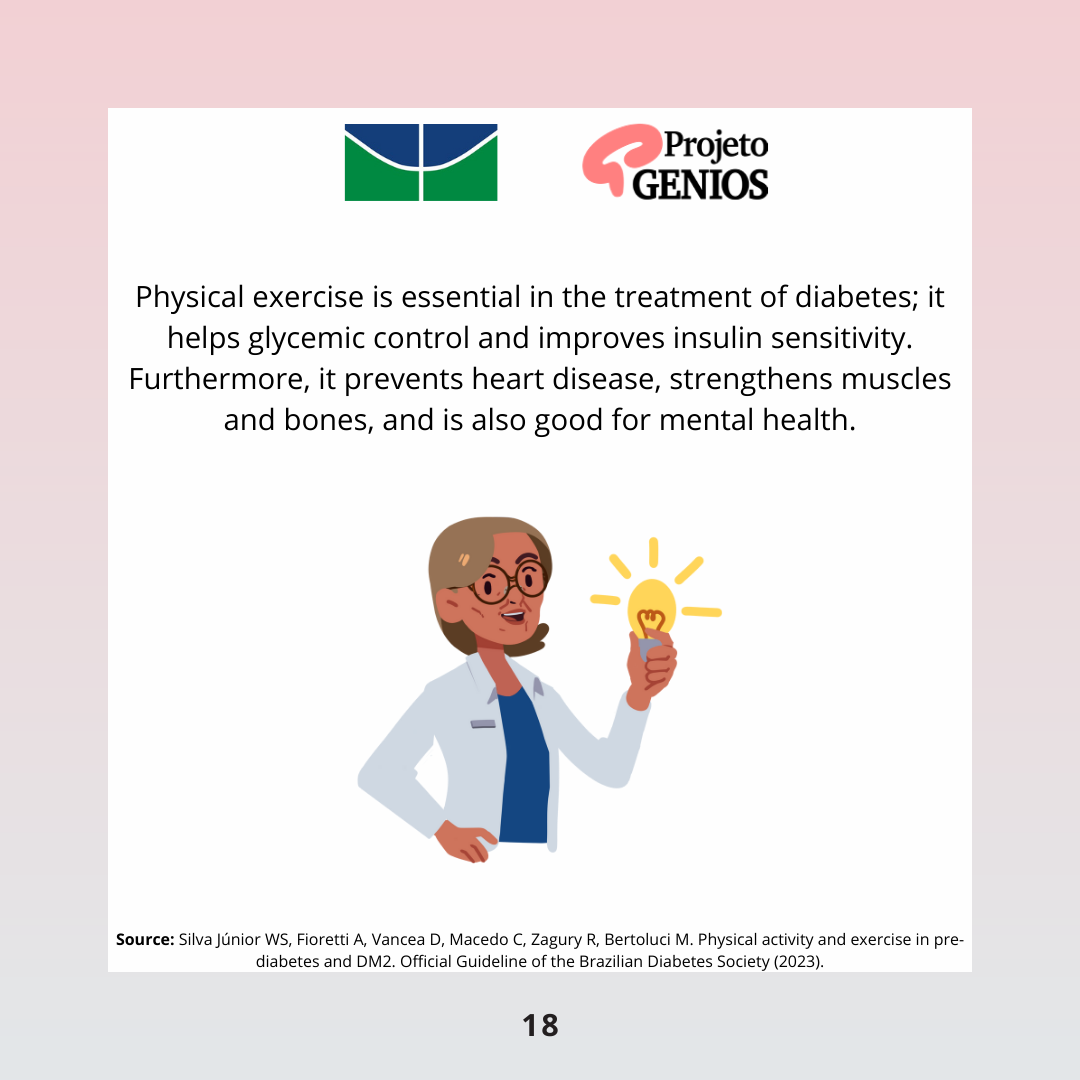

Supplement: Supplementary file 1 [file ijerph-22-00282-s001.zip › Instante Messages English/18.png]

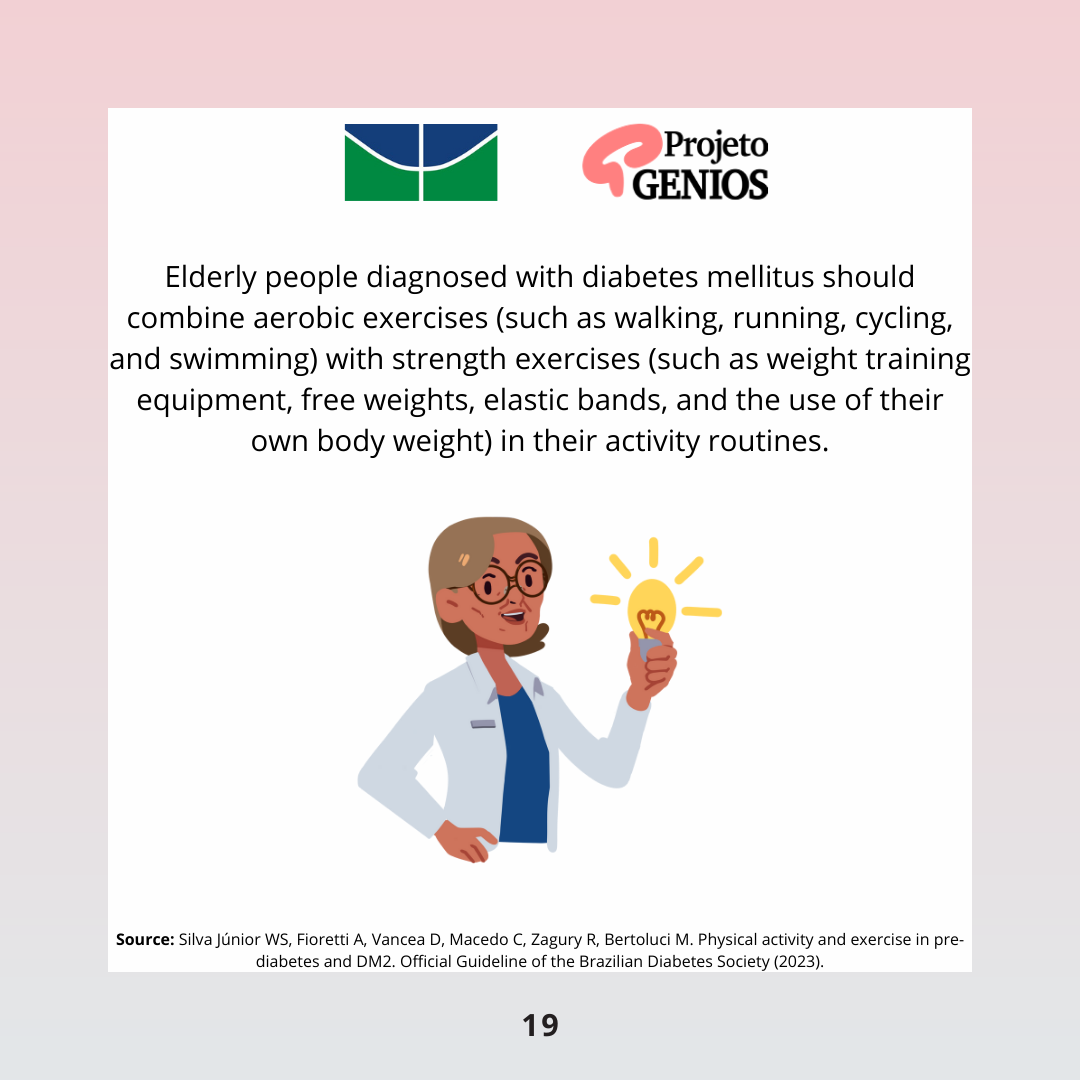

Supplement: Supplementary file 1 [file ijerph-22-00282-s001.zip › Instante Messages English/19.png]

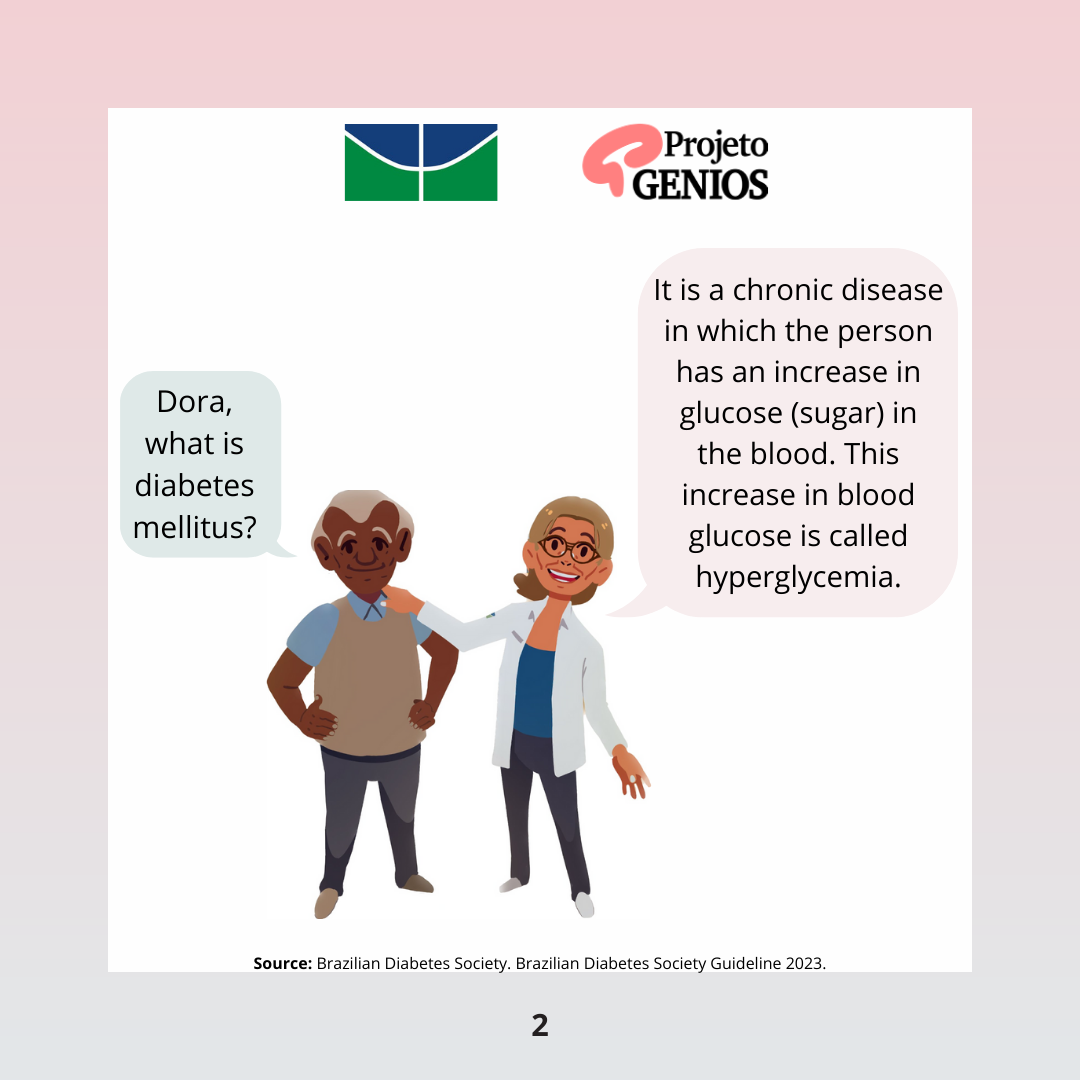

Supplement: Supplementary file 1 [file ijerph-22-00282-s001.zip › Instante Messages English/2.png]

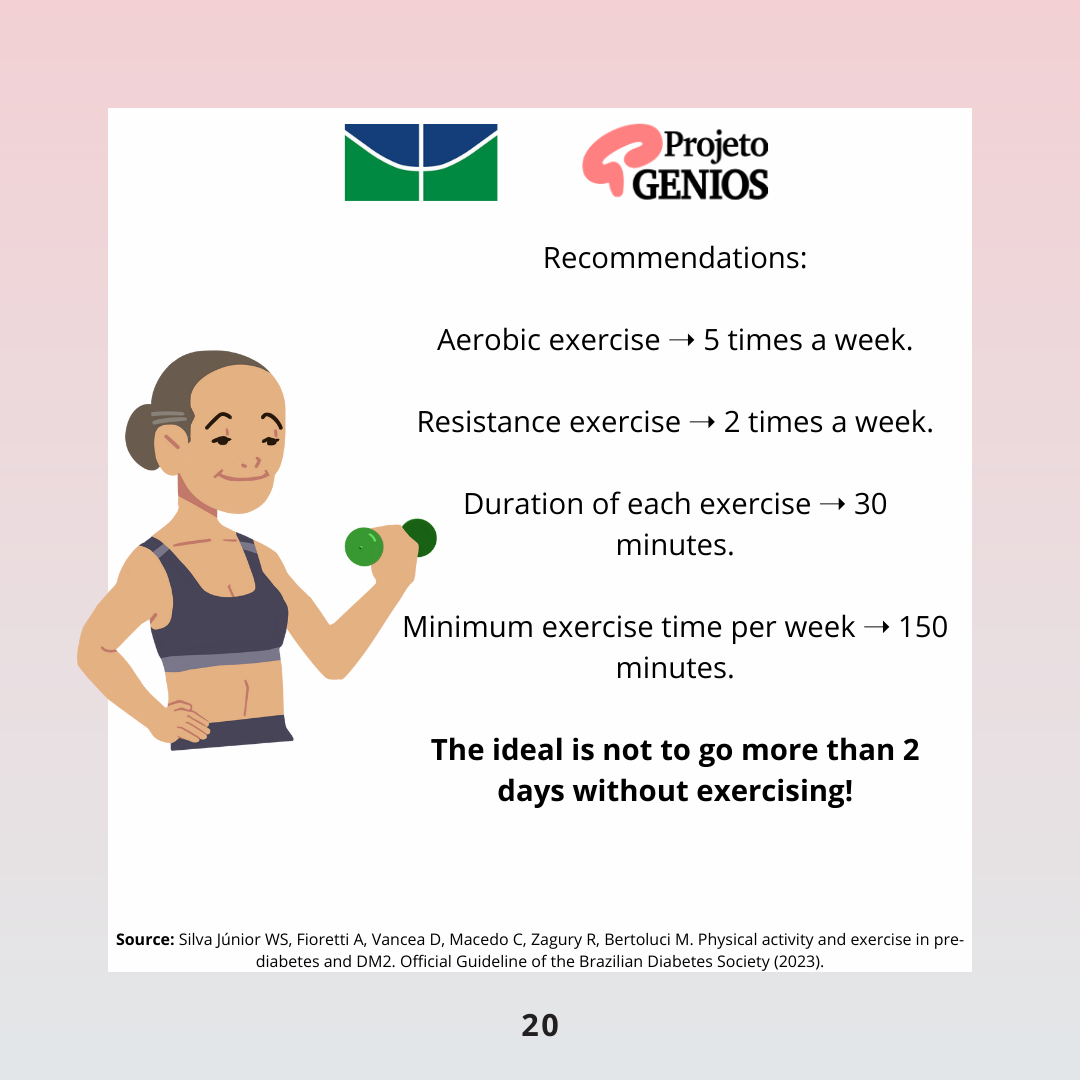

Supplement: Supplementary file 1 [file ijerph-22-00282-s001.zip › Instante Messages English/20.png]

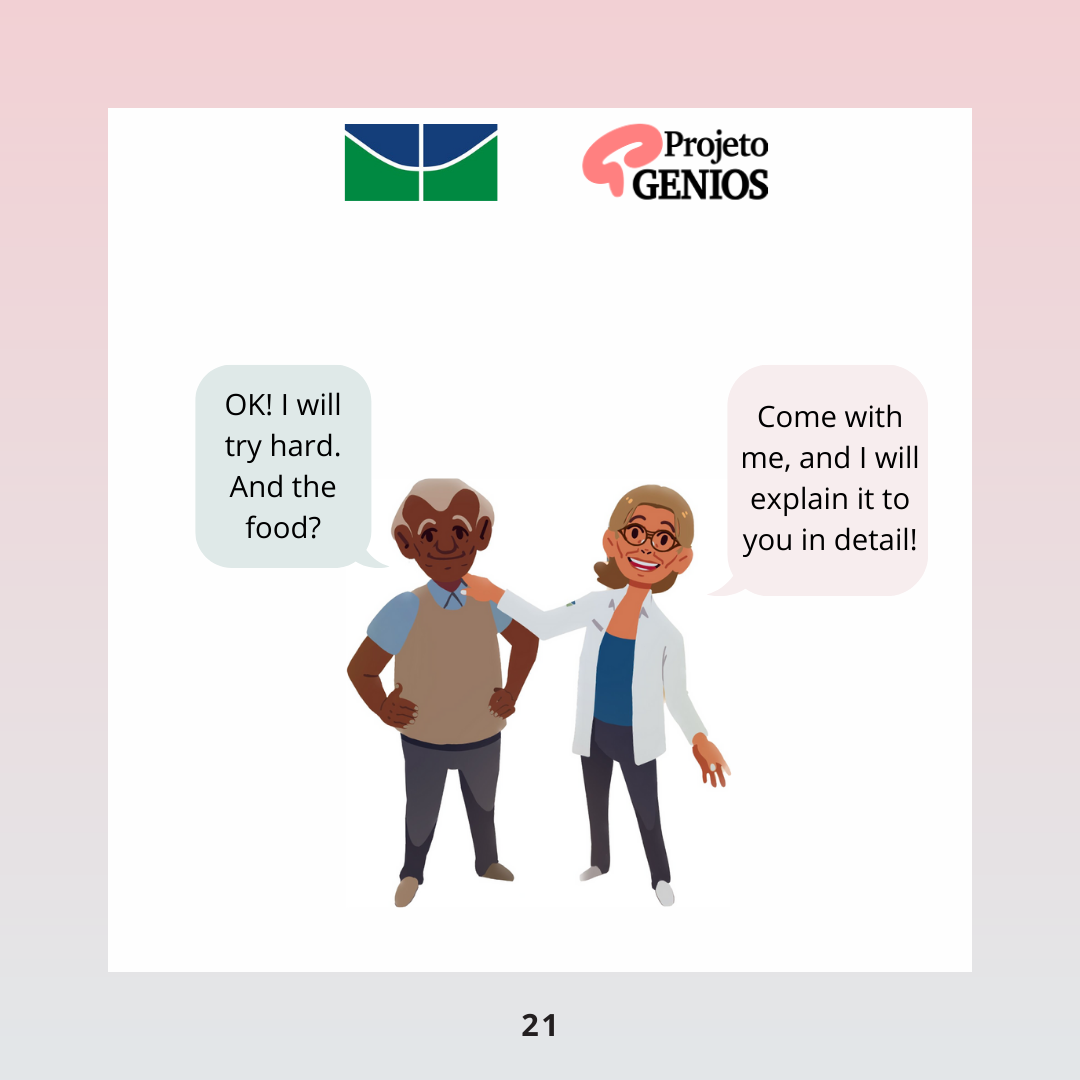

Supplement: Supplementary file 1 [file ijerph-22-00282-s001.zip › Instante Messages English/21.png]

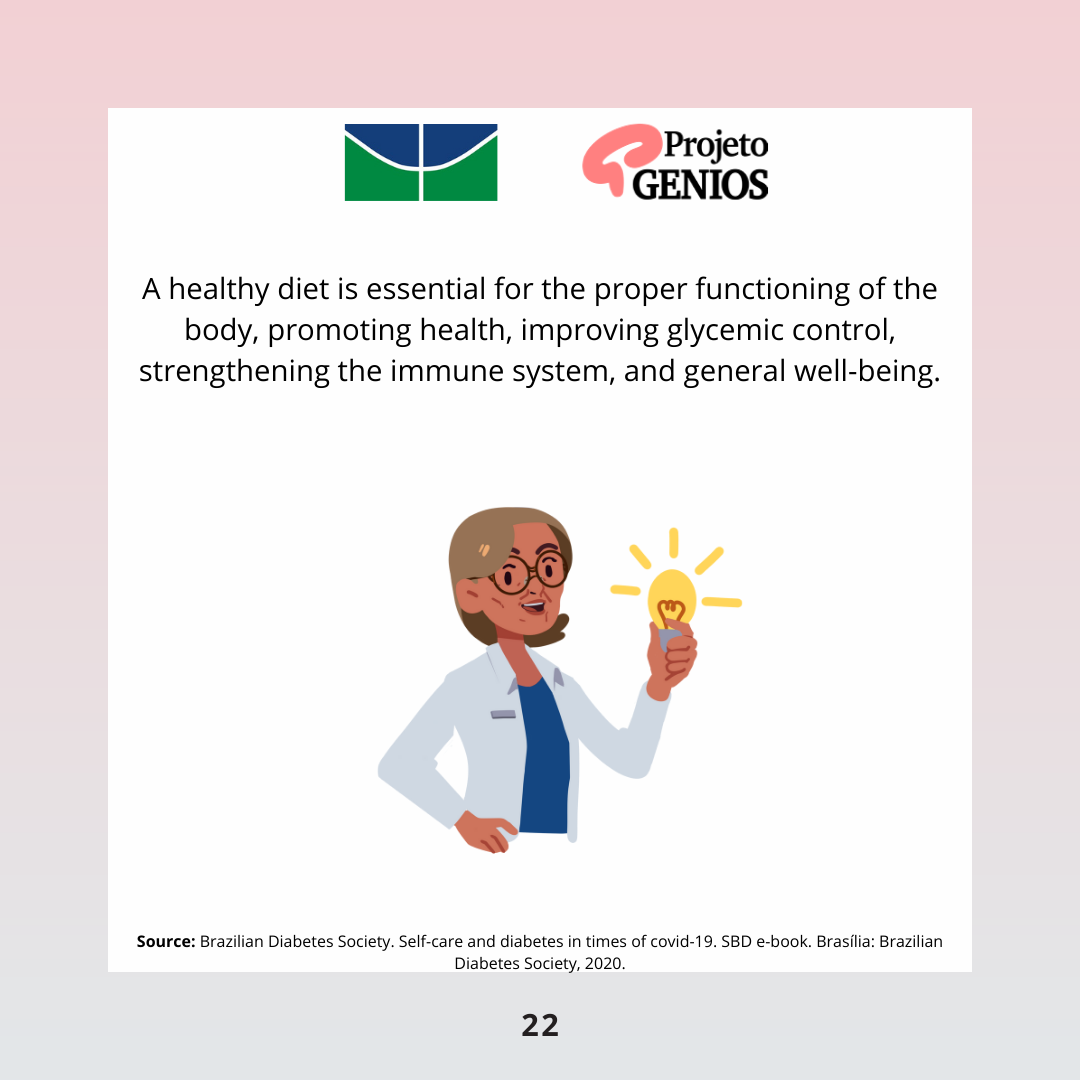

Supplement: Supplementary file 1 [file ijerph-22-00282-s001.zip › Instante Messages English/22.png]

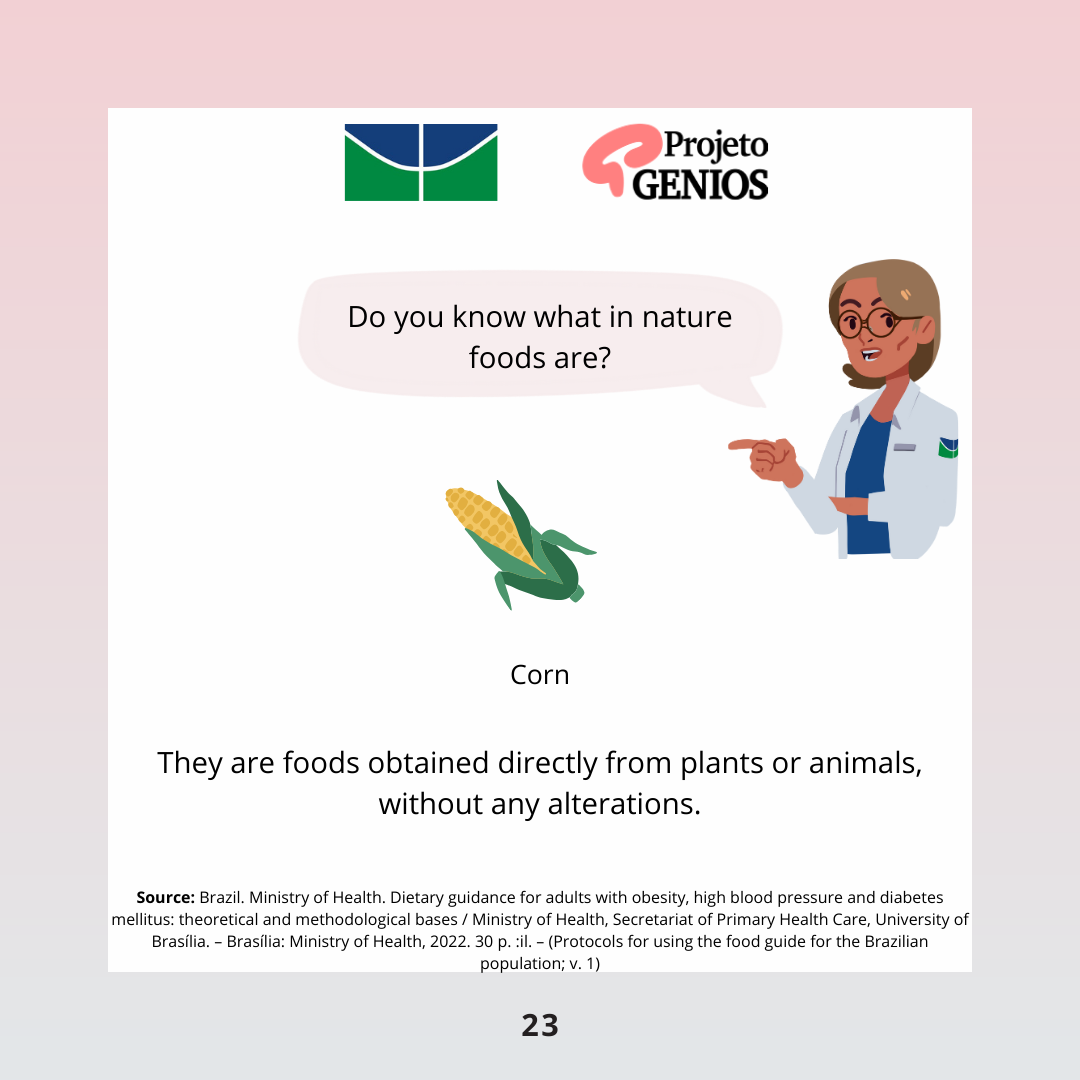

Supplement: Supplementary file 1 [file ijerph-22-00282-s001.zip › Instante Messages English/23.png]

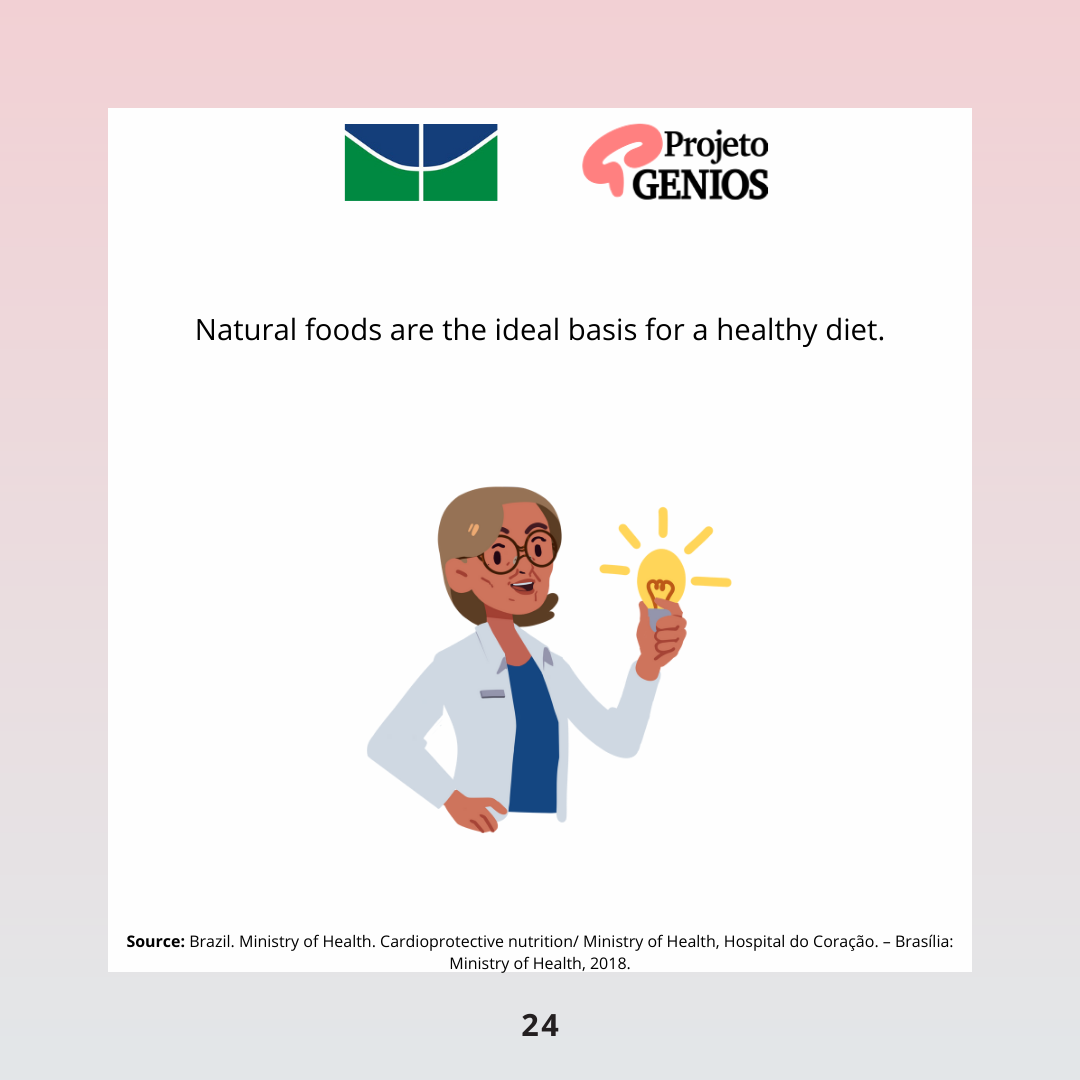

Supplement: Supplementary file 1 [file ijerph-22-00282-s001.zip › Instante Messages English/24.png]

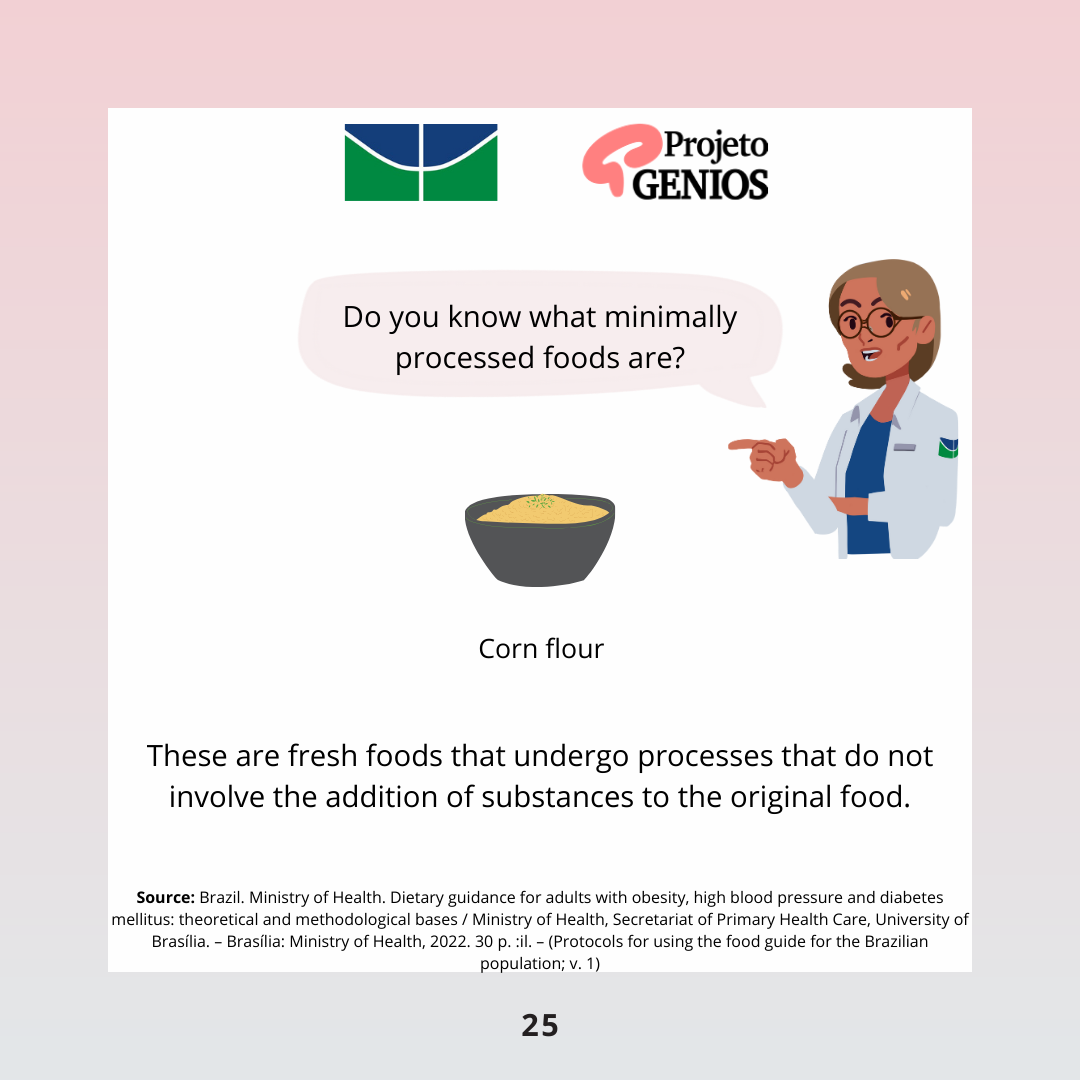

Supplement: Supplementary file 1 [file ijerph-22-00282-s001.zip › Instante Messages English/25.png]

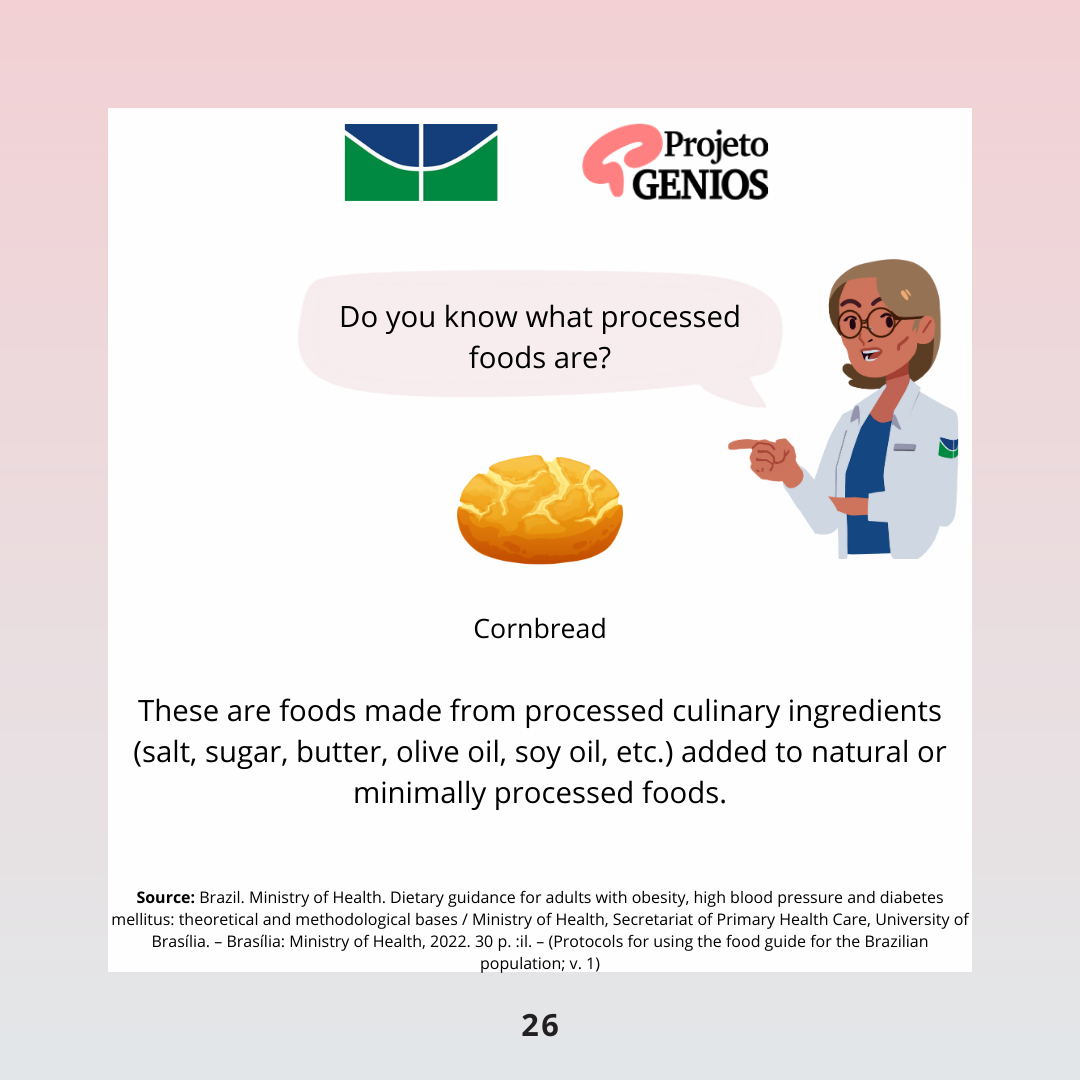

Supplement: Supplementary file 1 [file ijerph-22-00282-s001.zip › Instante Messages English/26.png]

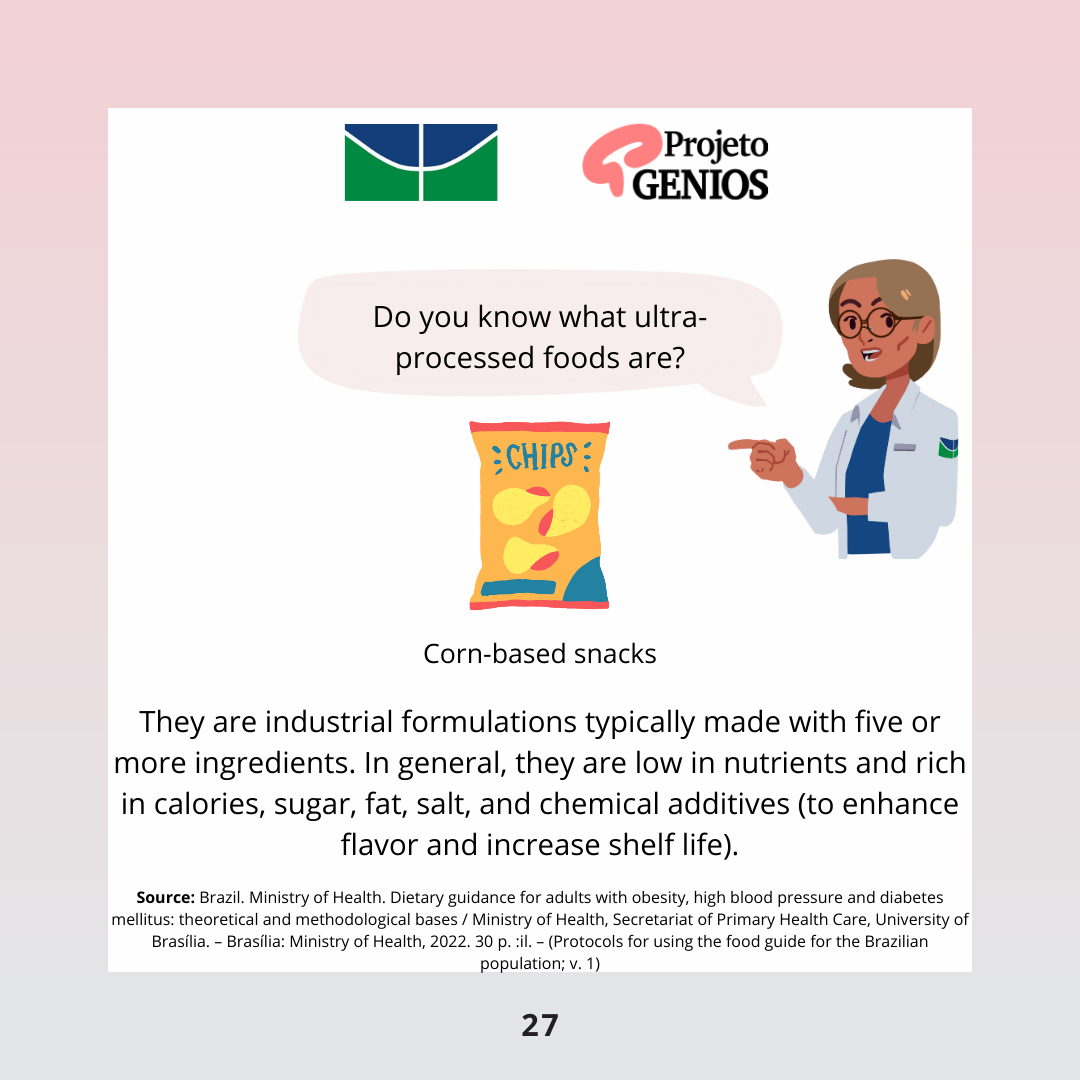

Supplement: Supplementary file 1 [file ijerph-22-00282-s001.zip › Instante Messages English/27.png]

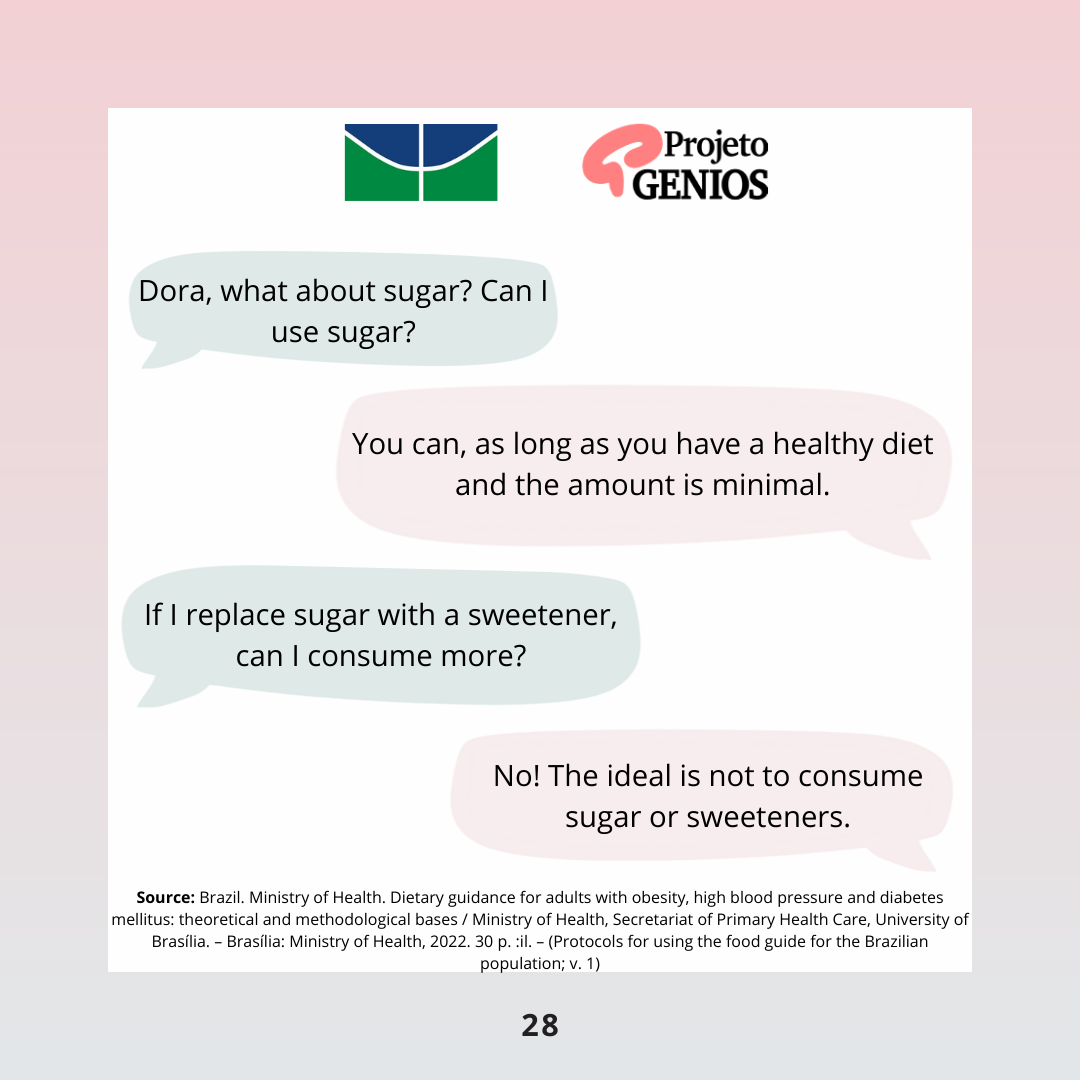

Supplement: Supplementary file 1 [file ijerph-22-00282-s001.zip › Instante Messages English/28.png]

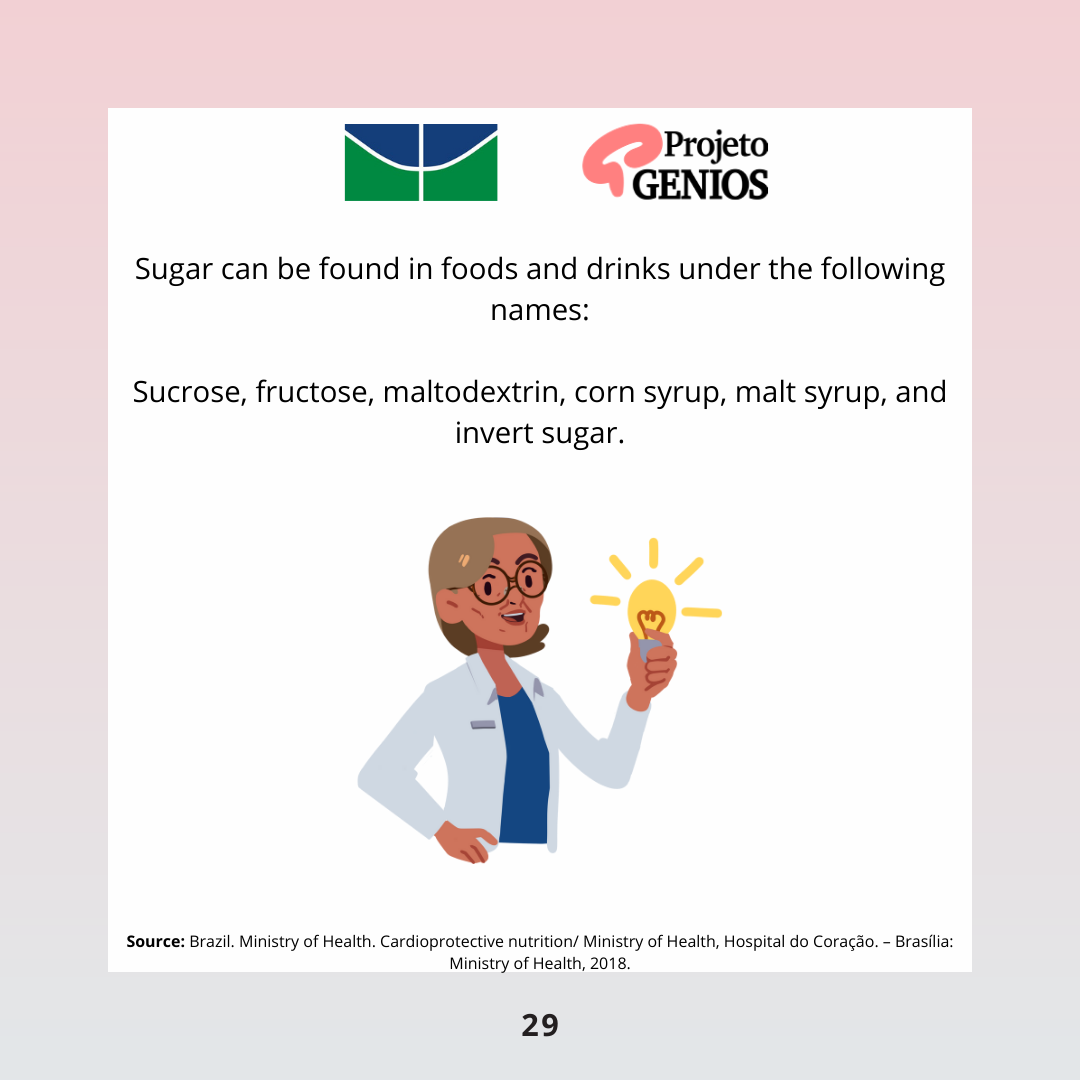

Supplement: Supplementary file 1 [file ijerph-22-00282-s001.zip › Instante Messages English/29.png]

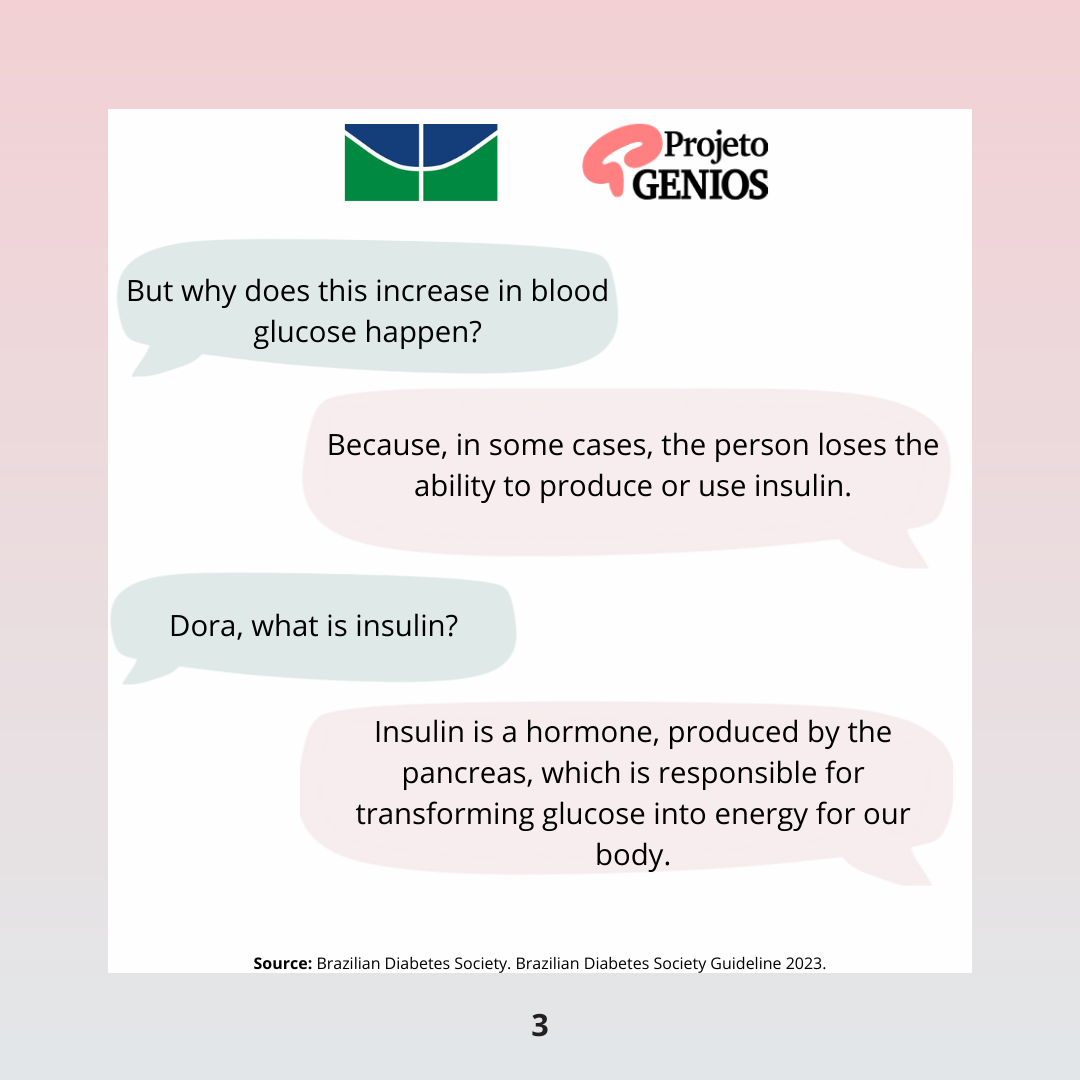

Supplement: Supplementary file 1 [file ijerph-22-00282-s001.zip › Instante Messages English/3.png]

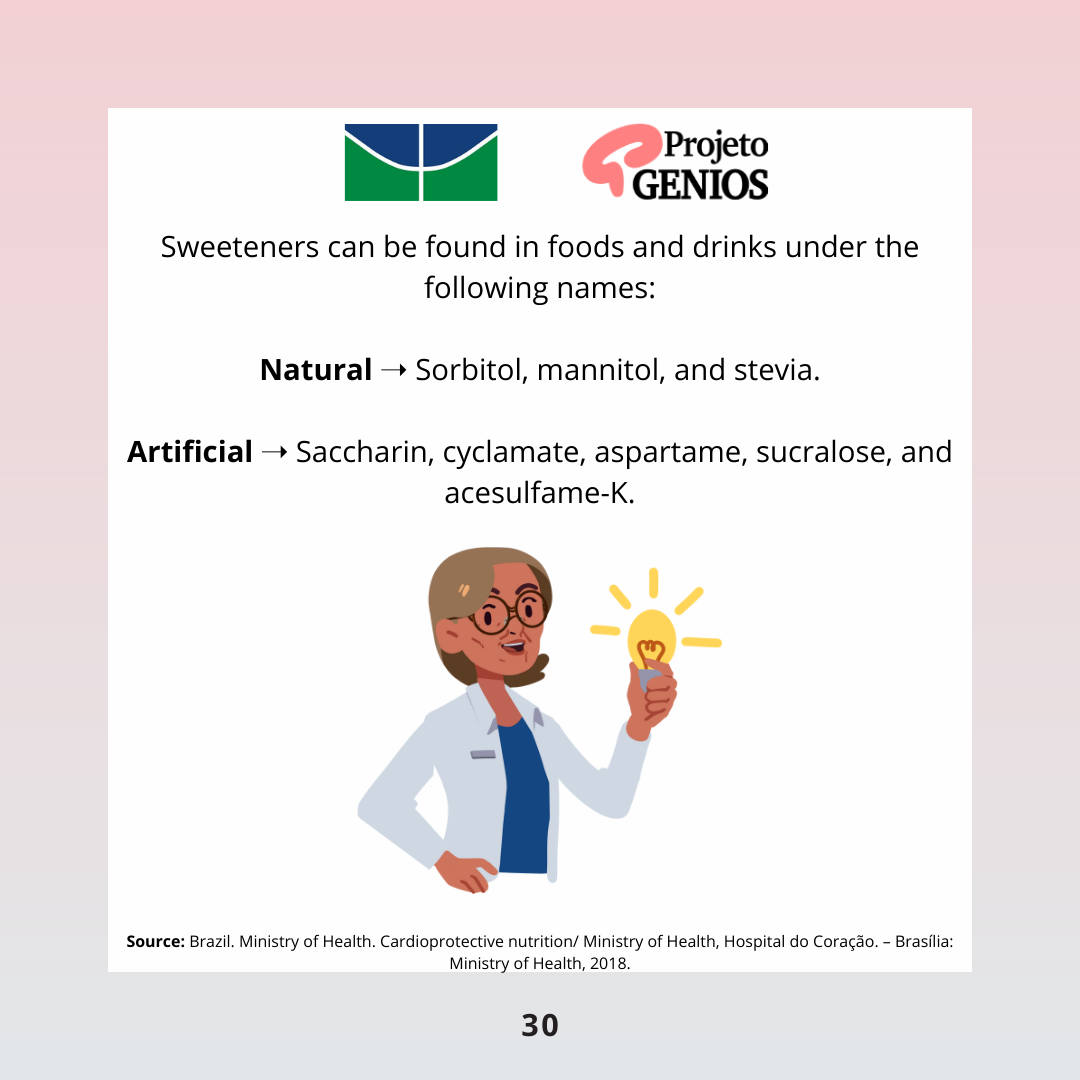

Supplement: Supplementary file 1 [file ijerph-22-00282-s001.zip › Instante Messages English/30.png]

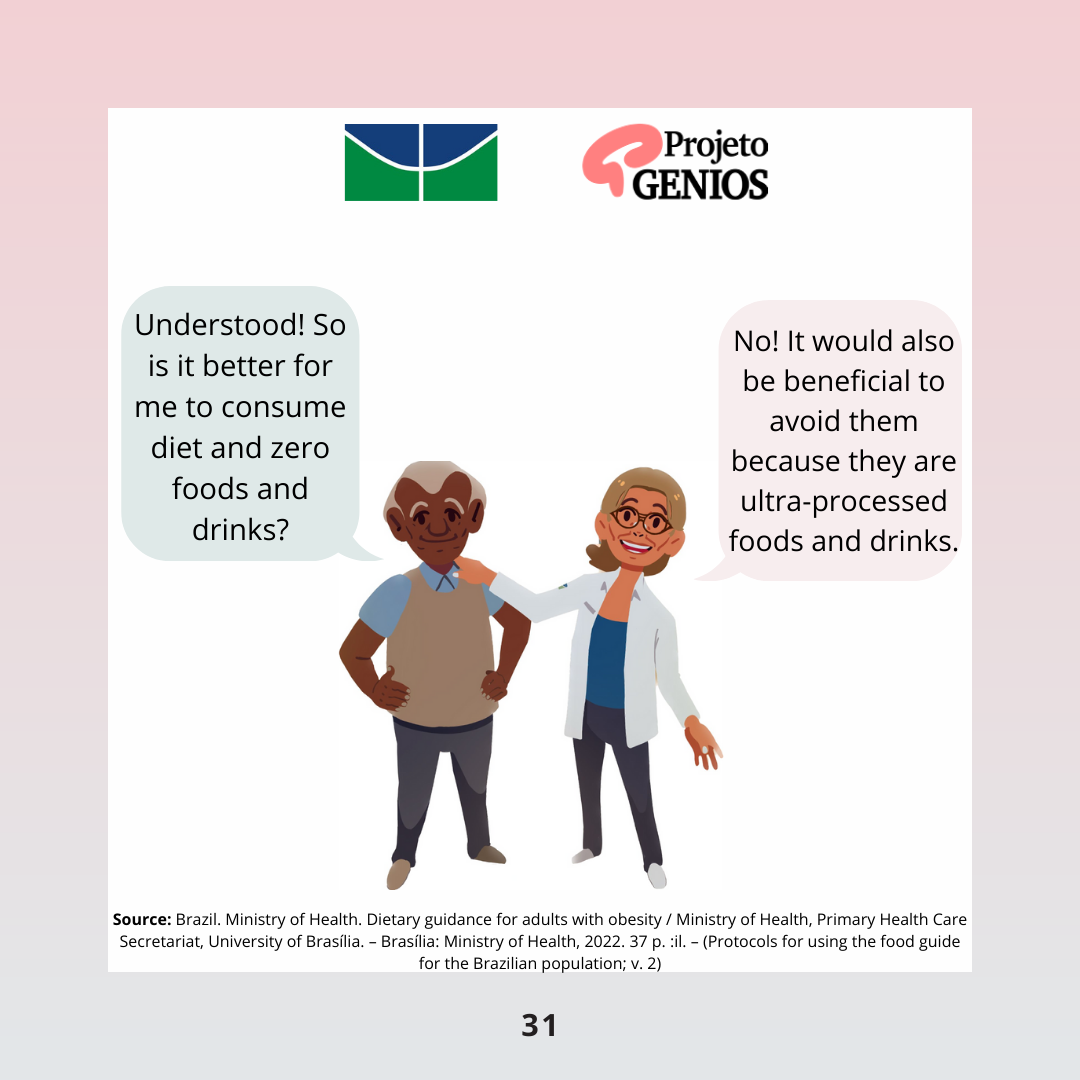

Supplement: Supplementary file 1 [file ijerph-22-00282-s001.zip › Instante Messages English/31.png]

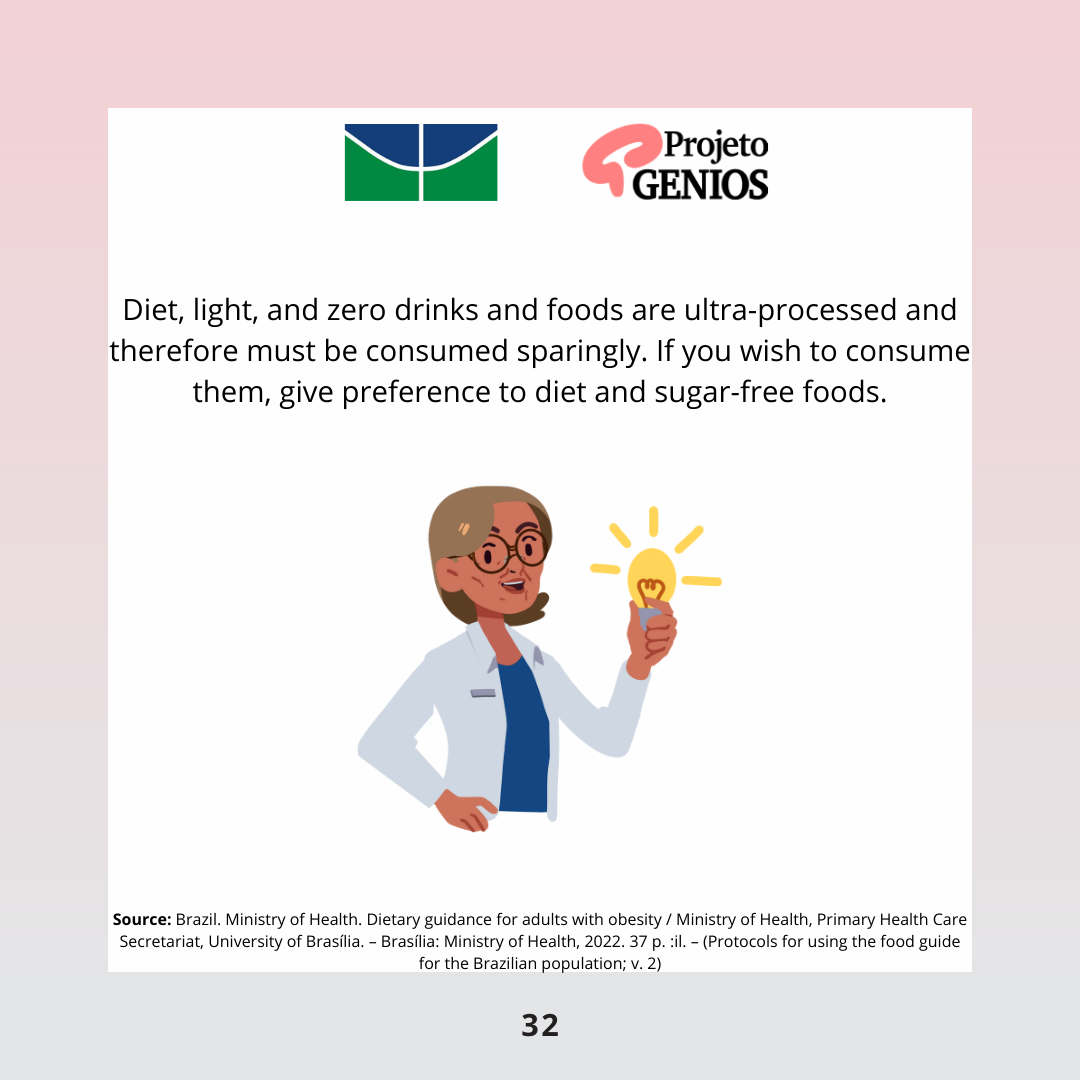

Supplement: Supplementary file 1 [file ijerph-22-00282-s001.zip › Instante Messages English/32.png]

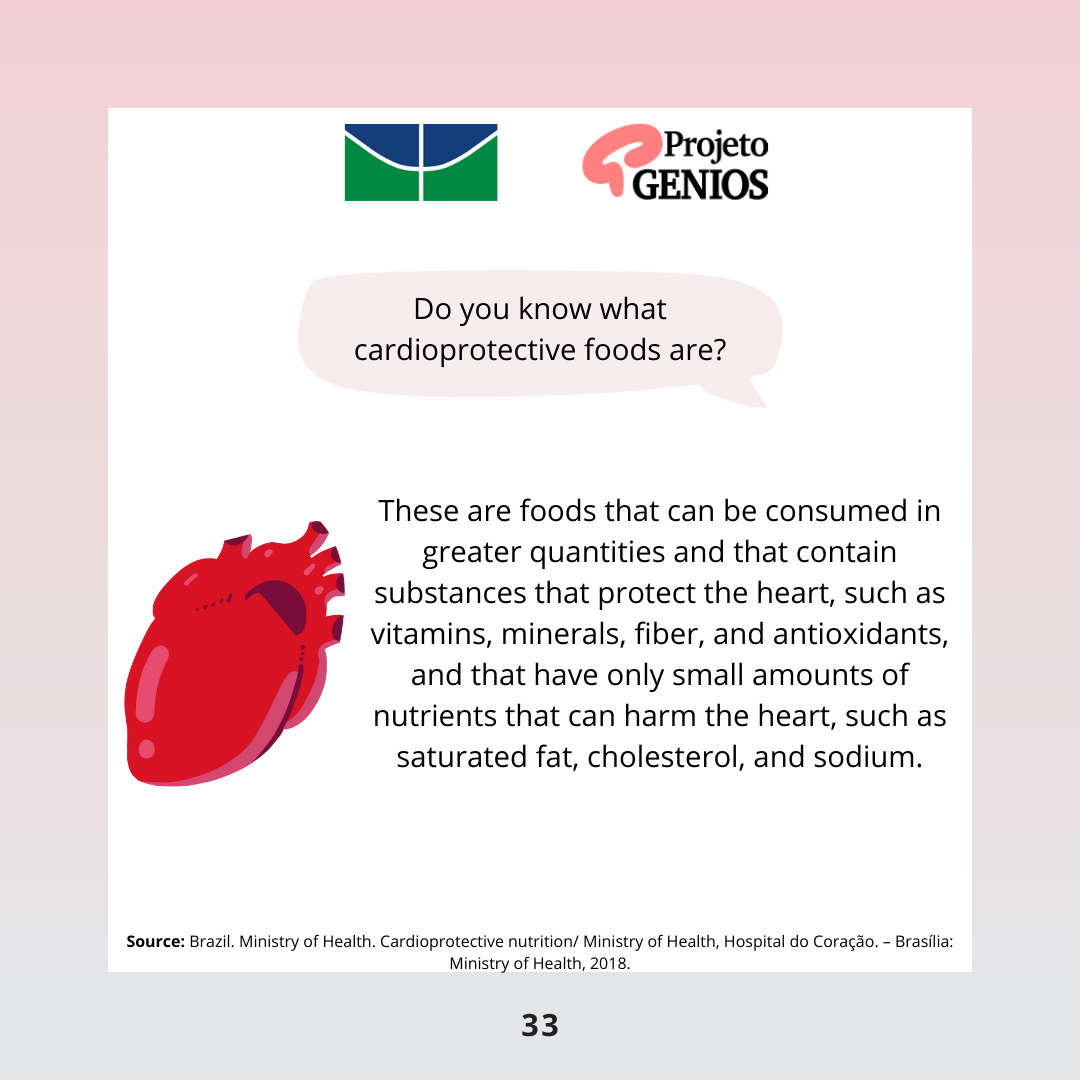

Supplement: Supplementary file 1 [file ijerph-22-00282-s001.zip › Instante Messages English/33.png]

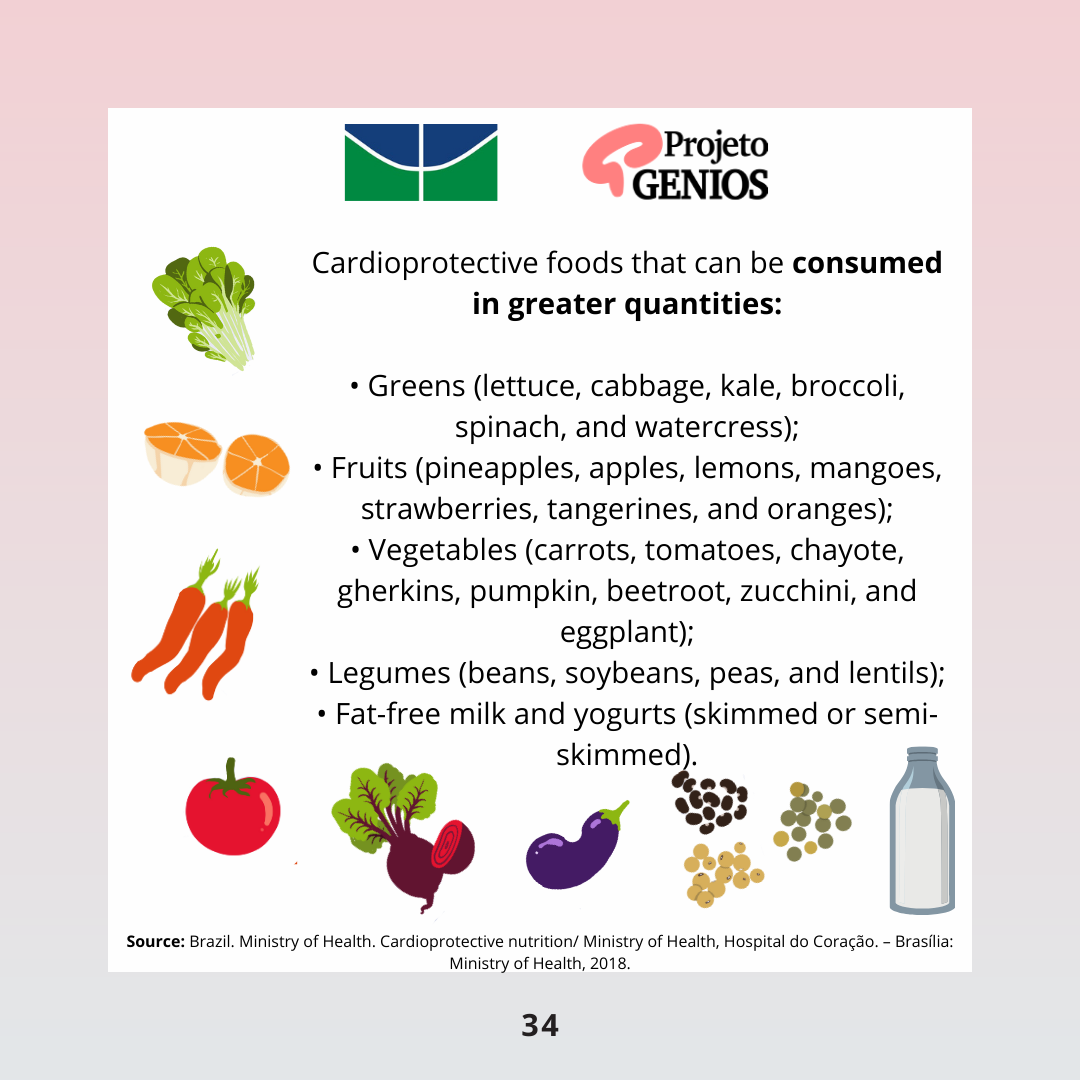

Supplement: Supplementary file 1 [file ijerph-22-00282-s001.zip › Instante Messages English/34.png]

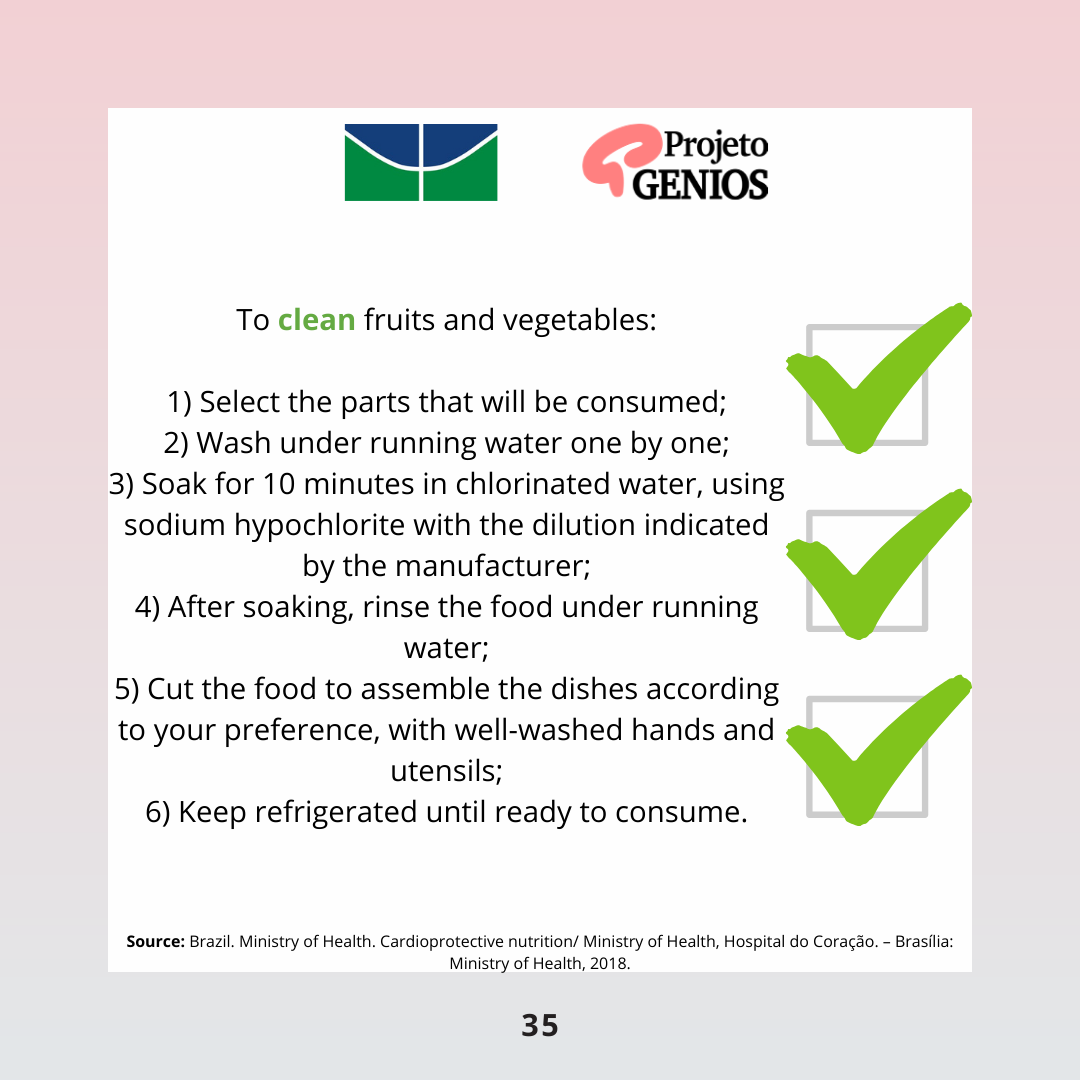

Supplement: Supplementary file 1 [file ijerph-22-00282-s001.zip › Instante Messages English/35.png]

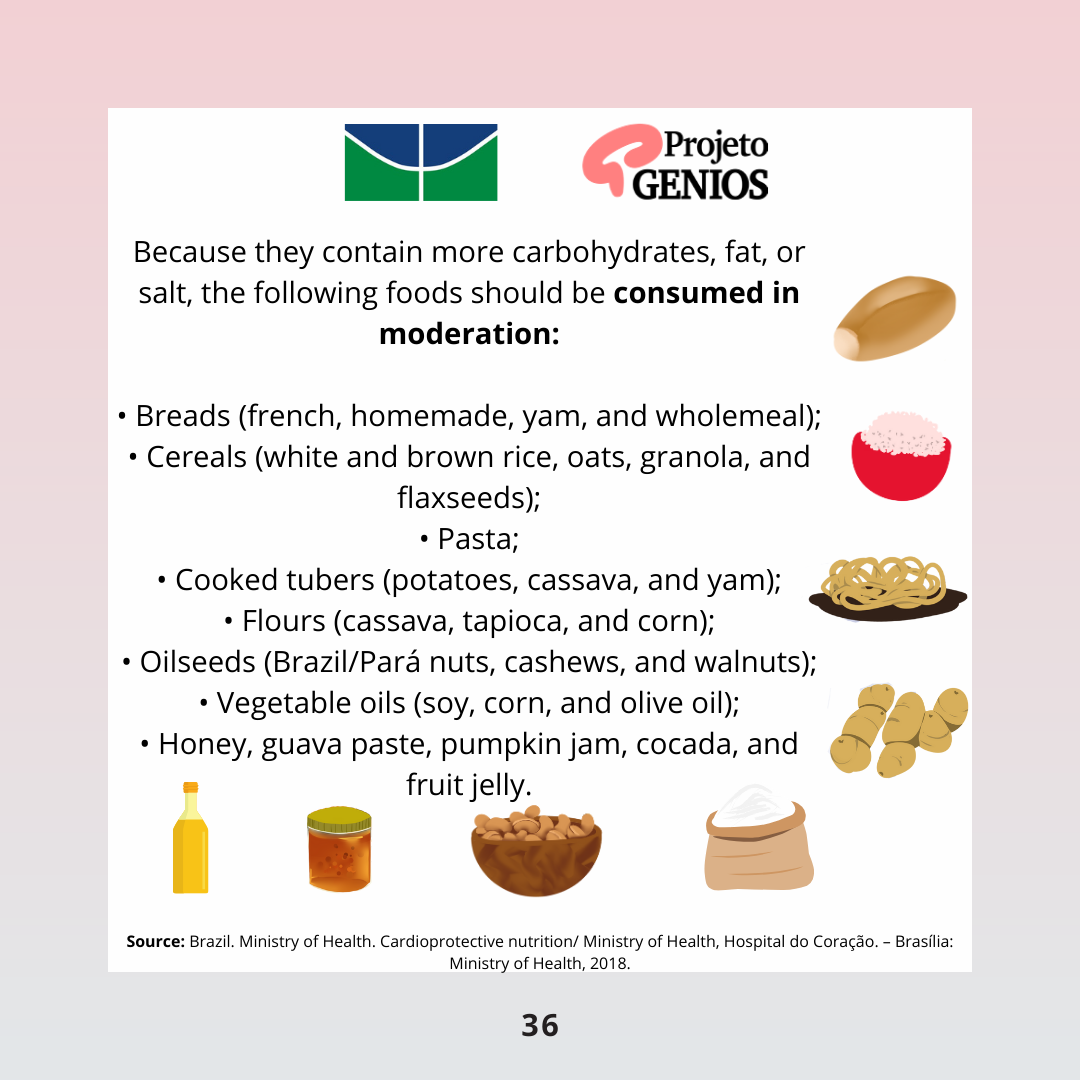

Supplement: Supplementary file 1 [file ijerph-22-00282-s001.zip › Instante Messages English/36.png]

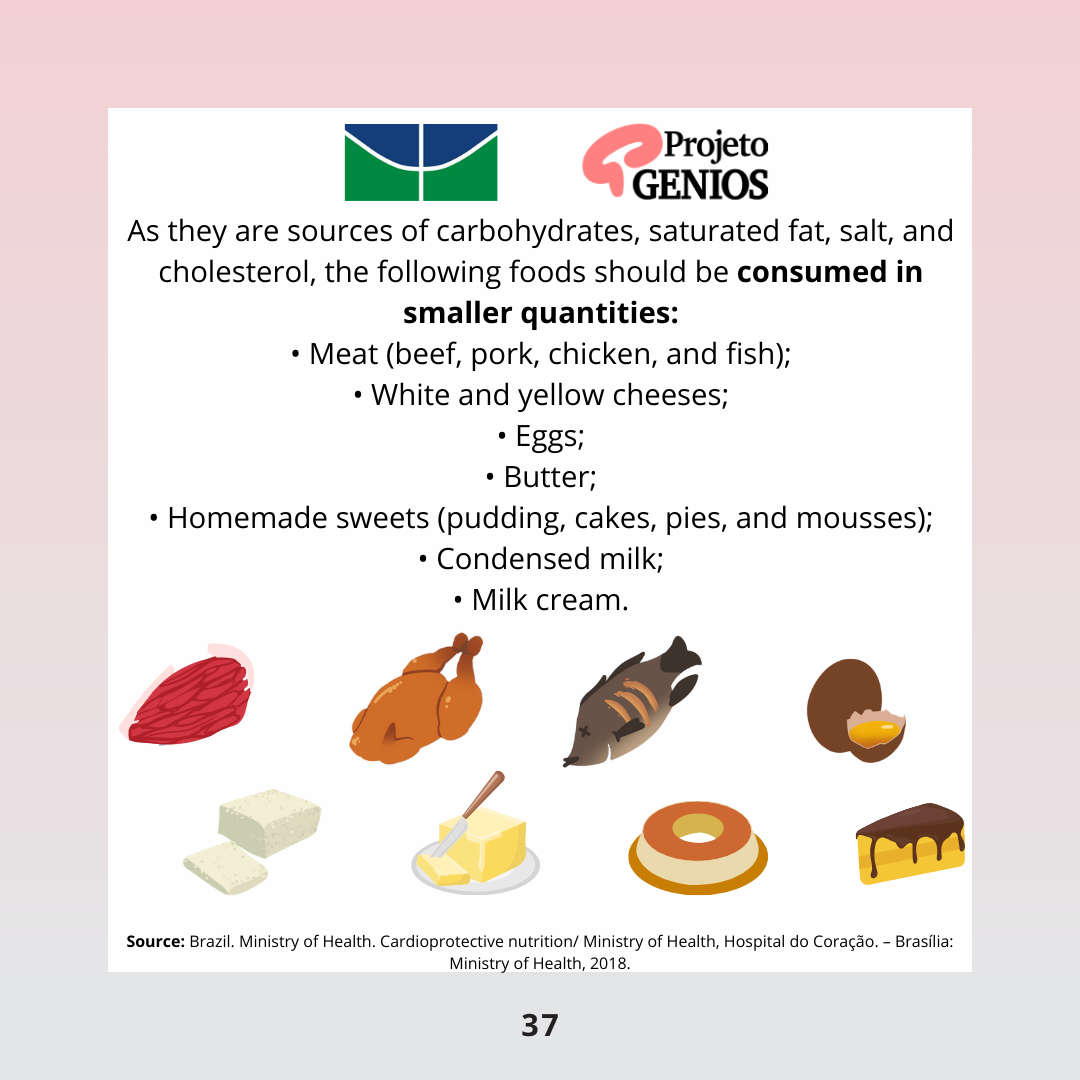

Supplement: Supplementary file 1 [file ijerph-22-00282-s001.zip › Instante Messages English/37.png]

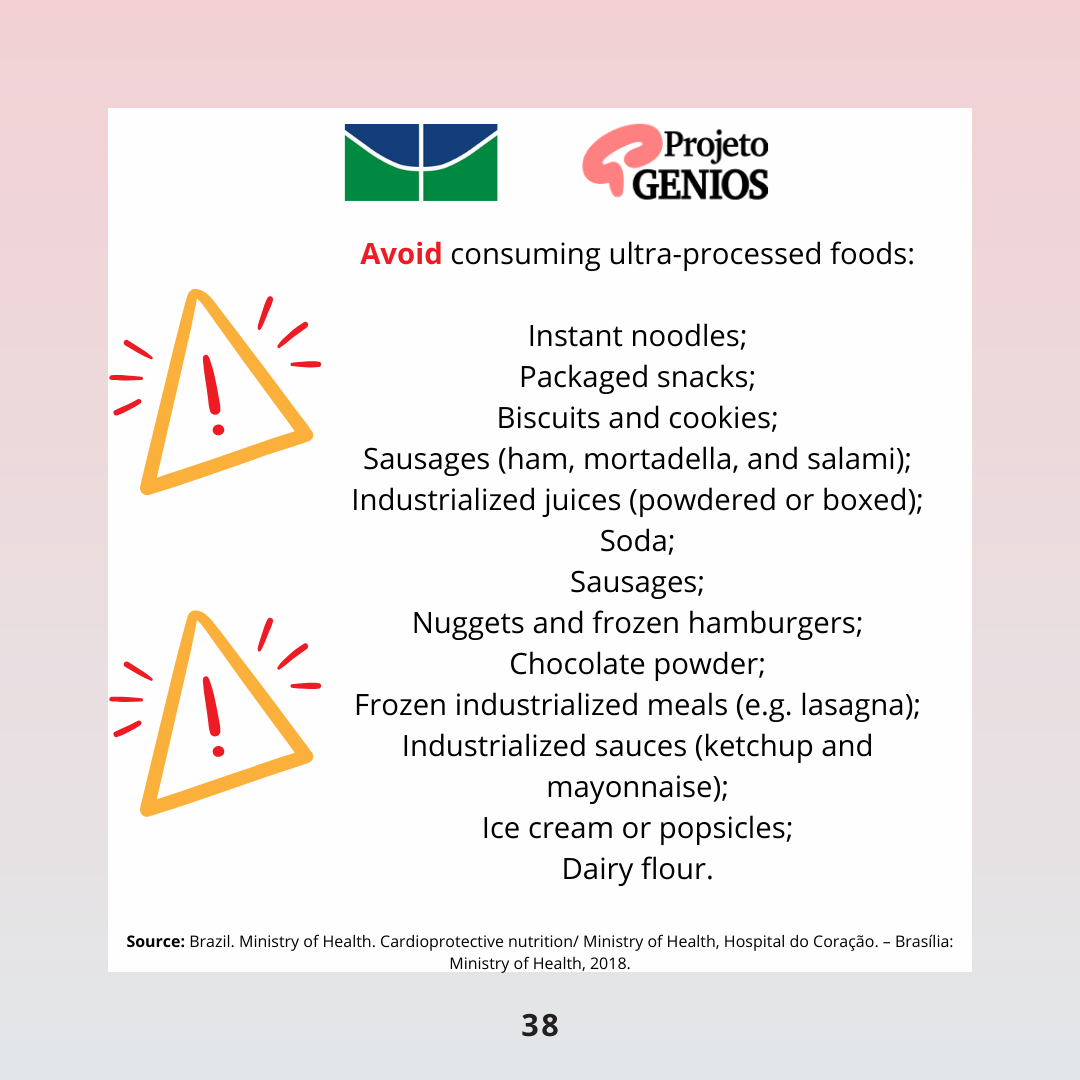

Supplement: Supplementary file 1 [file ijerph-22-00282-s001.zip › Instante Messages English/38.png]

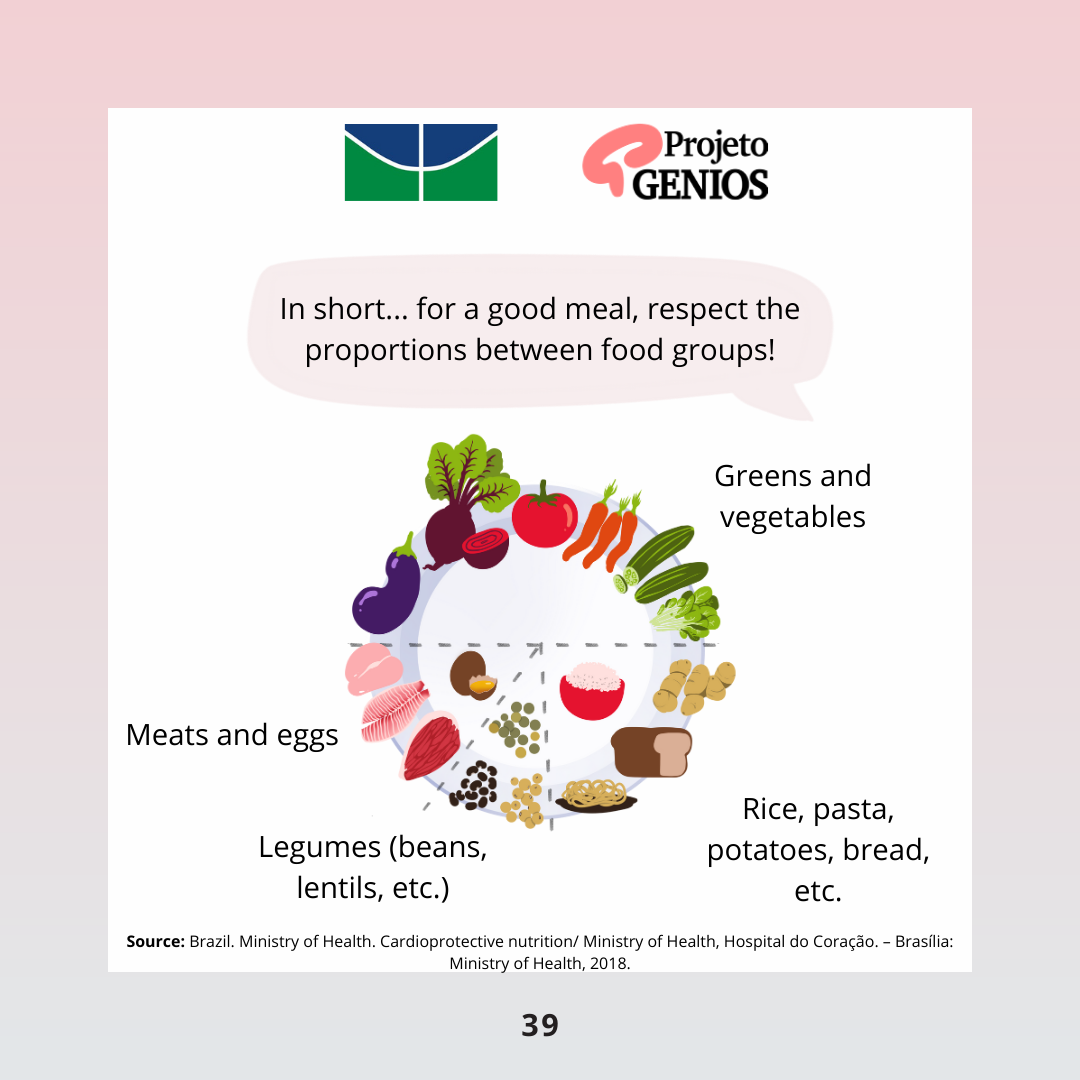

Supplement: Supplementary file 1 [file ijerph-22-00282-s001.zip › Instante Messages English/39.png]

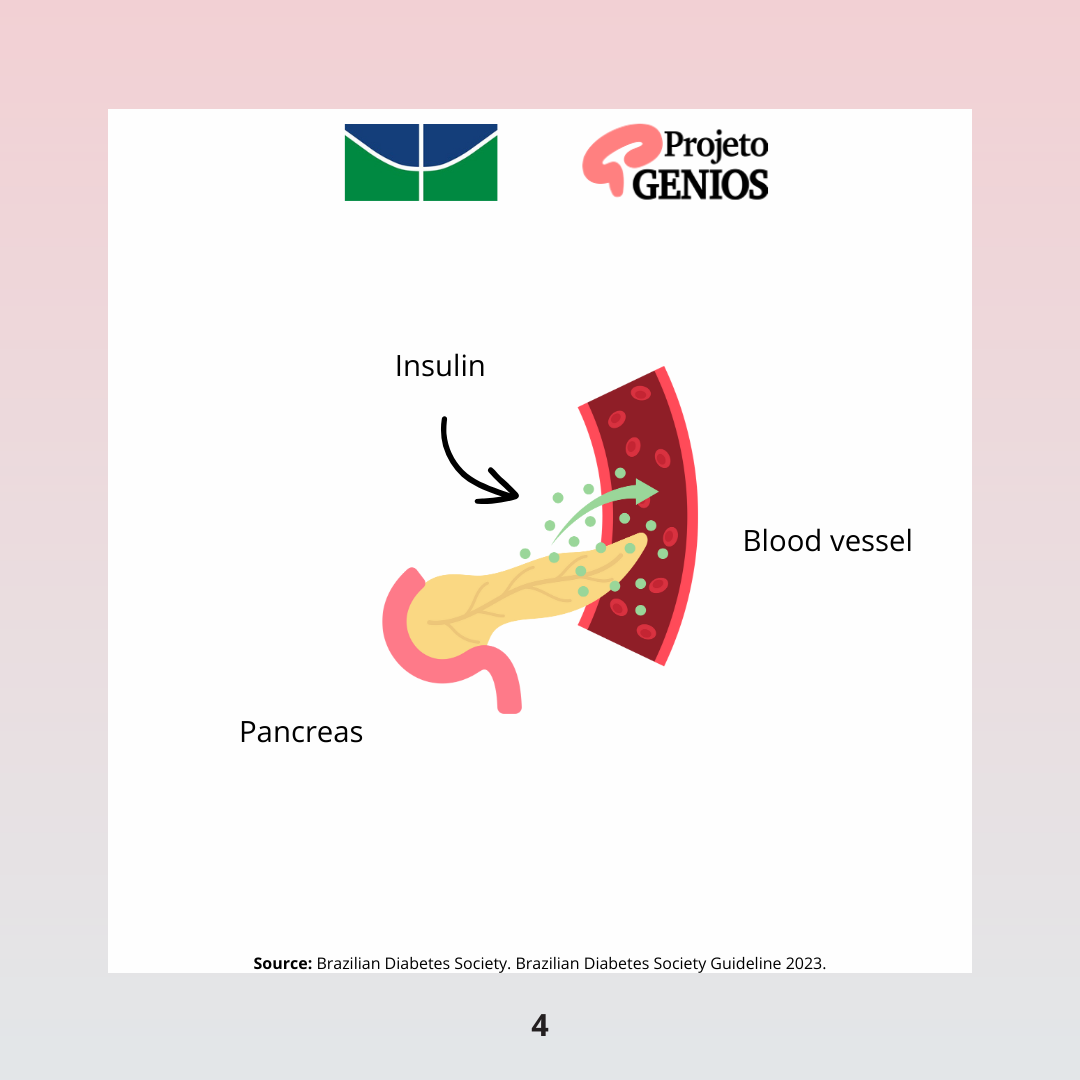

Supplement: Supplementary file 1 [file ijerph-22-00282-s001.zip › Instante Messages English/4.png]

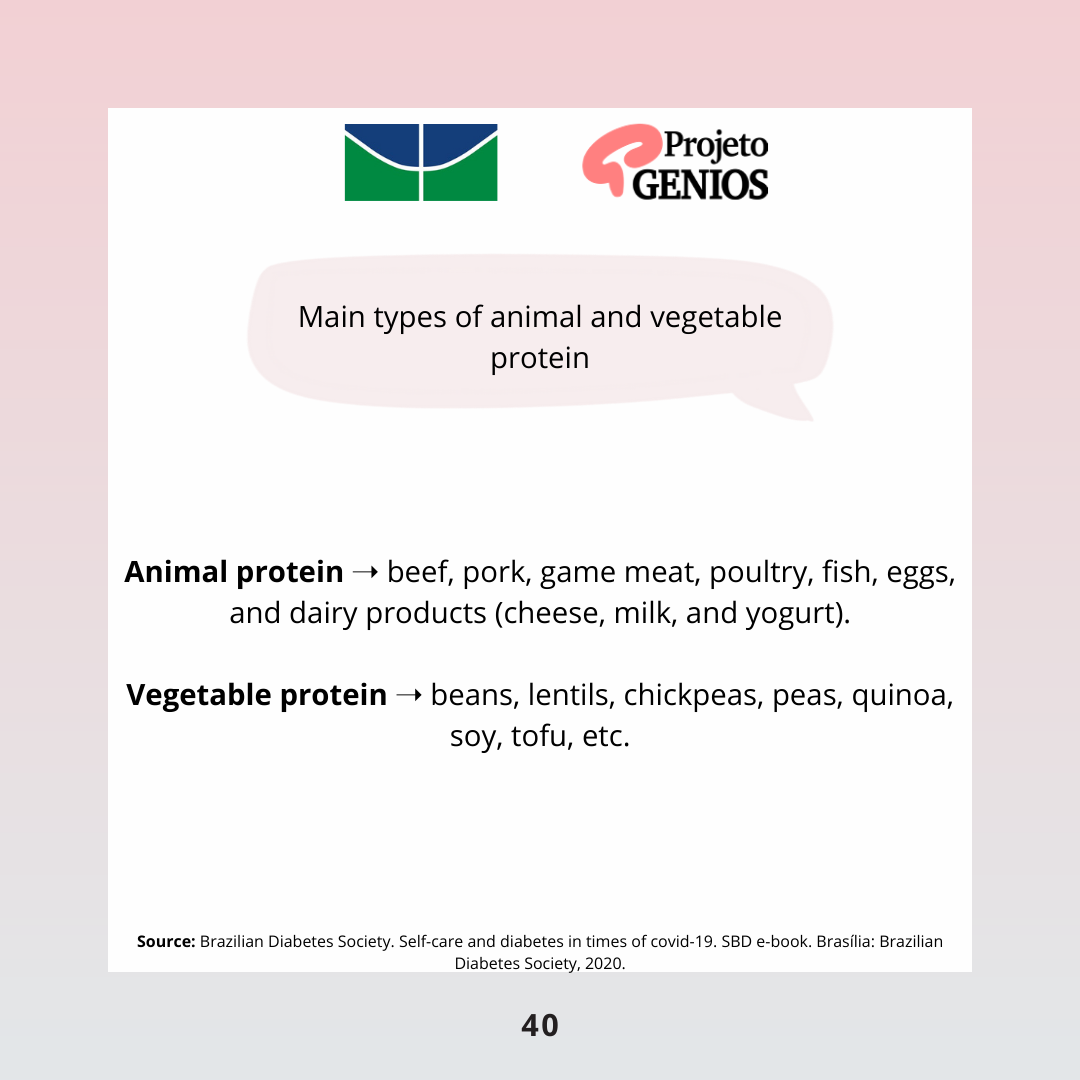

Supplement: Supplementary file 1 [file ijerph-22-00282-s001.zip › Instante Messages English/40.png]

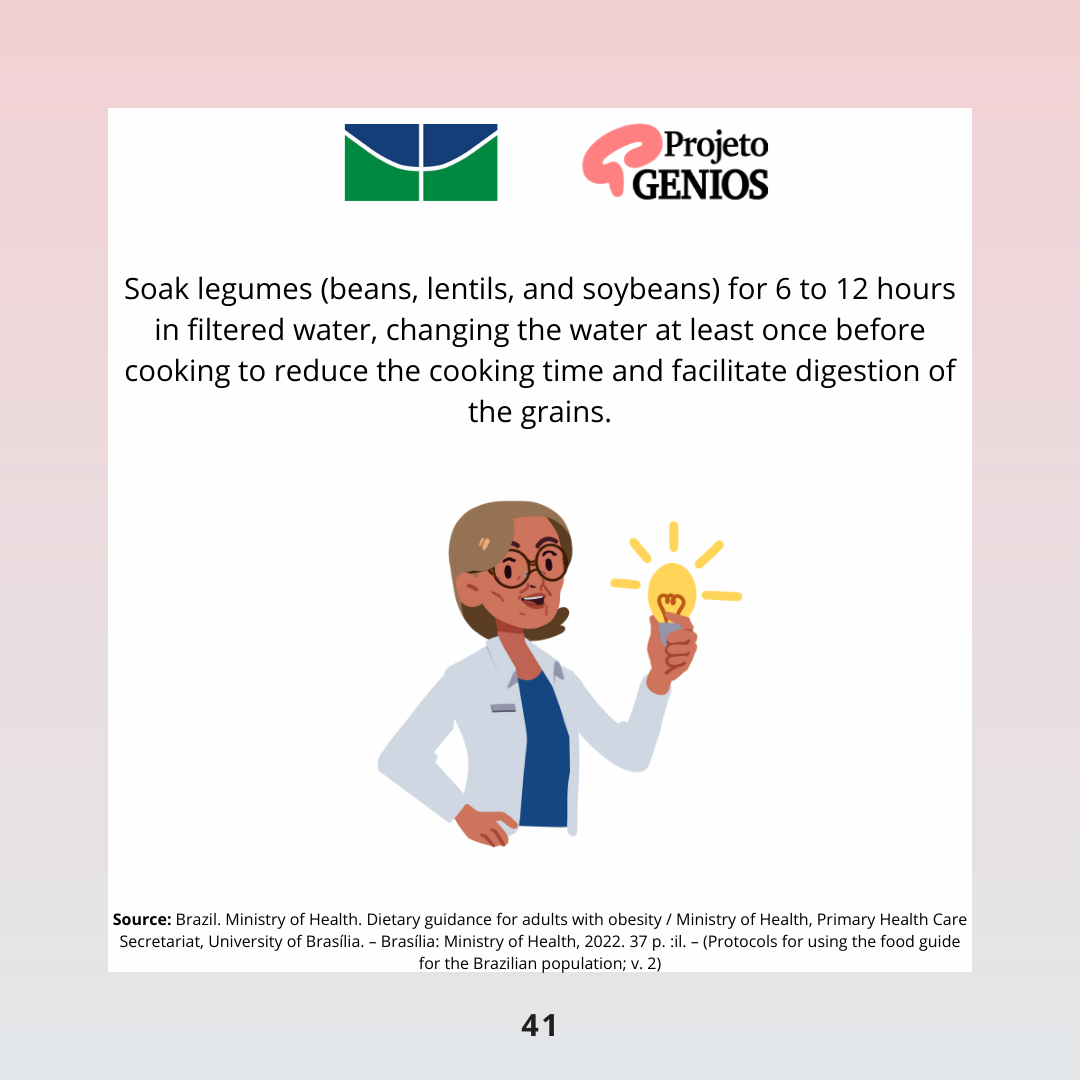

Supplement: Supplementary file 1 [file ijerph-22-00282-s001.zip › Instante Messages English/41.png]

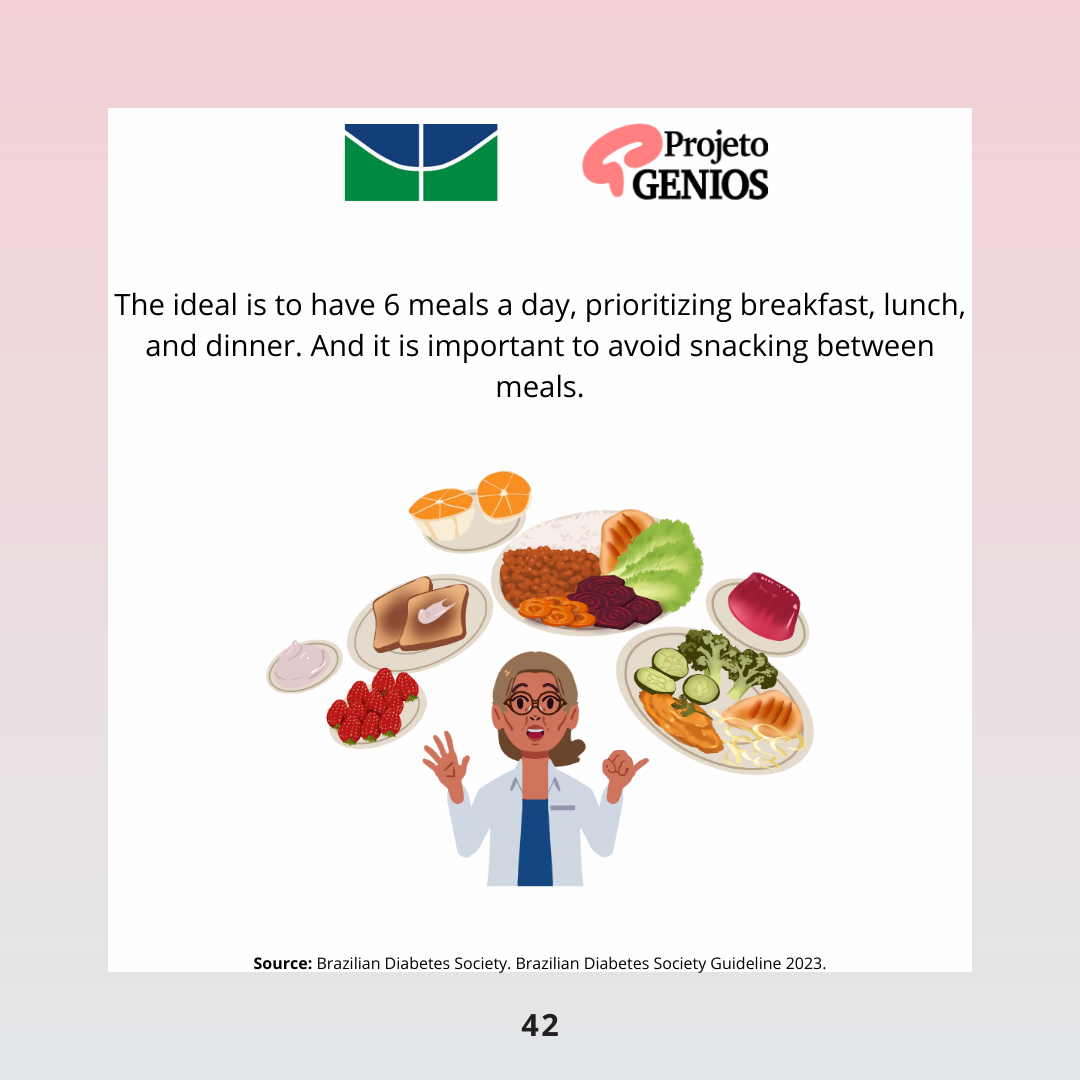

Supplement: Supplementary file 1 [file ijerph-22-00282-s001.zip › Instante Messages English/42.png]

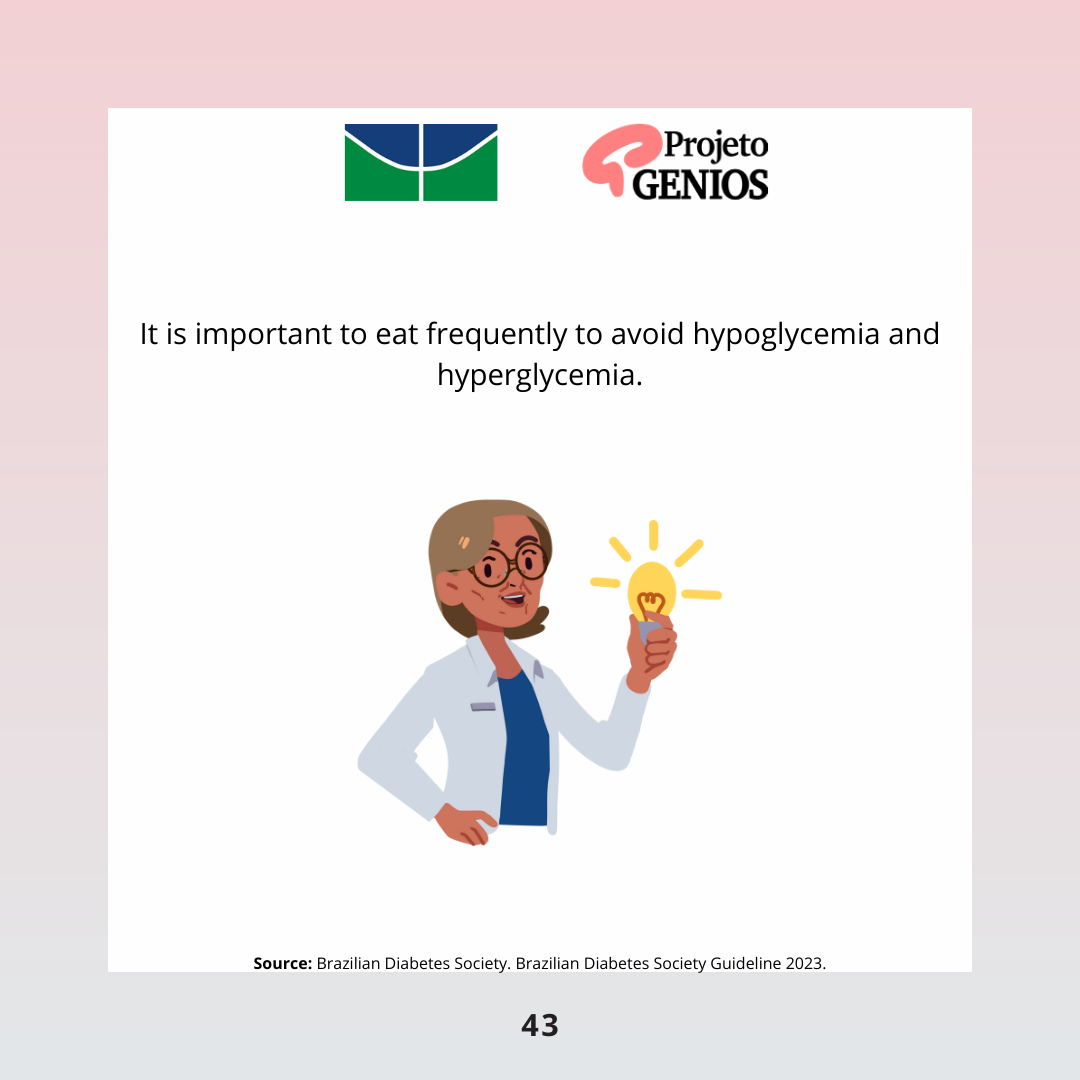

Supplement: Supplementary file 1 [file ijerph-22-00282-s001.zip › Instante Messages English/43.png]

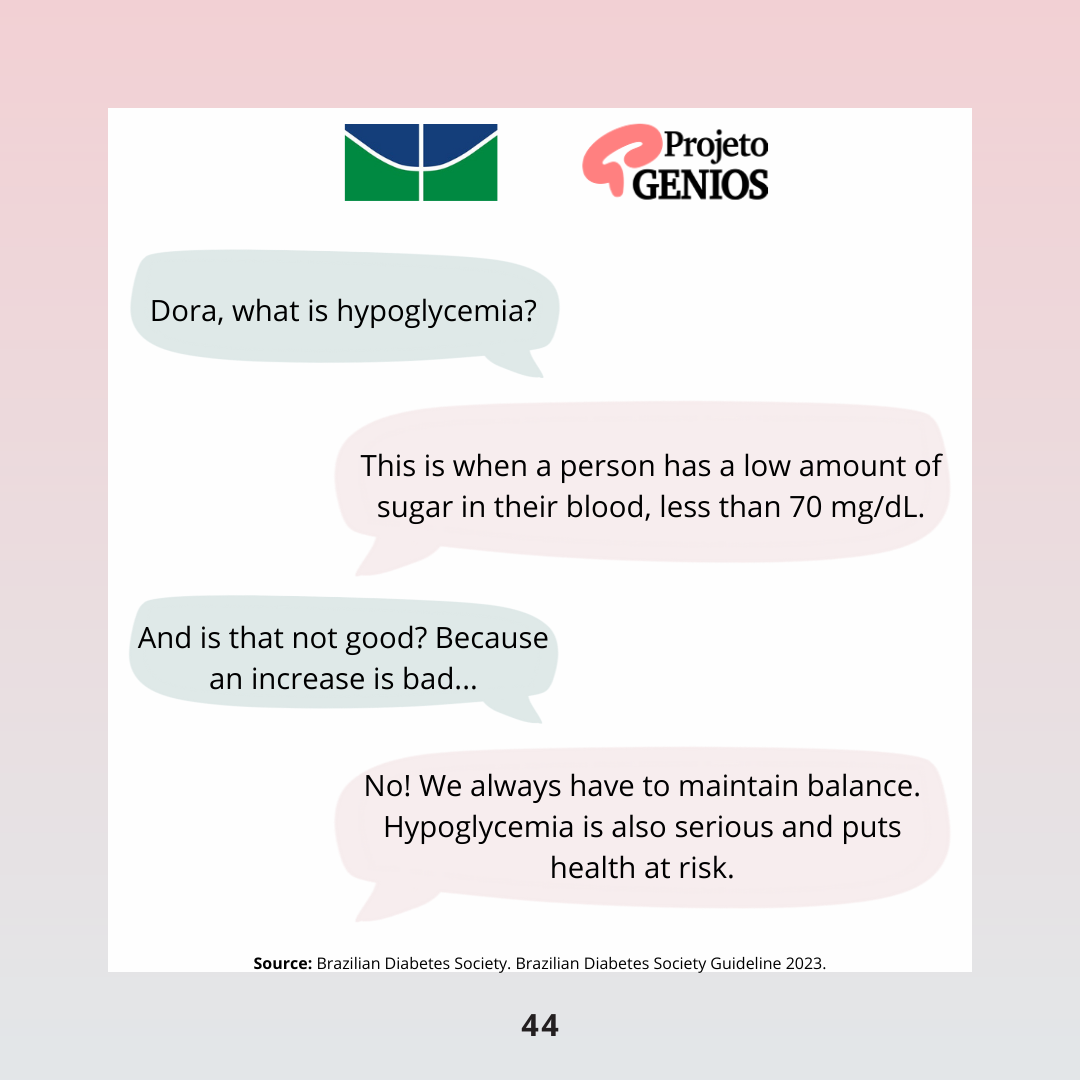

Supplement: Supplementary file 1 [file ijerph-22-00282-s001.zip › Instante Messages English/44.png]

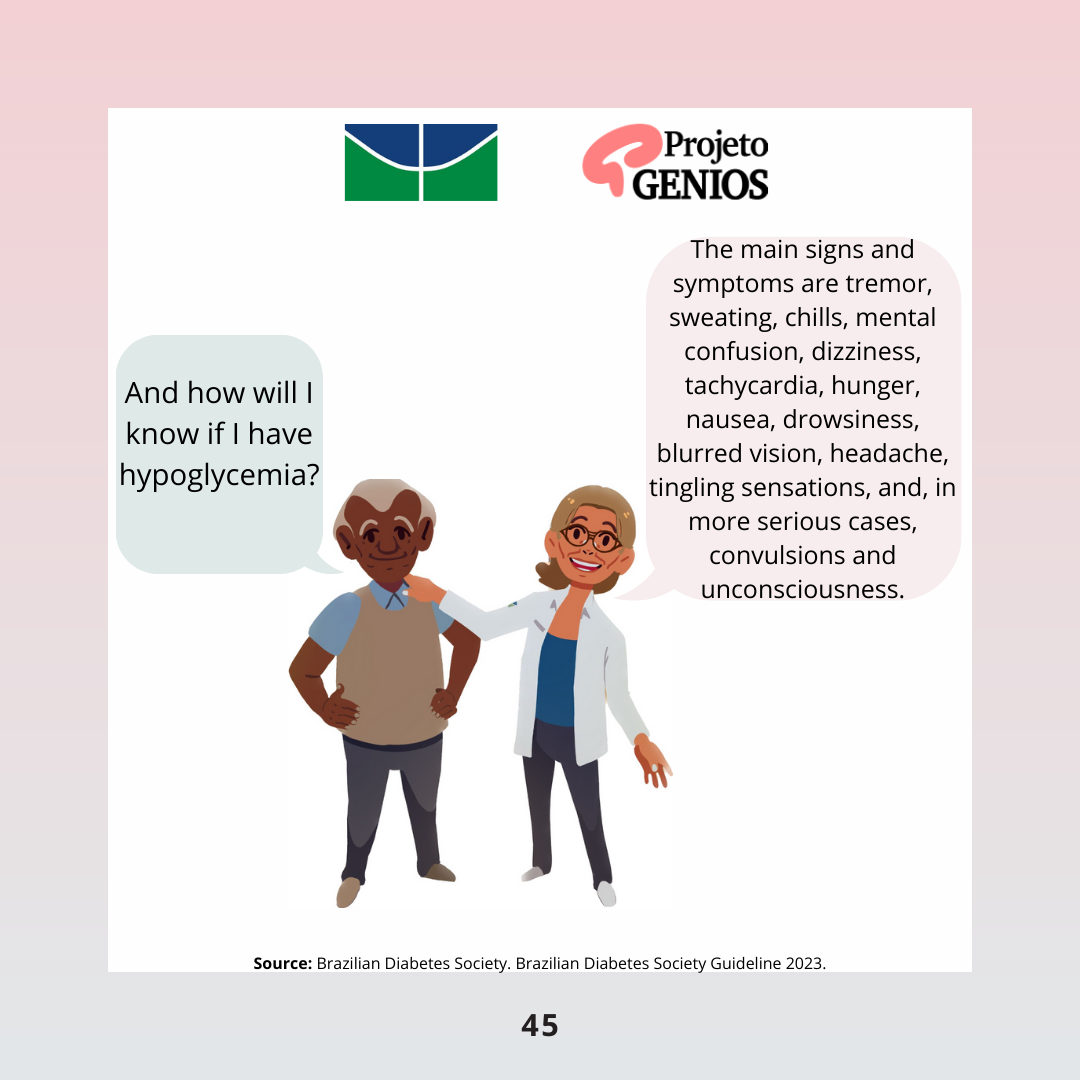

Supplement: Supplementary file 1 [file ijerph-22-00282-s001.zip › Instante Messages English/45.png]

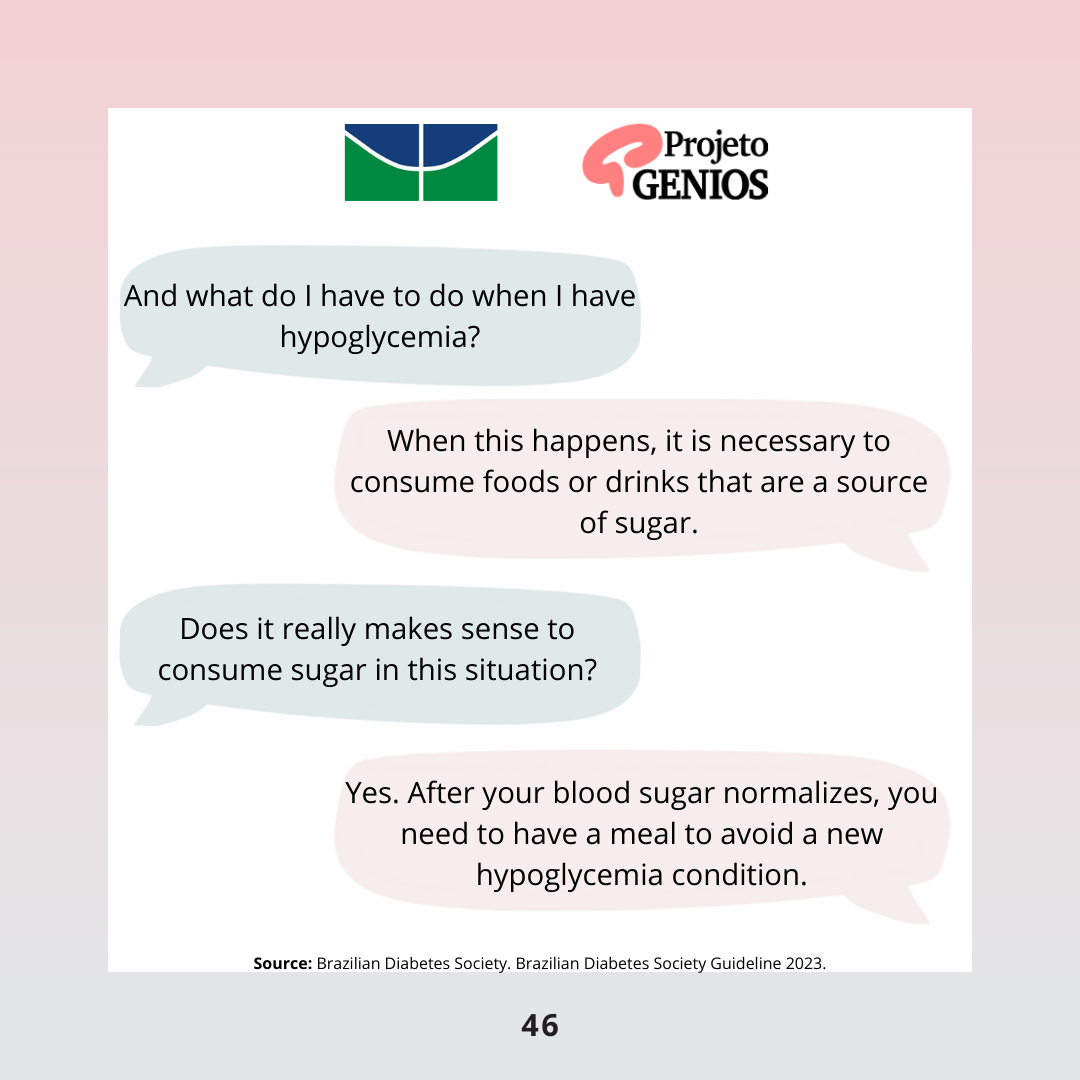

Supplement: Supplementary file 1 [file ijerph-22-00282-s001.zip › Instante Messages English/46.png]

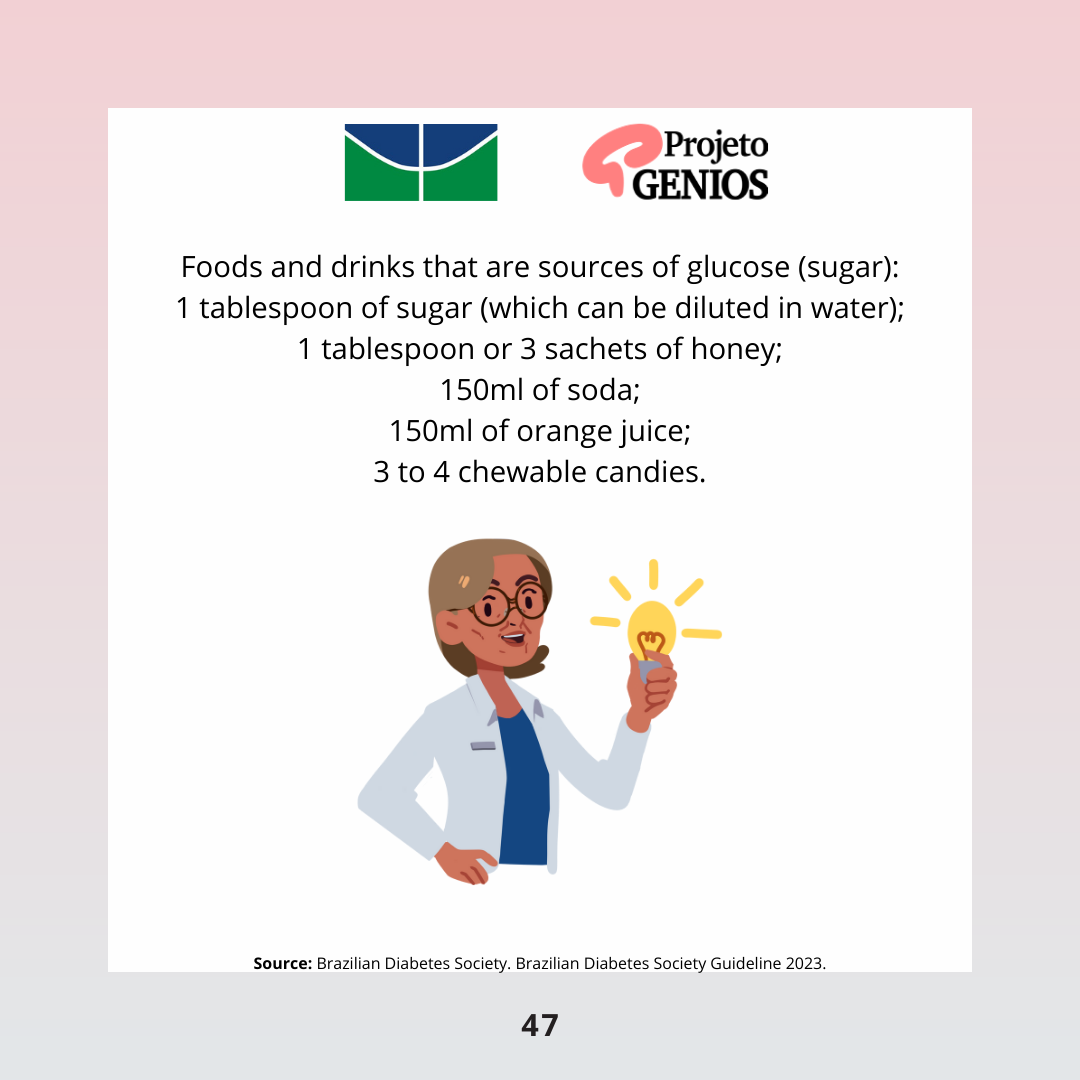

Supplement: Supplementary file 1 [file ijerph-22-00282-s001.zip › Instante Messages English/47.png]

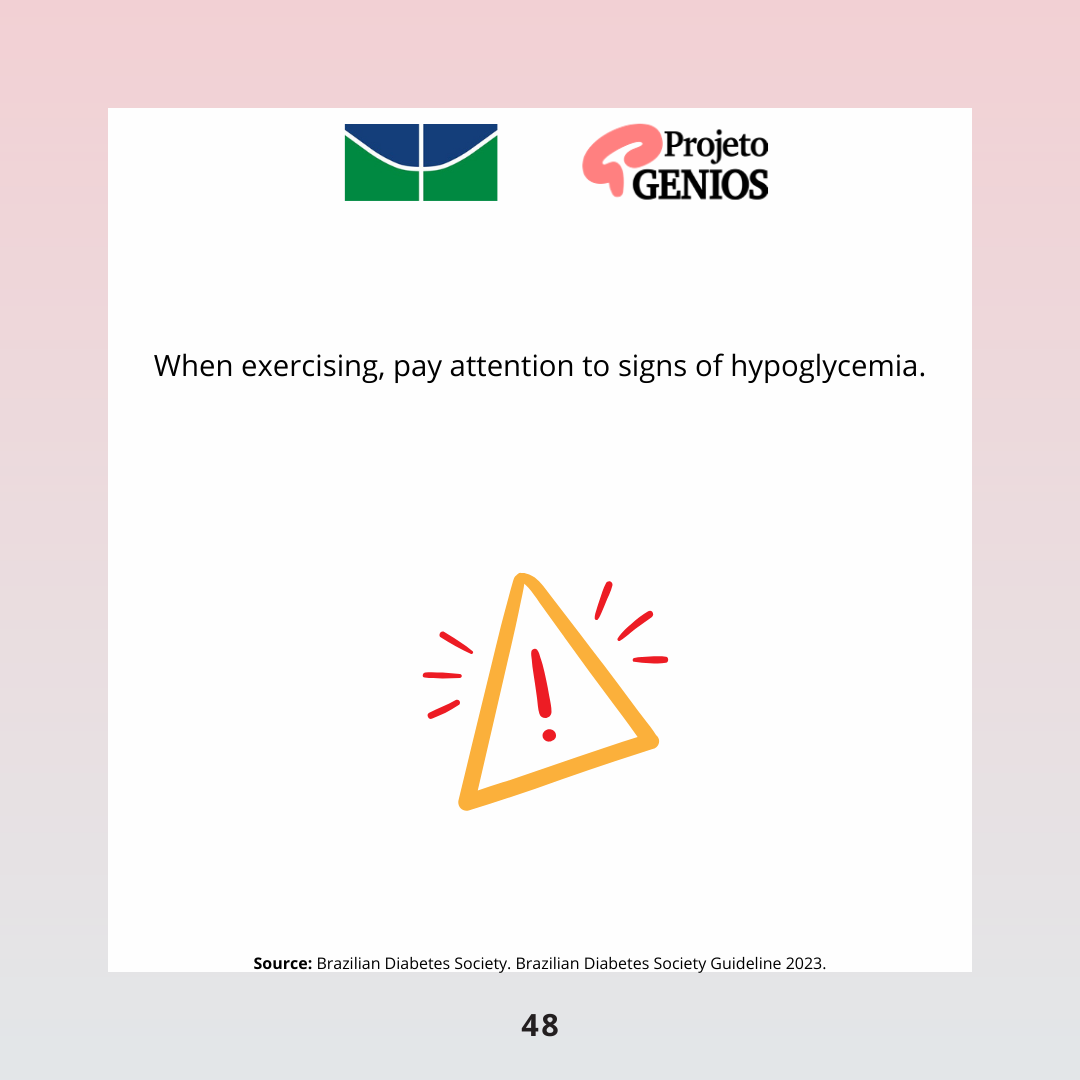

Supplement: Supplementary file 1 [file ijerph-22-00282-s001.zip › Instante Messages English/48.png]

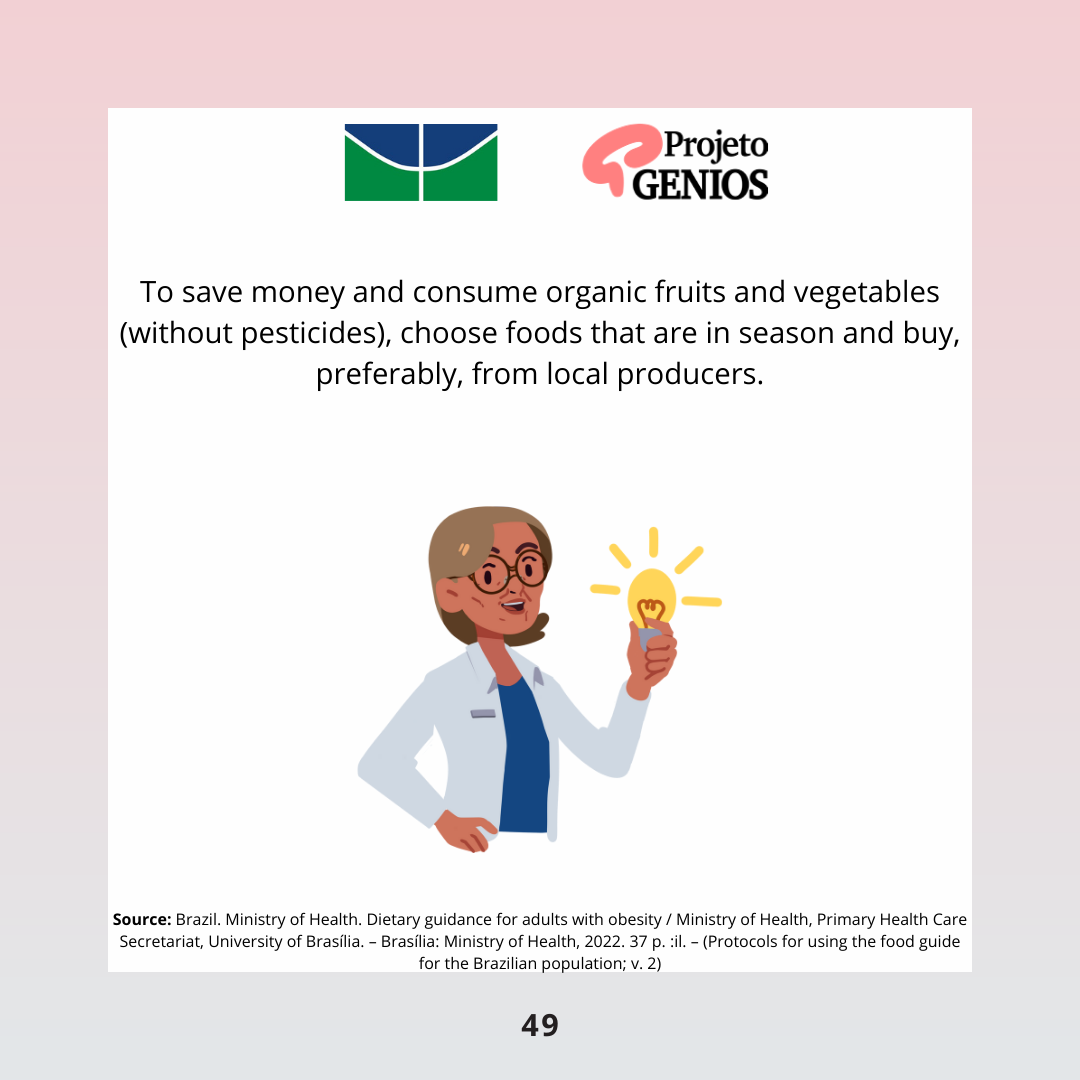

Supplement: Supplementary file 1 [file ijerph-22-00282-s001.zip › Instante Messages English/49.png]

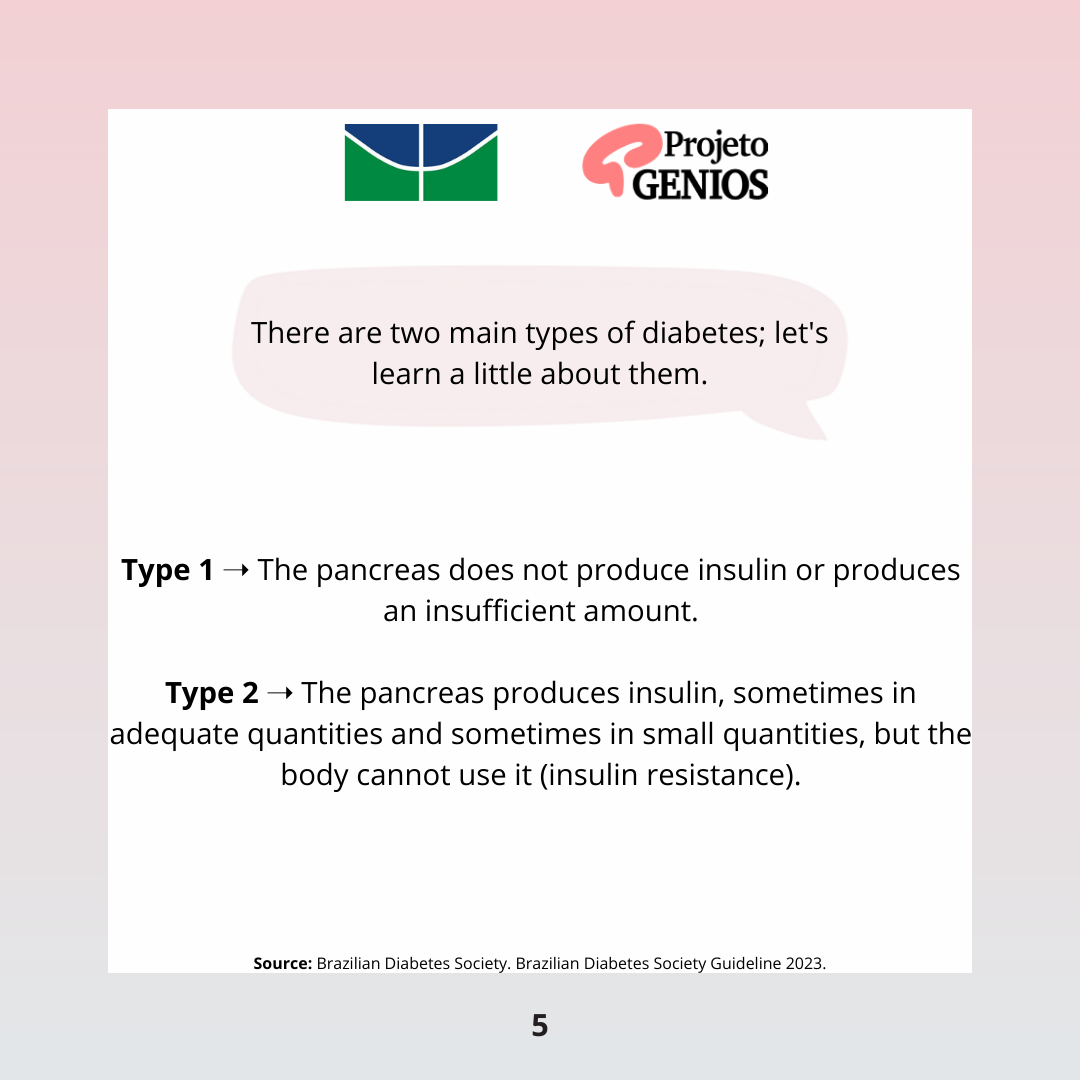

Supplement: Supplementary file 1 [file ijerph-22-00282-s001.zip › Instante Messages English/5.png]

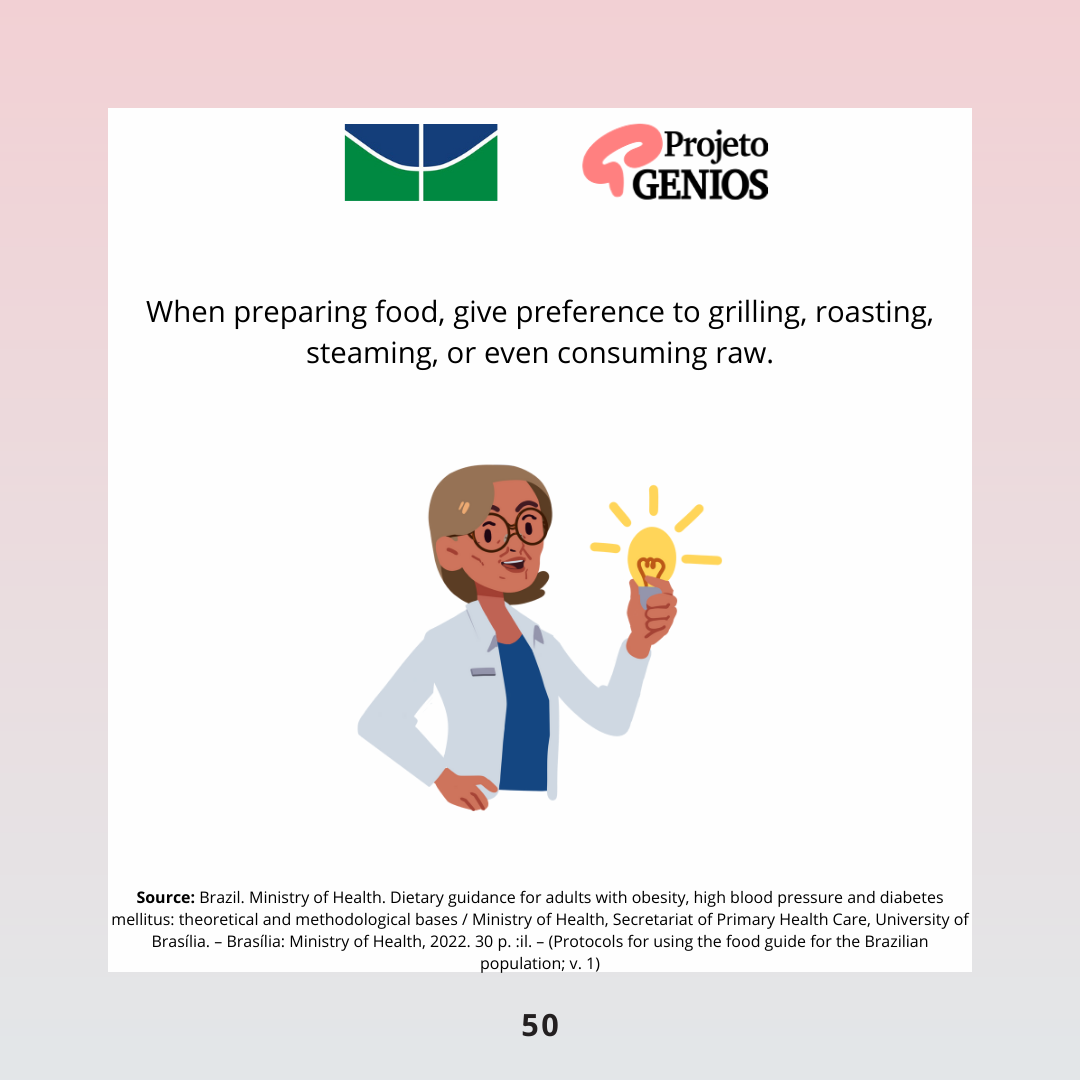

Supplement: Supplementary file 1 [file ijerph-22-00282-s001.zip › Instante Messages English/50.png]

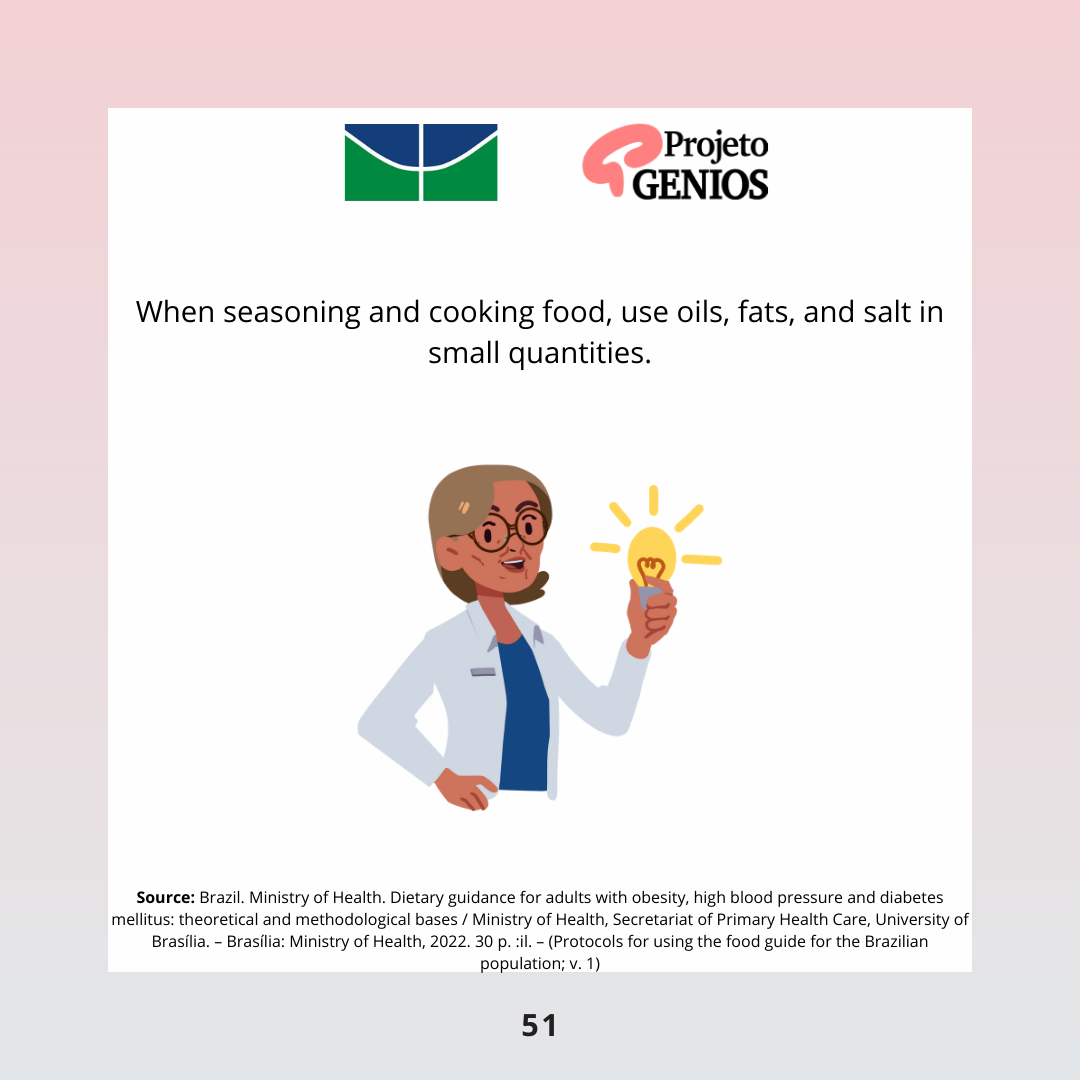

Supplement: Supplementary file 1 [file ijerph-22-00282-s001.zip › Instante Messages English/51.png]

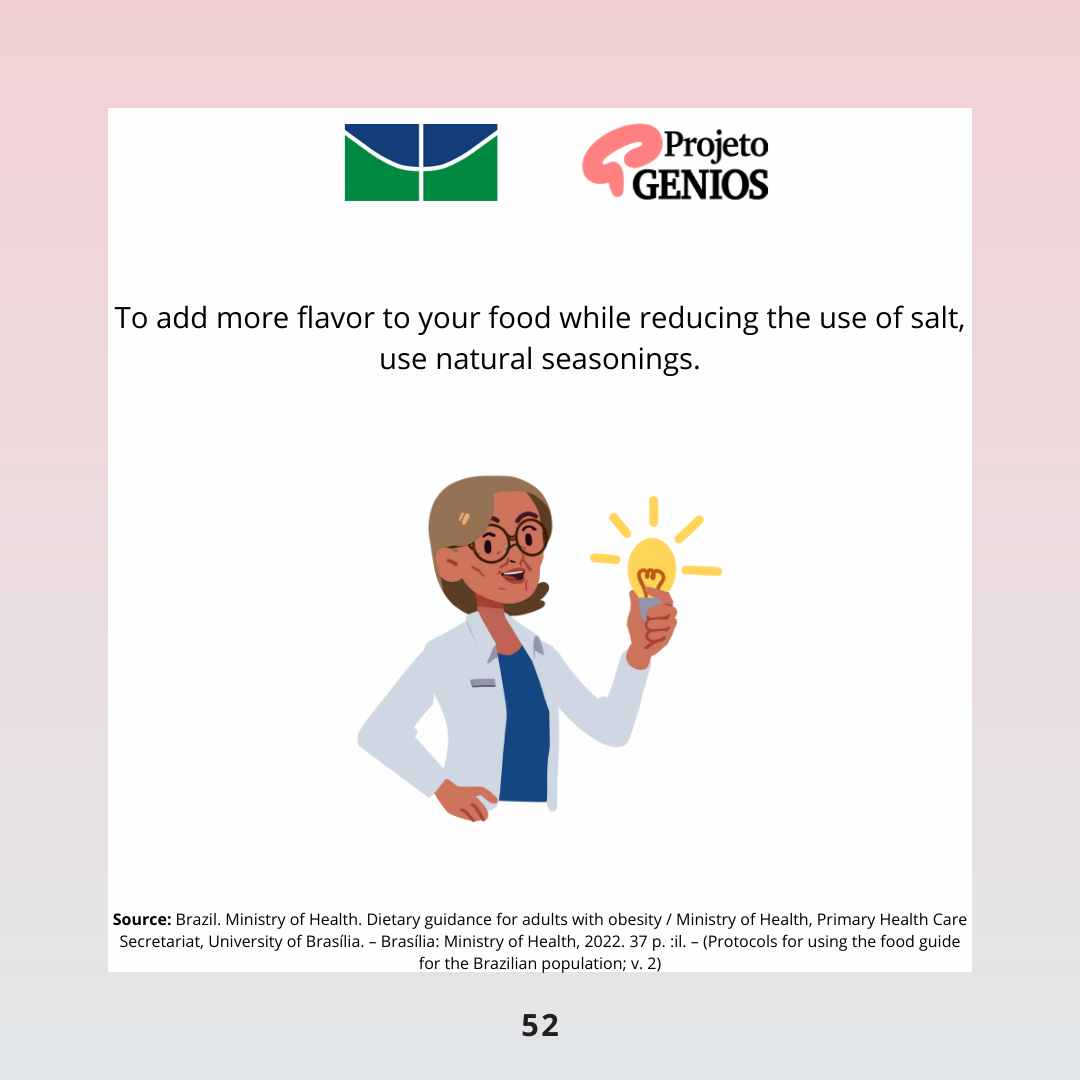

Supplement: Supplementary file 1 [file ijerph-22-00282-s001.zip › Instante Messages English/52.png]

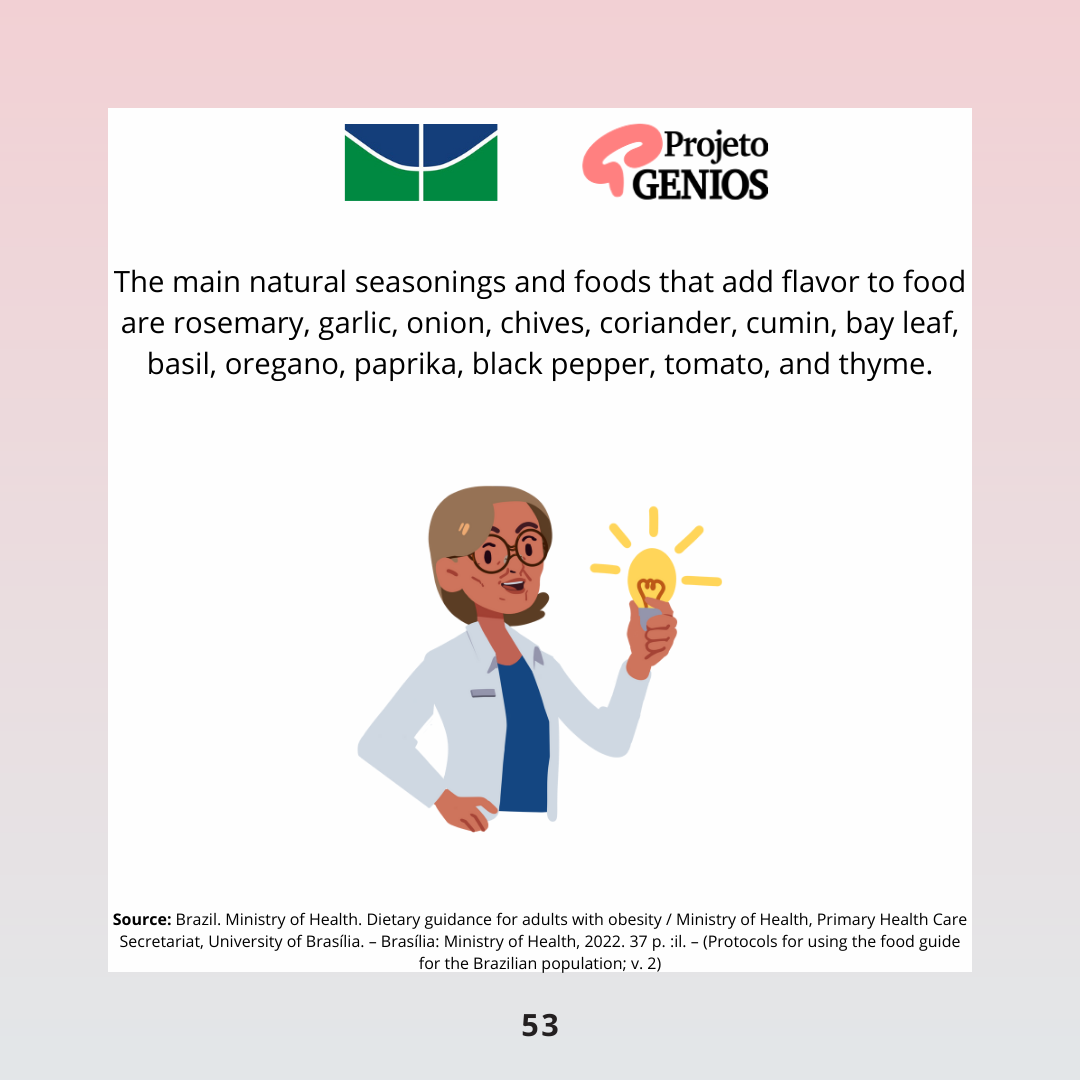

Supplement: Supplementary file 1 [file ijerph-22-00282-s001.zip › Instante Messages English/53.png]

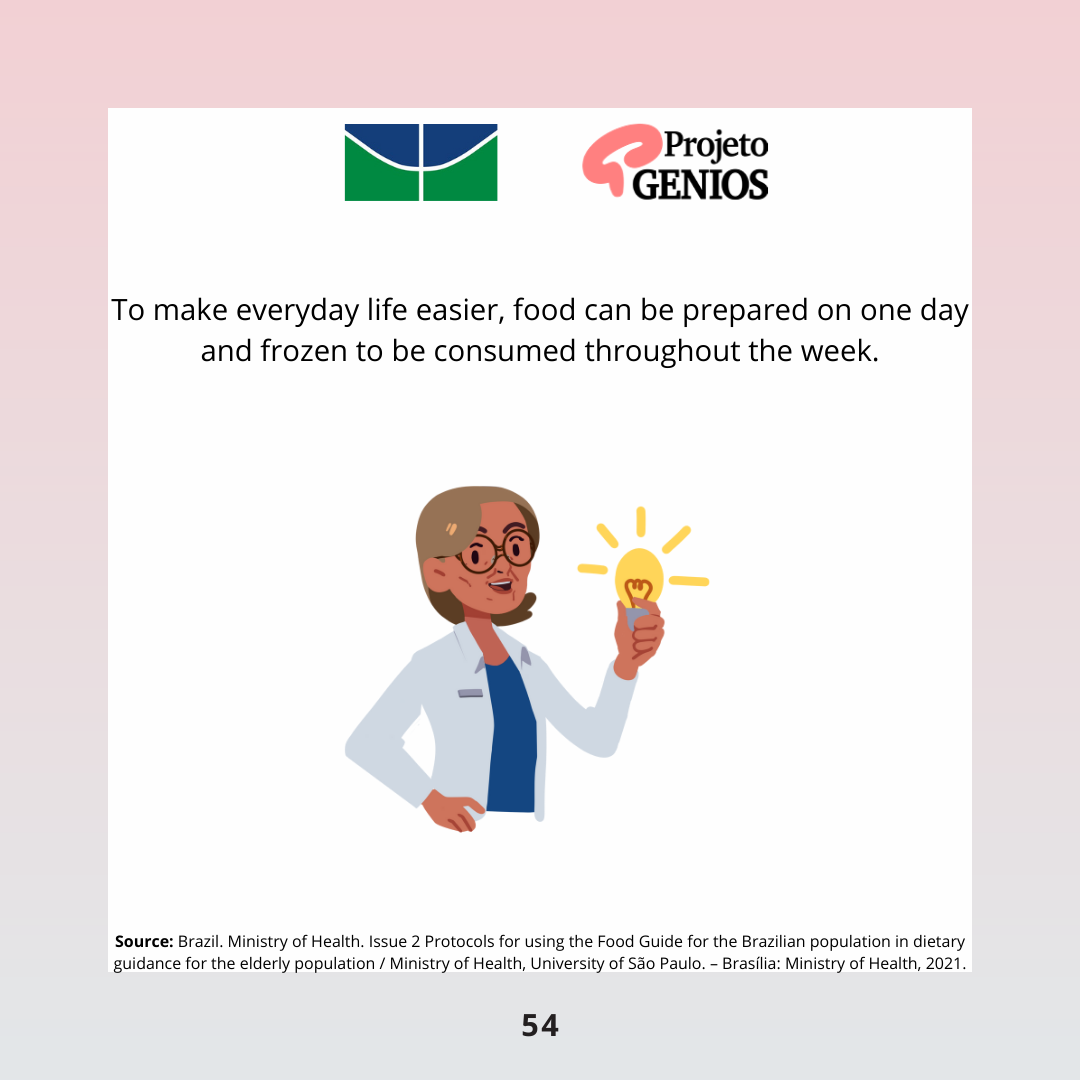

Supplement: Supplementary file 1 [file ijerph-22-00282-s001.zip › Instante Messages English/54.png]

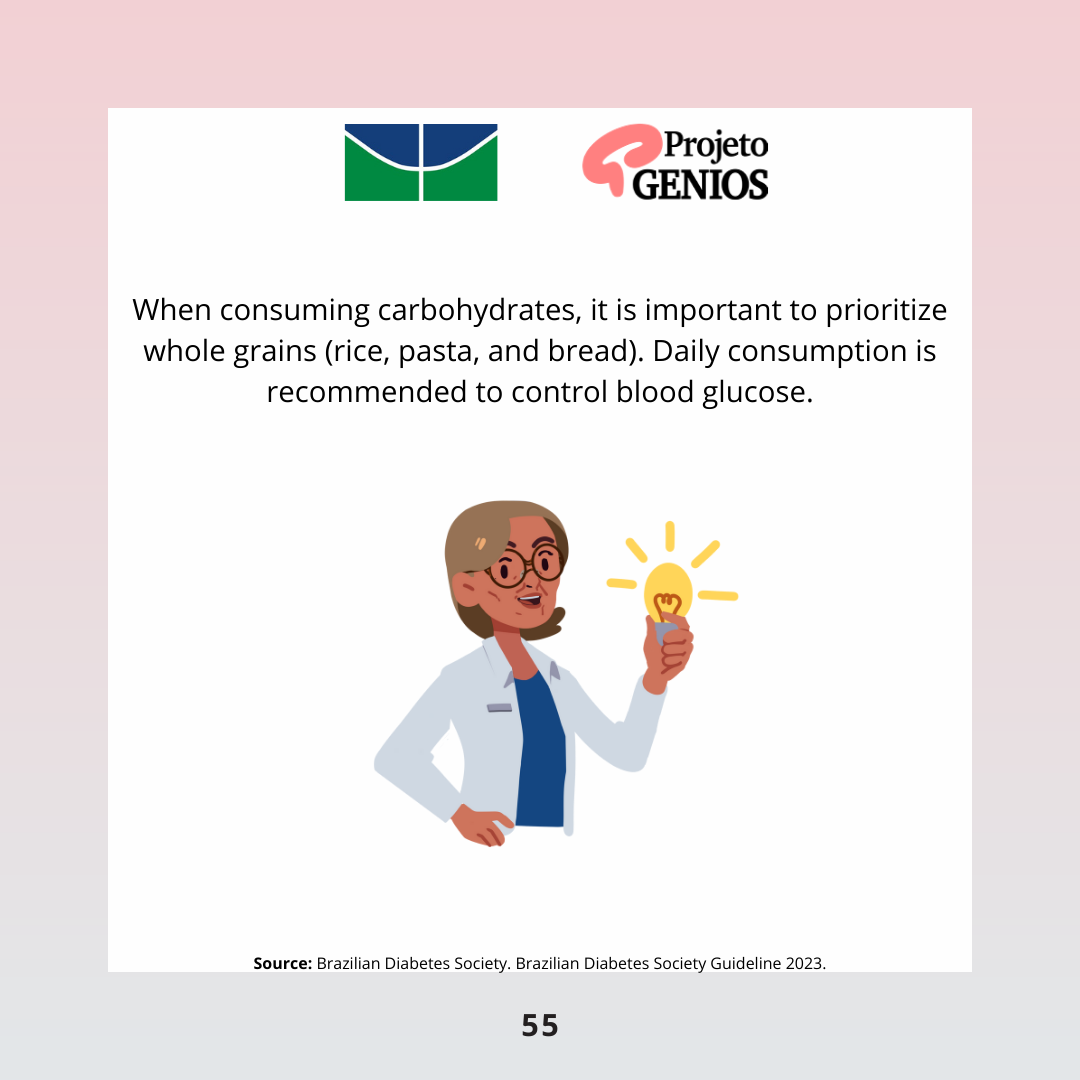

Supplement: Supplementary file 1 [file ijerph-22-00282-s001.zip › Instante Messages English/55.png]

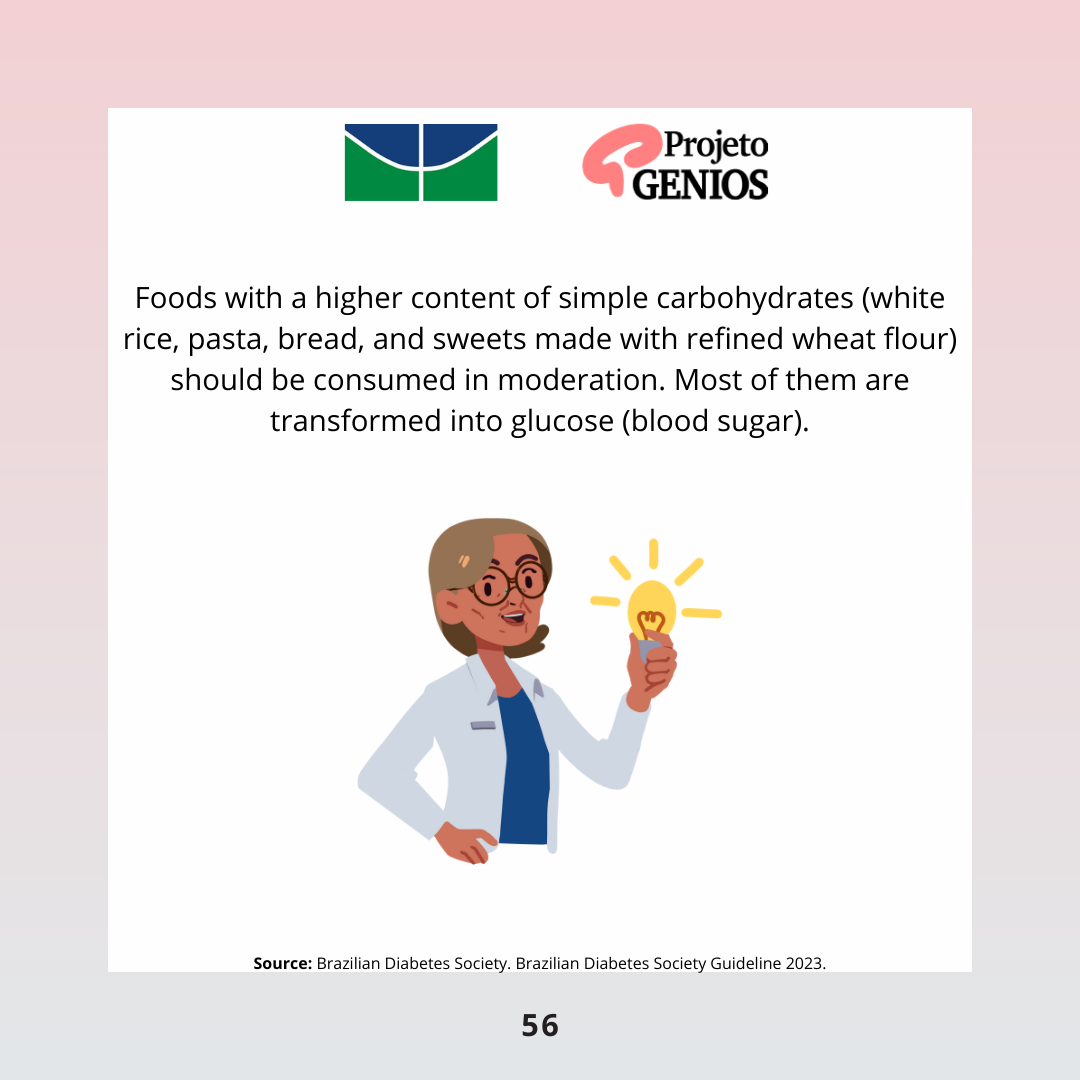

Supplement: Supplementary file 1 [file ijerph-22-00282-s001.zip › Instante Messages English/56.png]

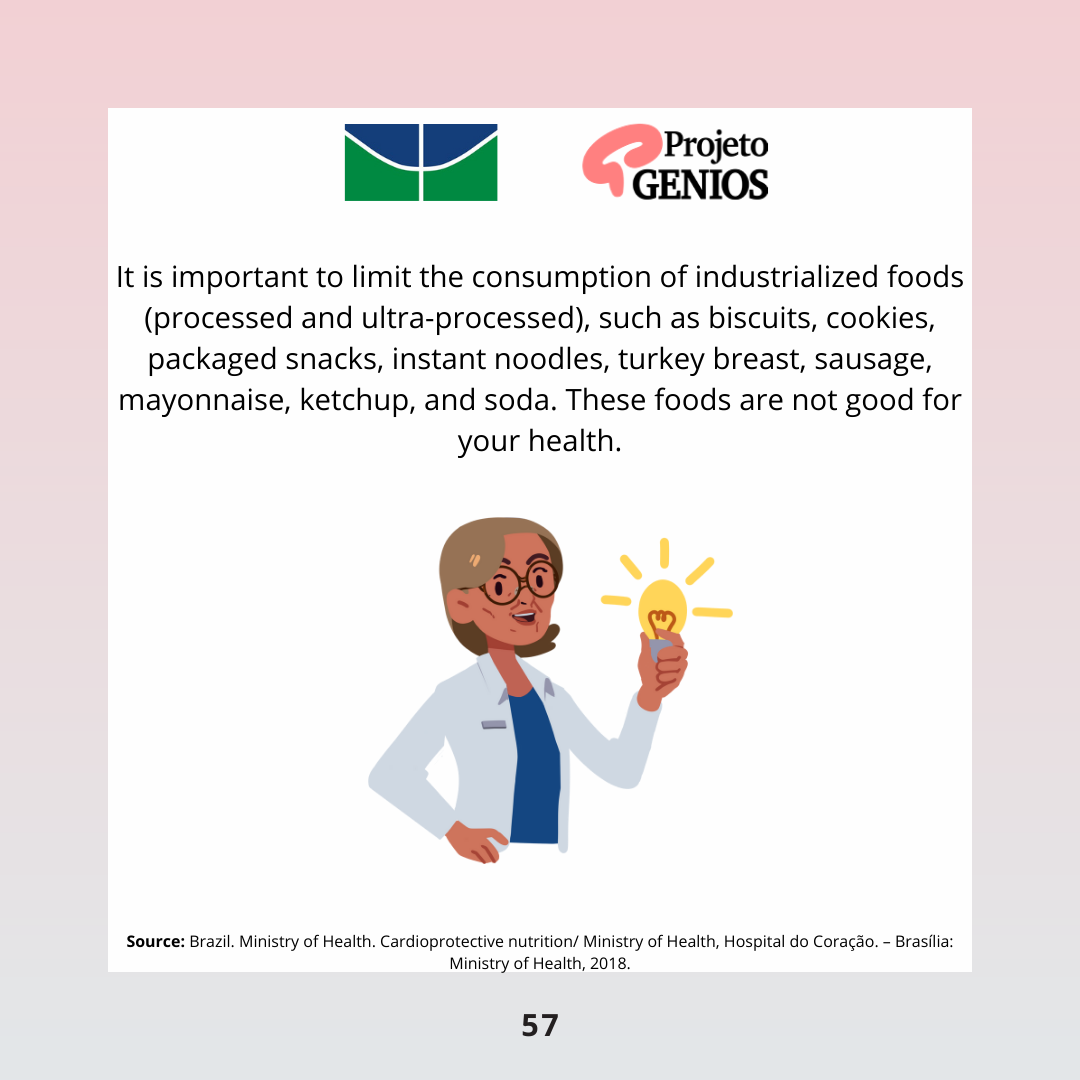

Supplement: Supplementary file 1 [file ijerph-22-00282-s001.zip › Instante Messages English/57.png]

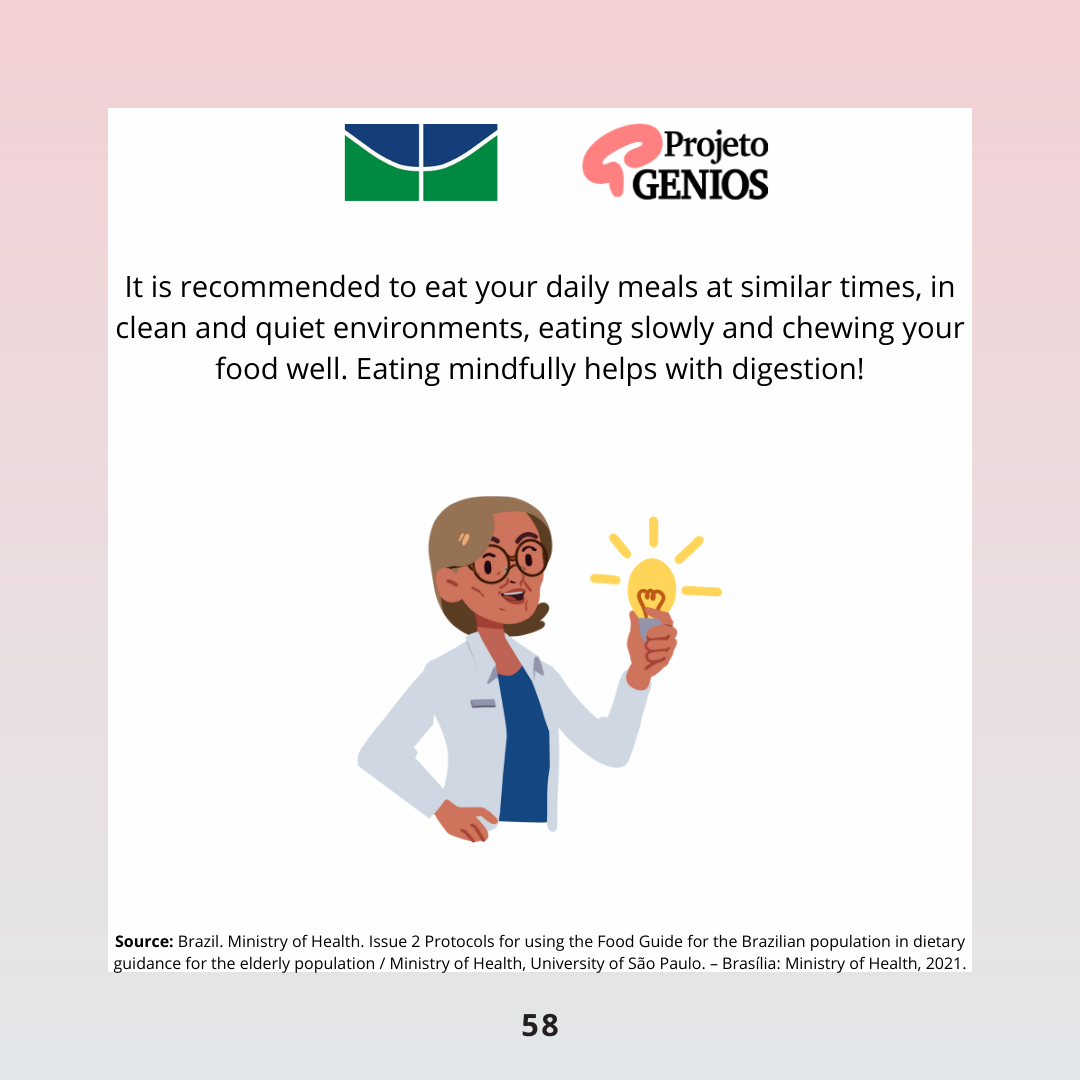

Supplement: Supplementary file 1 [file ijerph-22-00282-s001.zip › Instante Messages English/58.png]

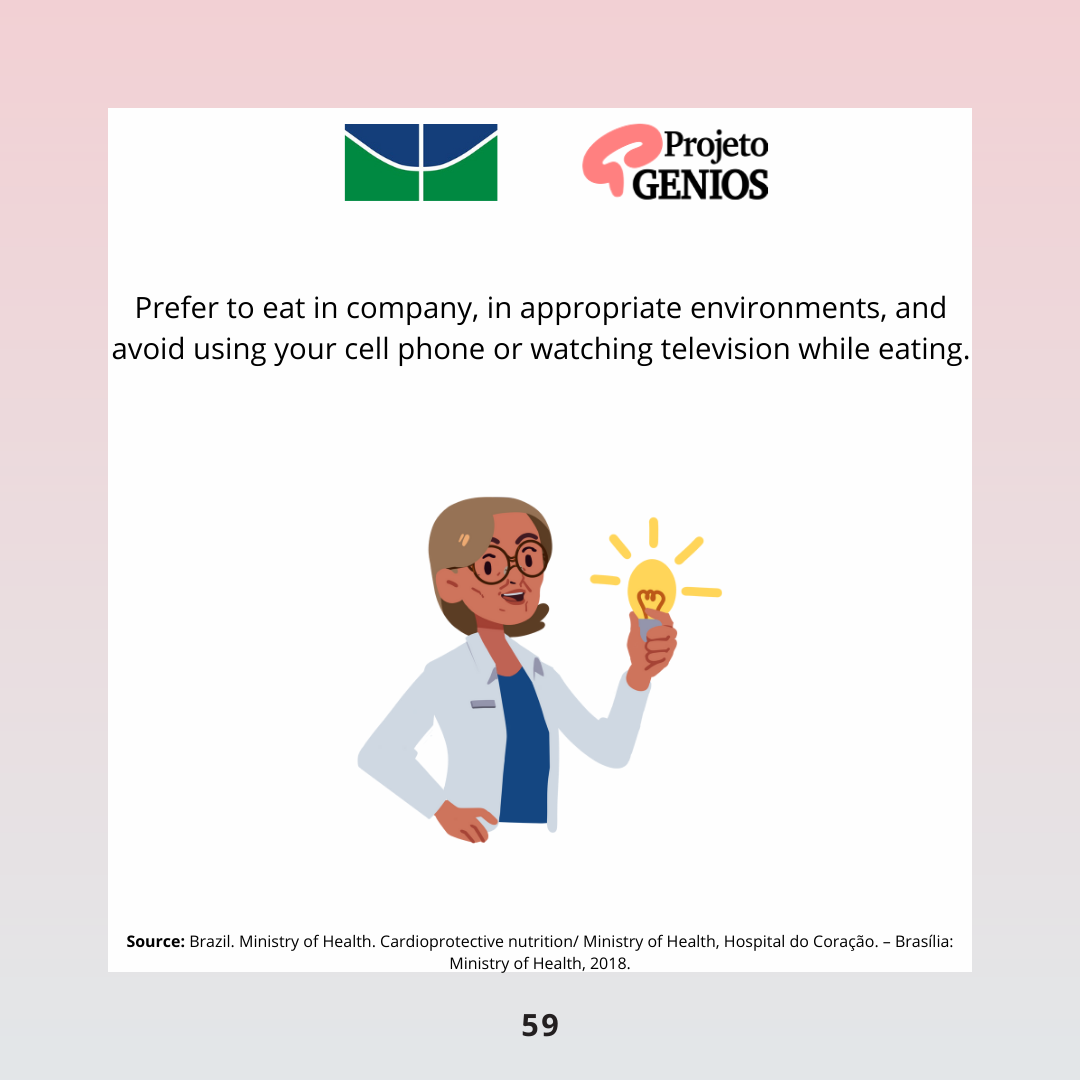

Supplement: Supplementary file 1 [file ijerph-22-00282-s001.zip › Instante Messages English/59.png]

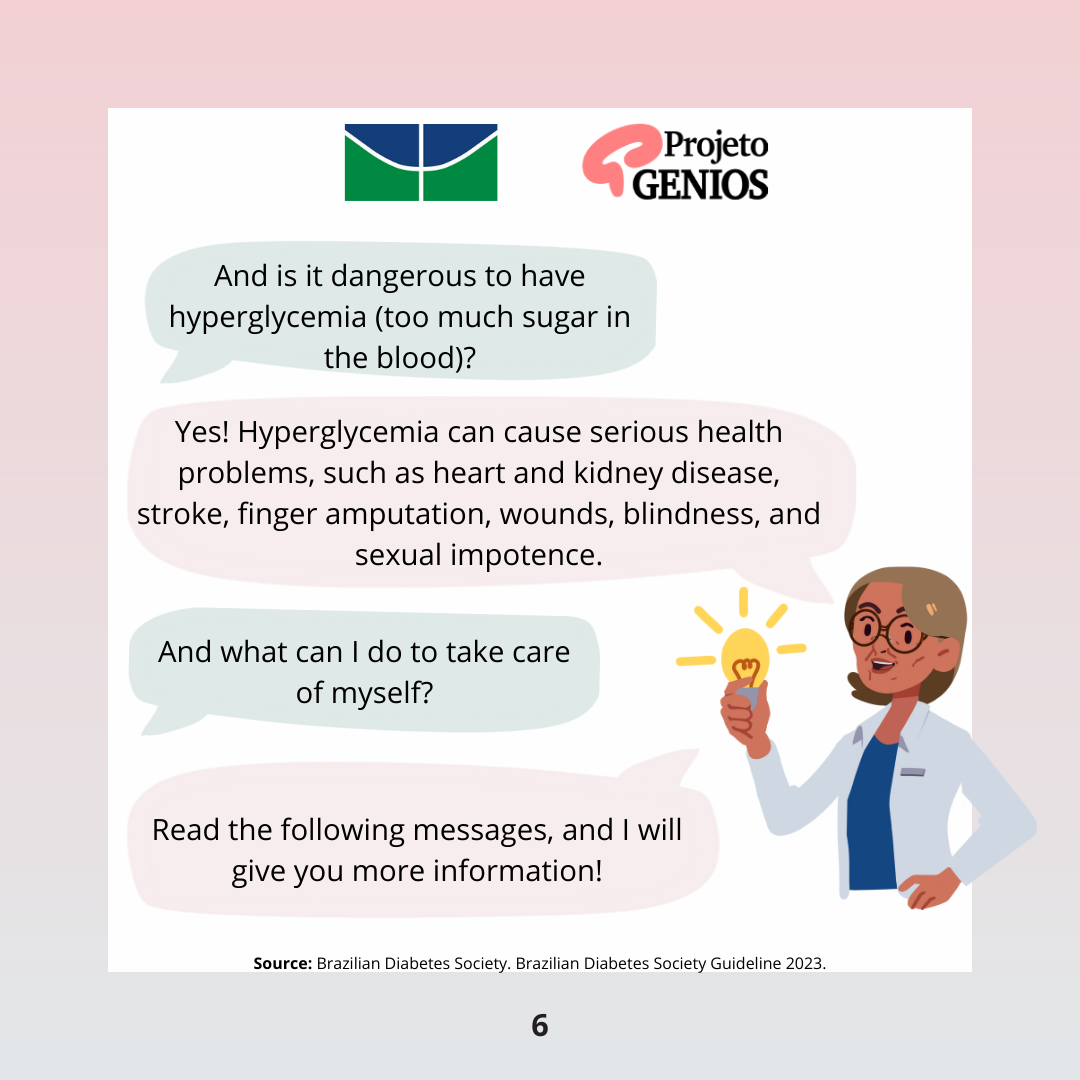

Supplement: Supplementary file 1 [file ijerph-22-00282-s001.zip › Instante Messages English/6.png]

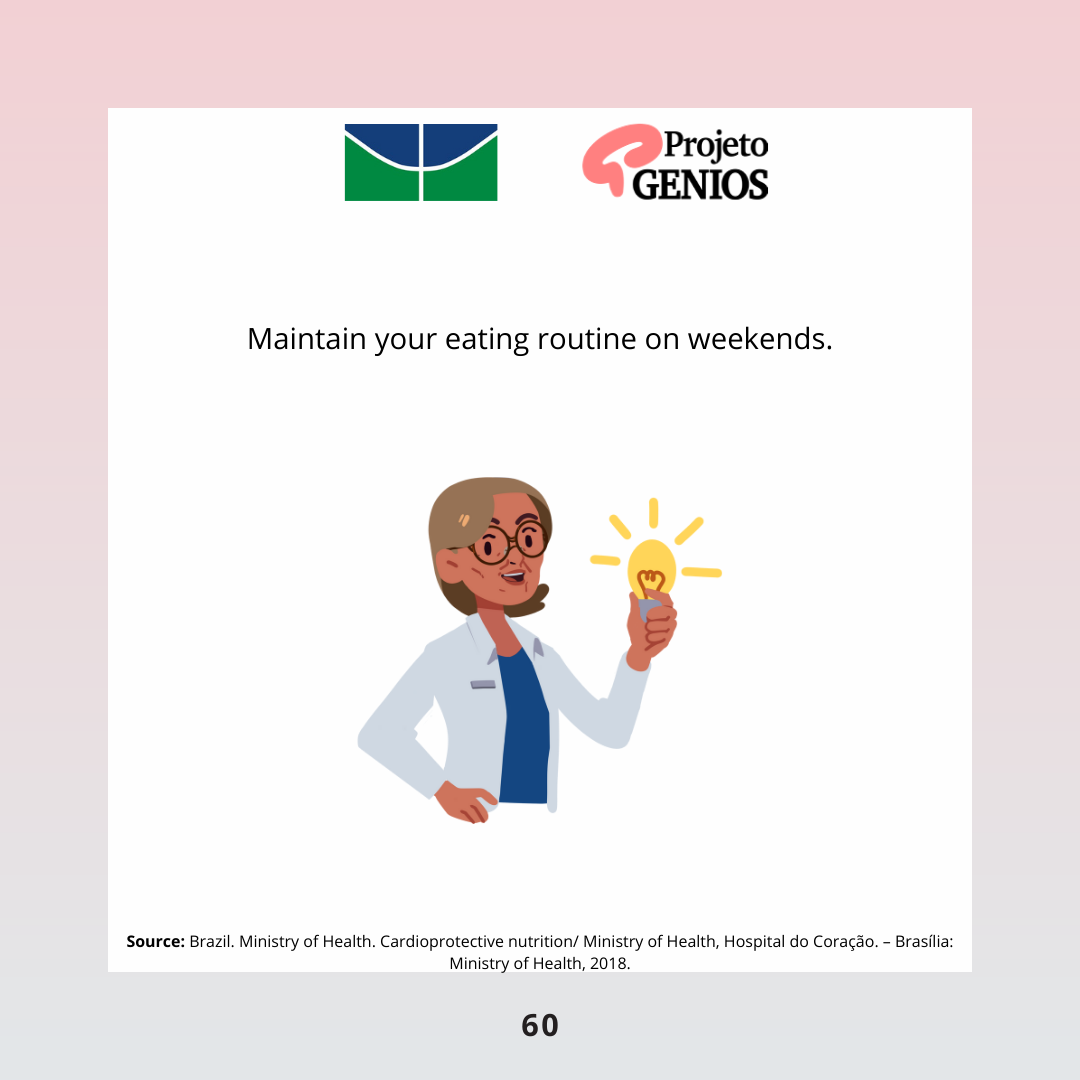

Supplement: Supplementary file 1 [file ijerph-22-00282-s001.zip › Instante Messages English/60.png]

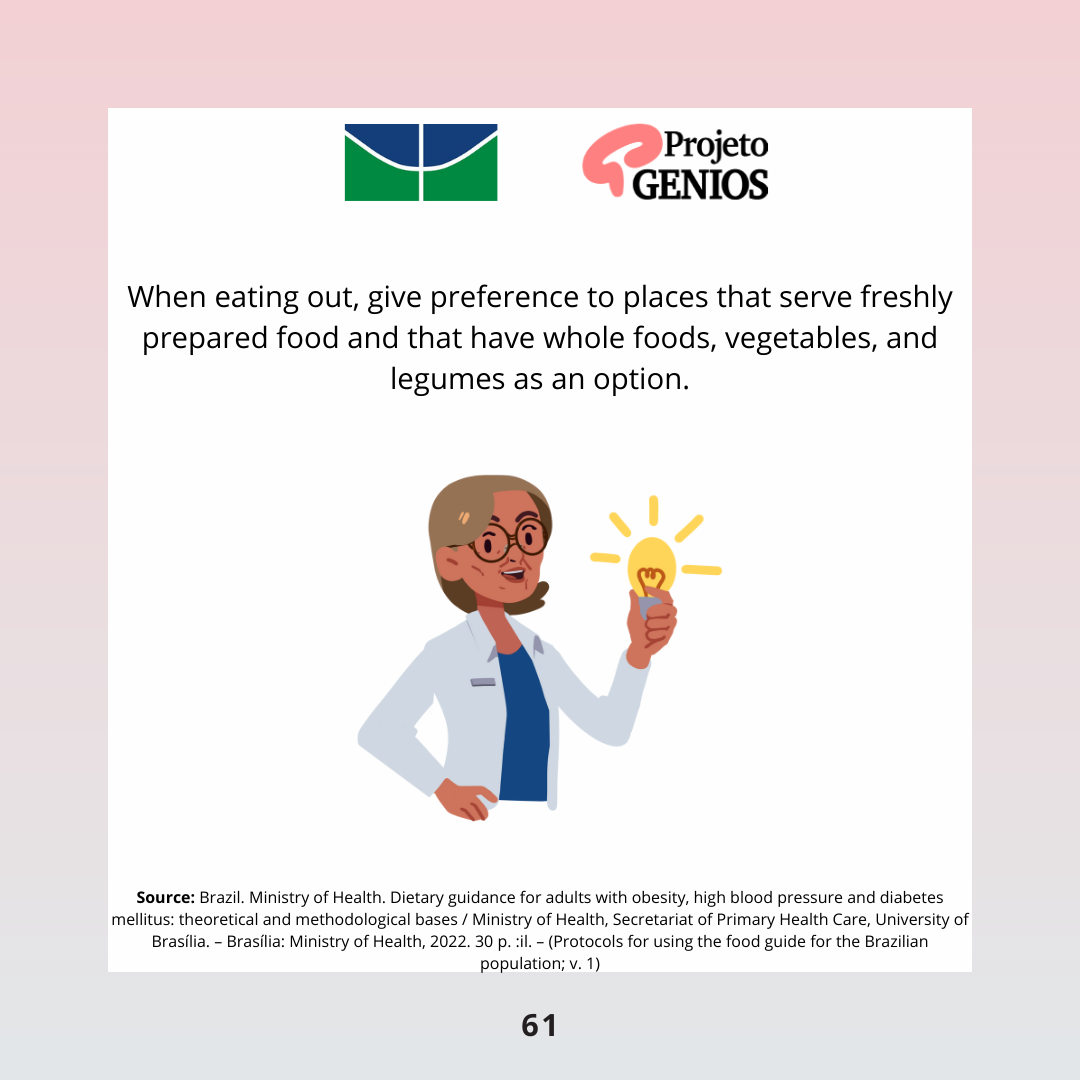

Supplement: Supplementary file 1 [file ijerph-22-00282-s001.zip › Instante Messages English/61.png]

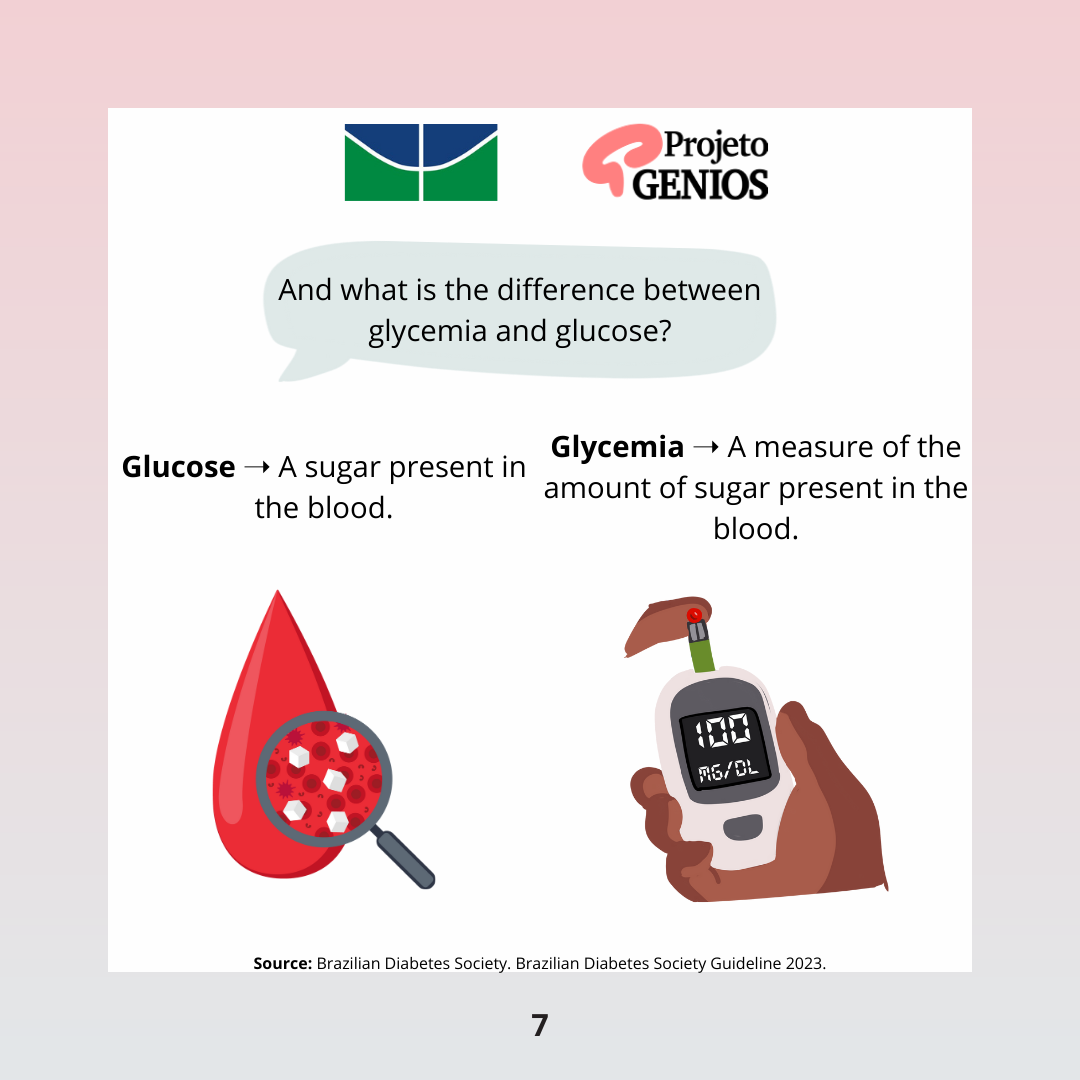

Supplement: Supplementary file 1 [file ijerph-22-00282-s001.zip › Instante Messages English/7.png]

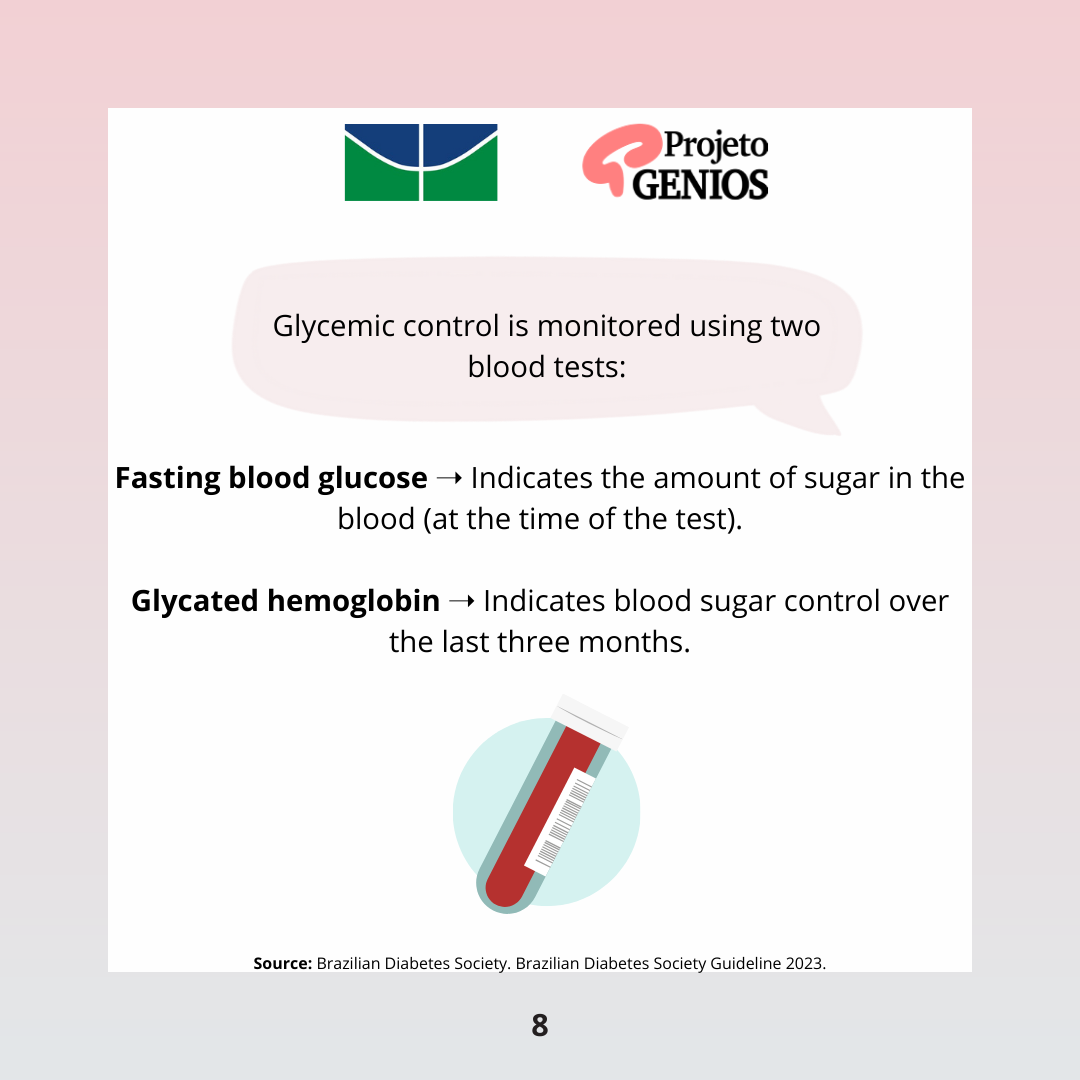

Supplement: Supplementary file 1 [file ijerph-22-00282-s001.zip › Instante Messages English/8.png]

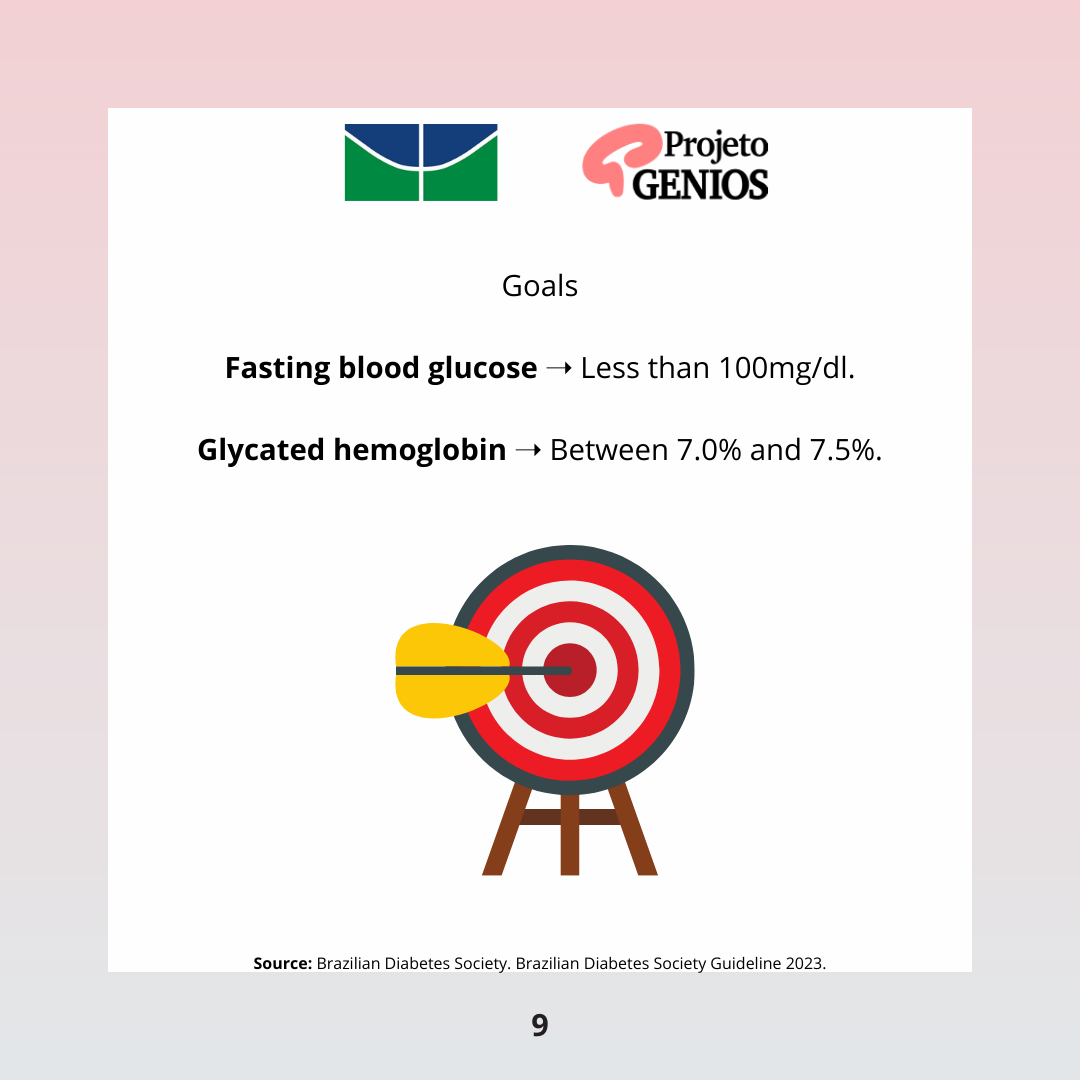

Supplement: Supplementary file 1 [file ijerph-22-00282-s001.zip › Instante Messages English/9.png]
